# General Measurement Tools for Assessing Mental Health Problems Among Children and Adolescents with an Intellectual Disability: A Systematic Review

**DOI:** 10.1007/s10803-021-05419-5

**Published:** 2022-01-12

**Authors:** Marianne Berg Halvorsen, Sissel Berge Helverschou, Brynhildur Axelsdottir, Per Håkan Brøndbo, Monica Martinussen

**Affiliations:** 1 Department of Pediatric Rehabilitation, University Hospital of North Norway, P.O. Box 2, 9038 Tromsø, Norway; 2 NevSom Norwegian Centre of Expertise for Neurodevelopmental Disorders and Hypersomnias, Oslo University Hospital, Oslo, Norway; 3 Regional Centre for Child and Adolescent Mental Health, Eastern and Southern Norway, Oslo, Norway; 4 Department of Psychology, UiT The Arctic University of Norway, Tromsø, Norway; 5 RKBU North, UiT The Arctic University of Norway, Tromsø, Norway

**Keywords:** Assessment, Intellectual disability, Mental disorders, Mental health, Psychometrics

## Abstract

There is a need for more knowledge of valid and standardized measures of mental health problems among children and adolescents with intellectual disability (ID). In this study, we systematically reviewed and evaluated the psychometric properties of instruments used to assess general mental health problems in this population. Following PRISMA guidelines, we reviewed empirical research published from 1980 through February 2020 with an updated search in March 2021 in Medline, Embase, PsycINFO, Health and Psychological Instruments, CINAHL, ERIC, and Web of Science databases. Forty-nine empirical articles were included in this review. Overall, the review indicated consistently better documentation of the reliability and validity of instruments designed for the ID population compared to instruments developed for the general child population.

Cooccurring mental health disorders are more frequent in the intellectual disability (ID) population than in the general population (Einfeld et al., 2011; Munir, 2016). Mental health disorders result in reduced functioning and an increased need for help in everyday life at home, at school, or at work, in addition to difficulties due to ID (Einfeld et al., 2011; Halvorsen et al., 2019). These difficulties are associated with reduced quality of life for the person and the family (Hastings et al., 2001; Lin et al., 2009). Accordingly, careful assessment of mental health should be an essential component of care for all people with ID and should be integrated into clinical practice. The identification of mental health (MH) disorders is, however, considered difficult due to the considerable symptom overlap between ID and MH disorders and the problems of distinguishing between the conditions (Einfeld et al., 2011). Additionally, accompanying communication difficulties and atypical symptom presentations associated with more severe ID make assessment challenging (Stratis & Lecavalier, 2015). The use of relatively broadband standardized instruments is generally recommended in the initial assessment of MH disorders. There are few currently available instruments that have been specifically developed for children and adolescents with ID (e.g., Aberrant Behavior Checklist [ABC]: (Aman & Singh, 1986); Developmental Behavior Checklist [DBC]: (Einfeld & Tonge, 1992), and accordingly, instruments not originally developed for this population are commonly used (e.g., Achenbach System of Empirically Based Assessment [ASEBA]; Strengths and Difficulties Questionnaire [SDQ; Goodman, 1997]). However, there is a need for more knowledge of valid and standardized measures of MH problems among children and adolescents with ID. A previous systematic review evaluated the suitability of MH measures, in terms of psychometric properties (i.e., reliability and validity), that are commonly used for people of all age groups (i.e., children, adolescents, and adults) with severe and profound ID (Flynn et al., 2017). Flynn et al. (2017) found that very few measures were available and recommended (i.e., sound psychometric properties) for adults. Furthermore, they found no eligible studies reporting psychometric properties of instruments for children and adolescents with severe and profound ID. Accordingly, there is an urgent need for more knowledge about the reliability and validity of MH instruments used among children and adolescents across the whole ID spectrum. Such knowledge of measurement properties will provide the clinical and research field with important new knowledge regarding the strengths and weaknesses of these instruments and provide input to further developmental needs in this field.

## Objective

This systematic review aimed to provide an overview of relevant general measures for assessing MH problems among children and youths with ID. More specifically, the research question was the following: What are the psychometric properties of measurement tools used to assess general MH problems in children and adolescents with ID at ages of 4–20 years? We set this age range (i) Because very few MH measurement tools have been developed for children under age four years and particularly for the ID population, and (ii) we wanted mainly children/youth samples because this was the focus of this review and including adults could provide findings that are not necessarily transferable to children.

## Methods

The protocol for this systematic review was registered in PROSPERO, an international register for systematic reviews with health-related outcomes (CRD42020172186). PRISMA guidelines were used for the reporting process (Moher et al., 2009). The PRISMA checklist is available in Appendix I.

### Inclusion and Exclusion Criteria

We included papers if they met the following criteria: (a) at least 70% of the sample in the study were reported as having an intellectual functioning equivalent to a full-scale intelligent quotient (FSIQ) ≤ 80 either by means of a standardized intelligence test or a diagnosis of ID or indirectly by parent report or being a pupil at a special school for children and youths with ID. (b) All studies were based on samples that included children and youths between the mean ages of 4–20 years. Samples reporting participant age above 25 years of age were excluded as the focus on this review were on children and adolescents. (c) Reported original data on quantitative or psychometric outcomes for general MH measures published in a peer-reviewed journal or as a PhD dissertation. (d) Focused on the development, adaptation, or evaluation of a measure of MH. The inclusion criteria for MH problems were derived from the International Statistical Classification of Disease and Related Health Problems, 10th Revision (World Health Organization, 2010). Eligible MH problems and their key diagnostic symptoms, with onset usually occurring during childhood and adolescence, were classified as follows: (a) F20-29: schizophrenia, schizotypal, and delusional disorders; (b) F30-39: mood (affective) disorders; (c) F40-48; neurotic, stress-related and somatoform disorders; and (d) F91-94 behavioral and emotional disorders. Accordingly, we did not include disorders of adult personality and behavior (F60-69), organic mental disorders, disorders due to psychoactive substance abuse, behavioral syndromes associated with physiological disturbances and physical factors, neurodevelopmental disorders (ID, attention-deficit/hyperactivity disorder, autism spectrum disorders, or specific developmental disorders), motor disorders (Tourette syndrome), or other behavioral and emotional disorders with onset usually occurring in childhood and adolescence that are not within F91-94 (e.g., pica or stereotyped movement disorder).

We excluded the following types of papers: (a) Published before 1980 in accordance with Flynn et al. (2017) (b) used specific MH measures with fewer than two symptom domains as the focus in this review was on broadband/general measurement tools, (c) focused on evaluating psychotropic drug interventions, or (d) reported only descriptive mean scores for ID samples (e.g., genetic syndromes) with no other psychometric information.

### Search Methods for Identification of Studies

We searched Medline (Ovid), Embase (Ovid), PsycINFO (Ovid), Health and Psychosocial Instruments (Ovid), CINAHL (EBSCO), ERIC (EBSCO), and Web of Science from 1980 through February 21st, 2020. The trial registers ClinicalTrials.gov and WHO International Clinical Trials Registry Platform (ICTRP) were also searched for ongoing and unpublished trials on May 16th, 2021. An updated search for each included measurement tool was performed on March 13th, 2021.

The search strategy was developed by an information librarian (BA) using a wide range of search terms for intellectual and developmental disabilities, MH issues, children and adolescents, and psychometric properties. No limits were applied to the study design, language, or publication type. The search strategy was adapted to each database (see complete search strategies in Appendix II).

The bibliographies of all included studies and previous systematic reviews were also searched for relevant studies. We contacted experts in the field to identify additional unknown studies; four additional papers were identified in this manner, but none met the inclusion criteria (Brinkley et al., 2007; Kaat et al., 2014; Ono et al., 1996; Siegfrid, 2000).

### Study Selection

All titles and abstracts were independently screened by at least two reviewers (MBH, (screened all references), BA, SBH and MM) in Covidence. All full-text papers were independently screened (always MBH, in addition to SBH, BA, MM, or PHB). Disagreements were resolved by discussion, and if needed, a third author (MM) was consulted to reach a final decision.

### Data Extraction (and Synthesis)

Data were extracted into a table format by one reviewer (MBH or BA) and were checked for accuracy by a second reviewer (SBH or PHB). The extracted data included study design, country, participant demographics (age and sex) and clinical characteristics (i.e., ID severity, adaptive level, comorbid diagnosis), rater characteristics (i.e., parent/caregiver, teacher or other), and information about the data analyses/psychometric properties.

The data were summarized for all the studies reporting on *each measurement tool*, with a narrative synthesis.

### Methodological Quality of MH Measures

As the objective was to assess the psychometric properties of the MH measures as they appeared in the studies we identified, we did not assess the quality of the methods in the included studies themselves. Originally, we planned to use the COSMIN Risk of Bias Checklist (Mokkink et al., 2018) to evaluate the psychometric properties of identified MH measures. However, we found that this tool was more suitable for assessing outcome studies. Accordingly, we chose to use the EFPA review model for the description and evaluation of psychological and educational tests (European Federation of Psychologists' Association (EFPA), 2013) to guide the assessment of the psychometric properties (Table 1). More specifically, reliability (i.e., internal consistency, test–retest reliability, and interrater reliability) and validity (i.e., criterion validity, content validity, and construct validity) were evaluated by means of the interpretation guidelines from the EFPA review model (European Federation of Psychologists' Association (EFPA), 2013) using a four-point scale (0 = not reported/not applicable; 1 = inadequate; 2 = adequate; 3 = excellent/good). We did not evaluate the measure’s reported norms. See Table 1 for more information. This quality assessment was independently conducted by MBH and SBH for 20 randomly chosen studies reporting psychometric statistics. The interrater reliability of these assessments showed an excellent degree of correspondence (*r* = 0.92) for the sum scores. Due to a high degree of correspondence in scoring, the remaining articles/studies (*n* = 29) were then randomly distributed between MBH and SBH. If uncertainty in scoring arose, this was discussed between MBH and SBH; if needed, a third author (MM) was consulted before an agreement was reached.

**Table 1:** Interpretation guidance from the EFPA Review Model (2013) to evaluate the psychometric quality of included measures

	Range	Rating
Sample size	Not reported/applicable *N* < 100 *N* = 100–200 *N* > 200	0 = not reported/applicable 1 = one inadequate study 2 = one adequate study 3 = large/more than one adequate study
Internal consistency: Cronbach’s alpha	Not reported/applicable < .70 = .70–.79 ≥ .80	0 = not reported/applicable 1 = inadequate 2 = adequate 3 = good/excellent
Test–retest/interrater: coefficient	Not reported/applicable < .60 .60–.69 ≥ .70	0 = not reported/applicable 1 = inadequate 2 = adequate 3 = good/excellent
Convergent validity: correlation coefficient	Not reported/applicable < .55 .55–.64 ≥ .65	0 = not reported/applicable 1 = inadequate 2 = adequate 3 = good/excellent
Criterion-related Validity	Not reported/applicable < .20 .20-.34 ≥ .35	0 = not reported/applicable 1 = inadequate 2 = adequate 3 = good/excellent

All studies pertaining to each individual measurement tool were then included *in the overall assessment of each measure*, allowing the authors to establish the weight of evidence for each measure in turn.

## Results

### Literature Selection

**Fig. 1:**
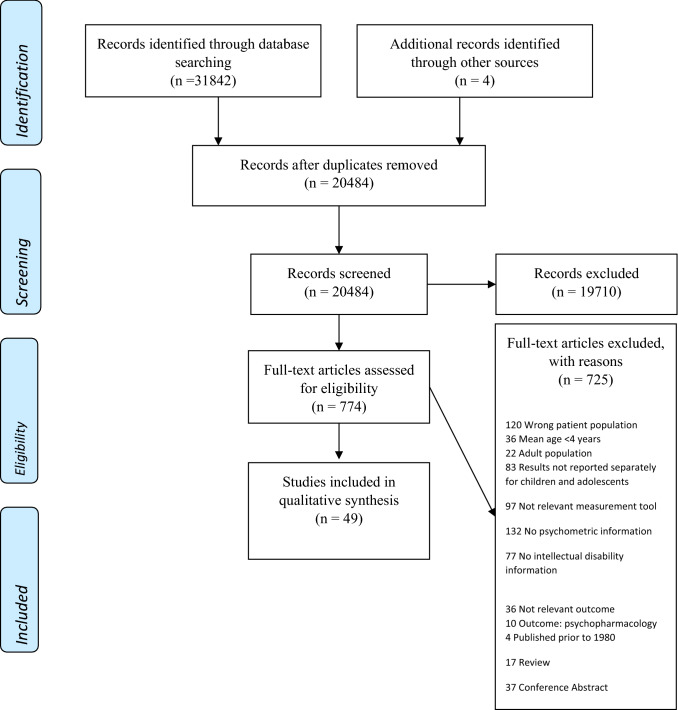
PRISMA Flow Diagram

The literature searches resulted in 22,692 unique references. We excluded 20,069 after screening titles and abstracts, and we assessed 774 full-text articles, of which 725 were excluded (see Appendix III for excluded studies with exclusion reasons). A total of 49 trials/papers were ultimately included. Details of the study selection process and reasons for exclusion are provided in Fig. 1. There were very few cases where a third reviewer (MM) was required to resolve disagreements. We focused on assessment instruments of MH for children and adolescents with chronological ages of 4–20 years. Some assessment tools had additional supporting data for older age groups, but this information was not included in the current review.

### MH Instruments

A total of 49 papers reporting on 10 instruments for assessing MH problems among children and adolescents with ID were identified and included (Aman et al., 1996; Baraldi et al., 2013; Borthwick-Duffy et al., 1997; Bostrom et al., 2016; Braga et al., 2018; Brereton et al., 2006; Brown et al., 2002; Chadwick et al., 2000; Clarke et al., 2003; Coe et al., 1999; Dekker et al., 2002a, 2002b, 2002c; Dieleman et al., 2018; Douma et al., 2006; Einfeld & Tonge, 1995; El-Keshky & Emam, 2015; Embregts et al., 2010; Emerson, 2005; Esbensen et al., 2018; Freund & Reiss, 1991; Hassiotis & Turk, 2012; Hastings et al., 2001; Haynes et al., 2013; Jacola et al., 2014; Kaptein et al., 2008; Koskentausta & Almqvist, 2004; Koskentausta et al., 2004; Marshburn & Aman, 1992; Masi et al., 2002; Matson et al., 1984; Mircea et al., 2010; Murray et al., 2020; Norris & Lecavalier, 2011; Oliver et al., 2003; Oubrahim & Combalbert, 2019; Reiss & Valenti-Hein, 1994; Rice et al., 2018; Rojahn & Helsel, 1991; Rojahn et al., 2010; Sansone et al., 2012; Taffe et al., 2007; Tasse & Lecavalier, 2000; Tasse et al., 1996; Tonge et al., 1996; van Lieshout et al., 1998; Wallander et al., 2006; Wolf, 1981; Wright, 2010) (see Tables 2 and 3). Of these instruments, seven were aimed for the intellectual and developmental disability (IDD) population (i.e., ID instruments), while three instruments were not originally designed or aimed for use in this population (i.e., non-ID instruments) (Table 3).

**Table 2:** Overview of studies: study characteristics and psychometric data

Measure	Author, year	Country	Sample description	IQ/adaptive level	N	Study design	Rater	Psychometric properties
*ABC*								
	Brown et al. (2002)^a^	US	Special education 56% boys. Age range 6–22 years	FSIQ ≤ 80 indexed by school placement	601	Cross-sectional	Parent	Factor structure (EFA/CFA) Internal consistency
	Chadwick et al. (2000)	UK	Special education. 62% boys. Age range 4–11 years	Severe ID defined by means of adaptive level	102	Cross-sectional	Parent (n = 102) Teacher (n = 65)	Interrater agreement Convergent validity (VABS)
	Freund & Reiss, 1991)	US	Outpatients. 69% boys. Age range 3–25 years	Borderline to severe FSIQ: *M* = 53.0 (*SD* = 14.9) Adaptive level: NR	110/94	Cross-sectional	Parent (n = 110) Teacher (n = 94)	Factor structure (EFA) Internal consistency Test–retest Interrater reliability
	Marshburn and Aman (1992)^a^	US	Special education. Gender frequency: NR. Age range 6–21 years	FSIQ ≤ 80. Indexed by school placement FSIQ/adaptive level: NR	666	Cross-sectional	Teacher	Factor structure (EFA). Internal consistency Norms
	Rojahn and Helsel ( 1991)	US	Inpatient psychiatric unit. 75% boys. Age range 3–23 years	Borderline to profound ID FSIQ/adaptive level: NR	199	Follow-up	Direct care staff	Factor structure (EFA) Internal consistency. Interrater reliability Criterion validity
	Sansone et al. (2012)	US	Fragile X. 73% boys. Age range 3–25 years	FSIQ: *M* = 58.0 (*SD* = 18.3)	630	Cross-sectional	Parent/guardian	Factor structure (EFA/CFA)
*ASEBA* CBCL	Borthwick-Duffy et al. (1997)	US	Children with ID. 52% boys. Age range 8–20 years	Mild to profound ID FSIQ: NR	67	Cross-sectional	Parent	Factor structure (EFA)
CBCL	Braga et al. (2018)	Brazil	Williams syndrome 38% boys. Age range 4–6 years	NR	8	Cross-sectional	Parent/caregiver	Convergent validity (BPI-01)
CBCL and TRF	Dekker et al. (2002a)^a^	Netherlands	Special education. 60% boys. Age range 6–18 years Control group from general population	Borderline to moderate ID FSIQ/adaptive level: NR	1041/1855	Follow-up	Parent (CBCL) and teacher (TRF)	Internal consistency (CBCL and TRF) Test–retest (CBCL) Interrater (CBCL and TRF) Convergent validity (DBC)
CBCL	Dieleman et al. (2018)	Belgium/Netherlands	Down syndrome. 55% boys. Age range 4–19 years	NR	67	Cross-sectional	Parent	Internal consistency
YSR	Douma et al. (2006)^a^	Netherlands	Special education. Gender frequency: NR. Age range 11–18 years. Control group from general population (N = 1047)	Borderline to moderate ID FSIQ: *M* = 66.8 (*SD* = 12.1) Adaptive level: NR	281/1047	Longitudinal	YSR and parent CBCL	Internal consistency Interrater. Construct validity (Multitrait-multimethod). Criterion validity
CBCL	Esbensen et al. (2018)	US	Down syndrome. Gender frequency: NR. Age range 6–18 years	IQ: *M* < 48 (*SD* < 11) Adaptive level: NR	88	Cross-sectional	Parent (teacher interrater)	Internal consistency. Interrater. Convergent validity (ABC and NCBRF)
CBCL	Koskentausta et al. (2004)^a^	Finland	Community sample. 61% boys. Age range 6–13 years	Mild to profound ID based on IQ scores/adaptive scores. FSIQ: NR	90	Cross-sectional	Parent/caregiver	Convergent validity (DBC). Criterion validity
CBCL	Jacola et al. (2014)	US	Down syndrome rec. 46% boys. Age range 12–18 years	*IQ: M* = 43.38 (*SD* = 18.29). Adaptive: NR	52	Cross-sectional	Parent/caregiver	Convergent validity (BASC-2 clinical and adaptive scales)
CBCL	Masi et al. (2002)	Italy	ID and concurrent depressive disorder and/or anxiety disorder sample. 58% boys. Age range 11–18 years	Mild to moderate ID. FSIQ: *M* = 56.7 (*SD* = 4.4). Adaptive scores: NR	50	Cross-sectional	Parent	Convergent validity (PIMRA, Zung Depression Scale, and Zung Anxiety Scale)
CBCL	van Lieshout et al. (1998)	Belgium/Netherlands	Prader-Willi Syndrome. 50% boys. Age range 3–20 years	Mild to moderate ID. FSIQ/adaptive: NR	39	Cross-sectional	Parent	Internal consistency
CBCL	Wallander et al. (2006)	Netherlands	Population-based. 60% boys. Age range 6–18 years	Borderline to moderate/severe ID indexed by school placement	968	Prospective	Parent/guardian	Internal consistency Test–retest
TRF	Wright (2010)	US	Special education. 58% boys. Age range 12–18 years	IQ: *M* = 63.70 (*SD* = 9.65) (mild ID)	48	Cross-sectional	Teacher	Internal consistency Convergent/divergent validity (ABI)
*BPC*	Coe et al. (1999)	US	Down syndrome (n = 44). Age range 6–15 years. Control group with non-ID (n = 44)	ID level not reported. VABS total: *M* = 51.4	88	Cross-sectional	Parent, teacher	Interrater
	Matson et al. (1984)	US	Students with ID and control group non-ID. 58% boys. Mean age: 15 years	ID group: mild to moderate ID FSIQ/adaptive: NR	259 (ID)/306 (control)	Cross-sectional	Teacher	Factor structure (EFA)
	Wolf (1981)	US	Special education. 87% boys. Age range 11–16 years	NR	39	Follow-up	Teacher/aid	Interrater Test–retest
*BPI-01*	Baraldi et al. (2013)	Brazil	ID group (n = 30) and non-ID (n = 30). 63% boys. Age range 6–18 years	Normal to moderate ID FSIQ reported. Adaptive: NR	30/30	Cross-sectional	Parent/guardian	Internal consistency. Convergent validity (CBCL)
	Mircea et al. (2010)^a^	Romania	Children with ID. 44% boys. Age range 3–23 years	Mild to profound ID. FSIQ/adaptive: NR	115	Cross-sectional	Caregiver	Internal consistency. Convergent validity (NCBRF)
	Rojahn et al. (2010)^a^	US	Special education. 68% boys. Age range 5–22 years	Mild to profound ID FSIQ/adaptive: NR	237	Follow-up	Parent/guardian (n = 63) Teacher (n = 27)	Factor structure (CFA) Internal consistency. Test–retest Interrater
	Oubrahim and Combalbert (2019)	France	Specialized institutions. 49% boys. Age range 7–24 years	Mild to profound ID. IQ level based clinical records. FSIQ/adaptive: NR	305	Cross-sectional	Care staff	Factor structure (CFA). Internal consistency. Interrater
*CBI*	Oliver et al., (2003))	UK	Child sample from schools with severe ID. 68% boys. Age range 4–12 years	Severe ID FSIQ/adaptive score: NR	47	Cross-sectional	Teacher	Content validity Convergent validity (ABC)
*DBC-P*	Brereton et al. (2006)	Australia	ASD sample. 85% boys. Age range 3–24 years ID sample: 58% boys. Age range 4–18 years	ASD sample: normal to severe ID ID sample: borderline to profound ID Formal IQ test. FSIQ/adaptive: NR	381 (ASD)/550 (ID)	Cross-sectional	Parent/caregiver	Criterion-related validity
	Clark et al. (2003)	Australia	Unselected patients with ID attending psychiatric clinic on at least two occasions. Gender/age: NR	NR	37	Follow-up	Parent	Criterion-related validity
	Dekker et al. (2002a)^a^	Australia/Netherlands	Combined sample: epidemiological prevalence study/nonresidential school, daycare center. 59% boys. Age range 3–22 years	Mild to profound ID indexed from school/daycare placement	1536	Cross-sectional	Parent and teacher	Factor structure (EFA) Internal consistency
	Dekker et al. (2002a)^a^	Netherlands	Nonresidential school settings/special education/day centers. 60% boys. Age range 6–18 years	IQ from < = 80. Borderline to profound ID, indexed from placement	1057/930	Cross-sectional/follow-up	Parents (n = 1057) Teachers (n = 930) Both (n = 851)	Internal consistency Test–retest. Interrater Convergent validity (CBCL) Divergent validity (Vineland) Criterion validity
	Einfeld and Tonge (1995)	Australia	Population-based study. 60% boys. Age range 4–18 years	Mild to profound ID. FSIQ/adaptive score: NR	1093	Cross-sectional/follow-up	Parent/caregiver	Content validity. Factor structure (EFA). Internal consistency. Test–retest- Interrater. Convergent validity. Criterion validity
	Hassiotis and Turk (2012)	UK	Clinical service sample: 64% boys. Age range 12–19 years	Mild to profound ID FSIQ: NR VABS total: *M* = 35 (*SD* = 16)	75	Cross-sectional	Parent and teacher	Criterion validity (agreement ICD-10 diagnoses)
	Hastings et al. (2001)	UK	Special education. 68% boys. Age range 4–18 years	High proportion likely severe to profound ID. FSIQ/adaptive score: NR	531	Cross-sectional	Parent and teacher	Factor structure (EFA). Internal consistency
	Koskentausta and Almqvist (2004)^a^	Finland	Community sample. 63% boys. Age range 6–13 years	Mild to profound ID based on IQ scores/adaptive scores. FSIQ: NR	85	Cross-sectional	Parent	Interrater. Convergent validity (ABS total Maladaptive Behavior). Criterion validity
*DBC-P24/short form*	Taffe et al. (2007)	Australia/other countries for cross-validation	Epidemiological ID sample. Cross-validation samples from England, Finland, and the Netherlands in addition to genetic syndrome subgroups	Mild to profound ID. FSIQ/adaptive score: NR	51–1057	Longitudinal/cross-sectional data from first three waves	Parent/caregiver	Criterion validity
	Tonge et al. (1996)	Australia	Community epidemiological prevalence study. > 50% boys. Age range 4–20 years.Community sample (n = 450). Validation sample (n = 448)	Mild to profound ID.FSIQ/adaptive score: NR	1093/450/448	Cross-sectional	Parent/caregiver	Factor structure (EFA). Internal consistency
*NCBRF: Problem Behavior section* Parent/teacher versions	Aman et al. (1996)^a^	US	Outpatients. 65% boys. Age range 3–16 years	Majority IQ range 55–70. Adaptive: NR	326/260	Cross-sectional	Parent (n = 326) and teacher (n = 260)	Content validity. Factor structure (EFA). Internal consistency. Interrater. Convergent validity (ABC)
	Mircea et al. (2010)^a^	Romania	Children with ID noninstitutional housing. 44% boys. Age range 3–23 years	Mild to profound ID FSIQ/adaptive: NR	115	Cross-sectional	Teacher	Internal consistency Convergent validity (BPI-01)
	Norris and Lecavalier ( 2011)	US	Special education/outpatients. 64% boys. Age range 5–18 years	Borderline to profound ID FSIQ/adaptive: NR	399	Cross-sectional	Parent/caregiver	Factor structure (CFA) Internal consistency Convergent validity (DBC) Criterion validity
	Rojahn et al. (2010)^a^	US	Special education. 68% boys. Age range 4–22 years	Mild to profound ID FSIQ/adaptive: NR	237	Cross-sectional/test–retest	Parent/guardians (n = 63) Teacher (n = 27 for interrater; n = 24 test/retest)	Factor structure (CFA) Internal consistency Test–retest Interrater reliability (teacher-teacher/teacher-parent) Convergent validity (BPI-01)
	Tasse et al. (1996)^a^	US	See Aman et al. (1996) above. Identical sample					Norms
	Tasse and Lecavalier (2000)	Canada/French version	Students. 62% boys. Age range 4–18 years	Mild to profound ID FSIQ: NR	109	Cross-sectional	Parent/teacher	Interrater
*Reiss Scales*	Reiss and Valenti-Hein (1994)	US	Two independent community samples with children and adolescents with ID. Sample 1: 61% boys. 78% under 11 years. Sample 2: 60% boys. Age range 4–21 years	Mild to profound ID FSIQ/adaptive: NR	313/270	Cross-sectional	Parent/caretaker or teacher	Content validity Factor structure (EFA) Internal consistency Criterion validity (diagnosis)
*SDQ*	El-Keshky and Emam (2015)	Saudi Arabia/Oman	Students with learning disability. Mean age: 8 years	IQ below 80 indexed by school placement. FSIQ/adaptive: NR	323 (SA)/229 (Omani)	Cross-sectional	Teacher	Factor structure (CFA). Internal consistency
	Embregts et al. (2010)	Netherlands	60% boys. Age range 12–16 years	Mild ID FSIQ/adaptive: NR	45		Self-report Parent Teacher	Internal consistency
	Emerson, 2005)	UK	Population-based sample children with/without ID. 77% boys, ID sample. Age range 11–15 years	NR	98 (ID)/4074 (control)	Cross-sectional	Self-report. Parent. Teacher	Internal consistency. Interrater. Criterion validity
	Haynes et al. (2013)	Australia	Children with ID from schools. 63% boys. Age range 9–14 years	Mild ID. Formal IQ/adaptive tests. FSIQ/adaptive: NR	128	Cross-sectional	Self-report	Content validity Factor structure (EFA/CFA) Internal consistency
	Kaptein et al. (2008)	Netherlands	Special education. 59% boys. Age range 6–12 years Non-ID control group (n = 707)	Mild to moderate ID FSIQ/adaptive: NR	260/707	Cross-sectional	Parent	Internal consistency Criterion validity
	Murray et al. (2020)	UK	Cohort children with ID. 68% boys. Age range 4–15 years	FSIQ: NR. VABS-II total scores: M = 58	626	Cross-sectional	Parent	Convergent validity (DBC)
	Rice et al. (2018)	Australia/UK	Children with ID from mental health clinics/Down syndrome. 66% boys. Age range 4–17 years	NR	83	Cross-sectional	Parent	Convergent validity (DBC)
*WellSEQ*	Bostrøm et al. (2016)	Sweden	Special education. 71% boys. Age range 12–16 years	NR	113/67 (parents)/97 (teacher ratings)	Cross-sectional/parent	Self-report. Parent. Teacher	Content validity. Internal consistency. Test–retest. Interrater. Convergent validity (SDQ)

*ABC*  Aberrant Behavior Checklist, *ABI*  Adaptive Behavior Inventory, *ASEBA* Achenbach System of Empirically Based Assessment; *BPC*  Behavior Problem Checklist, *BPI-01*  Behavior Problem Inventory, *CBCL *Child Behavior Checklist, *CBI*  Challenging Behavior Interview, *DBC* Developmental Behavior Checklist, *FSIQ*  full-scale IQ, *NCBRF*  Nisonger Child Behavior Rating Form, *NR*  not reported, *Reiss Scales * Reiss Scales for Children's Dual Diagnosis, *SDQ*  Strengths and Difficulties Questionnaire, *TFR*  Teacher Rating Form, *VABS*  Vineland Adaptive Behavior Scales, *YSR* Youth Self Report, *WellSEO* Well-being in Special Education Questionnaire

^a^Report data from the same study

**Table 3:** Description of included instruments from all studies

Instrument	Purpose/ composition	Administration and scoring	Framework
*ID instruments*			
*Aberrant Behavior Checklist-Community version* (*ABC-C*; Aman & Singh, 1986, 2017)	58 items, 5 subscales: Irritability, Social Withdrawal, Stereotypic Behavior, Hyperactivity, Non-Compliance, Inappropriate Speech	Proxy 4-point scale (0–3)	Empirically developed
*Behavior Problems Inventory* (*BPI*-*01*; Rojahn et al. (2001)	49 items, 3 subscales: Self-Injurious Behavior, Stereotyped Behavior, Aggressive/Destructive Behavior	Proxy 5-point frequency scale (0–4) 3-point severity scale (0–3)	Empirically developed
*Challenging Behavior Interview* (*CBI*; Oliver et al., 2003)	Two parts. Part I identifies the occurrence of 5 forms of behaviors: Self-Injury, Physical Aggression, Verbal Aggression, Disruption of the Environment, Inappropriate Vocalizations. Part II: 14 subscales measuring the frequency, duration and implication of the episodes	Proxy Part II: 4- or 5-point scale (e.g., 1–5)	Definitions of challenging behavior (e.g., Emerson, 1998) underscoring the need to include a broad assessment of its impact. Review of the literature on challenging behavior assessment instruments
*Developmental Behavior Checklist* (*DBC*; Einfeld & Tonge, 1992)	96 items, 6 subscales: Disruptive, Self-Absorbed, Communication Disturbance, Anxiety, Social Relating, Antisocial Total Behavior Problem Score	Proxy: Primary carer and teacher versions 3-point scale (0–2)	Empirically developed
*Nisonger CBRF Problem Behavior Section* (*Nisonger*; Aman et al., 1996)	60 items; 6 subscales: Conduct Problems, Insecure/anxious, Hyperactivity; Self-Injury/Stereotypic, Self-Isolated/Ritualistic, Overly Sensitive	Proxy: Parent and teacher versions 4-point scale (0–3)	Adapted/modified the Child Behavior Rating Form^a^ . Empirically developed
*Reiss Scales for Children´s Dual Diagnosis* (*Reiss;* Reiss & Veletin-Hein, 1994)	60 items, 10 subscales: Anger/Self-Control, Anxiety Disorder, Attention Deficit, Autism, Conduct Disorder, Depression, Poor Self-Esteem, psychosis, Somatoform Behavior, Withdrawn/Isolated	Proxy 3-point scale (0–2)	Empirically developed. Adapted from the adult version
*Well-Being in Special Education Questionnaire* (*WellSEQ*; Bostrom et al., 2016)	42 items^b^. 5 scales: Mental Health, Mental Ill-Health, Family Relations, School Environment, Peer Relations and Conflict	Youth Self-Report and proxy: Parent and teacher versions 3-point scale (0–2)	Literature review, expert consultations and workshops with special education students
*Non-ID instruments*			
*Behavior Problem Checklist* (*BPC*; Quay & Peterson, 1975, 1983)	77 items, 6 subscales: Conduct Disorder, Socialized Aggression, Attention Problem-Immaturity; Anxiety-Withdrawal, Psychotic Behavior, Motor Excess An additional set of 12 items do not load on any factor but largely assess Social Withdrawal	Proxy 3-point scale (0–2)	Empirically developed
*ASEBA: Child Behavior Checklist* (*CBCL*; Achenbach, 1991; Achenbach & Rescorla, 2001)	120 items, 8 syndrome subscales: Withdrawn, Somatic Complaints, Anxious/Depressed, Social Problems, Thought Problems, Attention Problems, Delinquent behavior, Aggressive Behavior 2 broadband scales: Internalizing and Externalizing. A total problems score The 2001 version includes in addition 6 DSM-oriented subscales: Affective Problems, Anxiety Problems, Somatic Problems, Attention Deficit/Hyperactivity Problems, Oppositional Defiant Problems, Conduct problems	Youth self-report Proxy: Parent report and teacher report forms 3-point scale (0–2)	Empirically developed DSM-oriented scales (latest version)
*Strengths and Difficulties Questionnaire* (*SDQ*; Goodman, 1997, 1999)	25 items, 5 subscales: Conduct Problems, Emotional problems, Hyperactivity/Inattention, Peer Relationships, Prosocial Behavior 2 broadband scales: Internalizing and Externalizing A total difficulties score Extended version includes Impact Supplement: 6 items	Youth self-report version Proxy: Parent and teacher versions 3-point scale (0–2) 4-point scale (0–3)	Modified the Rutter Questionnaire. Empirically developed

Parent/primary caregiver versions of the instruments, where they exist, are described as other versions of the instruments overlap to a very large extent

^a^Edelbrock (1985)

^b^Self-report version

The included assessment instruments were intended to screen for a relatively broad spectrum of problems, so-called broadband assessment instruments. The frequency, severity and duration of target behaviors were most often used to measure MH problems. Four papers gave a first report of the development or adaptation of a new instrument (Challenging Behavior Interview [CBI] (Oliver et al., 2003); Developmental Behavior Checklist [DBC] (Einfeld & Tonge, 1995); Nisonger Child Behavior Rating Form [NCBRF] (Aman et al., 1996); Reiss Scales for Children’s Dual Diagnosis [Reiss] (Reiss & Valenti-Hein, 1994); Well-Being in Special Education Questionnaire [WellSEQ] (Bostrom et al., 2016)).

Overall, the identified instruments reported their development/framework through a widely defined bottom-up approach (i.e., descriptive-empirical approach) based on specific descriptors of children’s functioning. These individual symptoms (i.e., items) were either based on other existing questionnaires (e.g., the NCBRF was adapted from the Child Behavior Rating Form, and the SDQ was adapted from the Rutter Questionnaire) and/or based on a literature review of the field, expert consultation, or case files from IDD services (i.e., the ID instruments). The ASEBA and the latest version (e.g., Child Behavior Checklist [CBCL]) specifically reported six additional subscales based on the Diagnostic and Statistical Manual of Mental Disorders (DSM) (Achenbach & Rescorla, 2001). The ASEBA (i.e., CBCL, Teacher Rating Form [TRF], and Youth Self Report [YSR]) was the most comprehensive measure identified in terms of the number of items (i.e., 120 items) compared to the other measures (mean number of 53 items). The SDQ, on the other hand, lacked DSM-oriented subscales.

It is noteworthy that the majority of the instruments were proxy or informant-based measures with the exception of the WellSEQ (Bostrom et al., 2016), an ID instrument, and the ASEBA and SDQ, both non-ID instruments, which also offered a youth self-report form. Five papers reported using the youth self-report form (ASEBA: (Douma et al., 2006); SDQ: (Embregts et al., 2010; Emerson, 2005; Haynes et al., 2013); WellSEQ: (Bostrom et al., 2016). The other papers reported using parent/primary caregiver, teacher, and (health) care staff as informants (Table 2). All identified studies/papers reporting on instruments were in the English language. Moreover, all instruments were originally developed in English, with the exception of the WellSEQ (Bostrom et al., 2016), which was developed in the Swedish language. However, the majority of the identified measures had one or more studies that reported psychometric properties for non-English versions, with the exception of the ABC (Aman & Singh, 1986) and Behavior Problem Checklist (BPC) (Quay & Peterson, 1975, 1983) (see Table 2).

We found that most papers used the ASEBA (11 papers) followed by the DBC (10 papers) and further followed by, in descending order, the SDQ (7 papers), ABC/NCBRF (6 papers each), Behavior Problems Inventory-01 (BPI-01) (4 papers), BPC (3 papers), and CBI/Reiss/WellSEQ (1 paper each). For seven of the measures, the researcher by whom it was developed was involved in its evaluation (ABC: Brown et al., 2002; Marshburn & Aman, 1992; BPI-01: Baraldi et al., 2013; Mircea et al., 2010; Rojahn et al., 2010; CBI: Oliver et al., 2003; DBC: Brereton et al., 2006; Clark et al., 2003; Dekker et al., 2002a, b, c; Einfeld & Tonge, 1995; Taffe et al., 2007; Tong et al., 1996; NCBRF: Aman et al., 1996; Tasse et al., 1996; Rojahn et al., 2010; Tasse & Lecavalier, 2000; Reiss: Reiss & Valentin-Hein, 1994; WellSEQ: Bostrom et al., 2016).

In relation to participant samples, the vast majority included mixed samples of people with ID with the exception of pure syndrome-specific samples in alphabetical order: i) Down syndrome (Coe et al., 1999; Dieleman et al., 2018; Esbensen et al., 2018; Jacola et al., 2014); ii) Fragile X (Sansone et al., 2012), iii) Prader-Willi syndrome (van Lieshout et al., 1998) and iiii) Williams syndrome (Braga et al., 2018). The majority of the studies included samples, in which the major proportion were reported with up to a moderate ID level, with the exception of a few studies reporting a high proportion of likely more severe ID (Chadwick et al., 2000; Hastings et al., 2001; Oliver et al., 2003). It should be noted, as shown in Table 2, that in general, very few studies reported formal IQ data and/or data concerning participants’ adaptive function level. FSIQ was reported only with the ABC (two papers), ASEBA (five papers), and BPI-01/NCBRF (one paper each). In relation to sex, overall, the papers reported on samples consisting of a higher proportion of boys, with the exception of five studies (ASEBA: Braga et al., 2018; Jacola et al., 2014); BPI-01 (Mircea et al., 2010; Oubrahim & Combalbert, 2019); NCBRF: (Mircea et al., 2010)). Moreover, population-based samples were reported by six papers (ASEBA: (Wallander et al., 2006); DBC: (Dekker et al., 2002a, b, c; Einfeld & Tonge, 1995; Taffe et al., 2007; Tonge et al., 1996); SDQ: (Emerson, 2005)), and special education/school samples were reported by 19 papers (ABC: Brown et al., 2002; Chadwick et al., 2000; Marshburn & Aman, 1992); ASEBA: (Dekker et al., 2002a, b, c; Douma et al., 2006; Wright, 2010); BPC: (Matson et al., 1984; Wolf, 1981); BPI-01: (Rojahn et al., 2010); CBI: (Dekker et al., 2002a, b, c; Oliver et al., 2003); DBC: (Hastings et al., 2001); NCBRF: (Norris & Lecavalier, 2011; Rojahn et al., 2010; Tasse & Lecavalier, 2000); SDQ: (El-Keshky & Emam, 2015; Haynes et al., 2013; Kaptein et al., 2008); WellSEQ: (Bostrom et al., 2016)). The remaining papers reported on some form of community samples, specific syndrome samples (as noted above) or patient samples (Table 2). Regarding sample sizes, 30 papers reported a sample size of 100 participants or more, as shown in Table 2.

### Methodological Quality of MH Measures

The quality assessment of the psychometric properties in terms of reliability and validity of the MH measures as they appeared in the papers/studies indicated overall summary scores ranging from 0% (i.e., the relevant properties not reported; (Tasse & Lecavalier, 2000)) to 89% (i.e., the majority of the properties documented in large sample and found to be good/excellent properties; (Einfeld & Tonge, 1995)) (Table 4).

**Table 4:** Quality assessment of instruments

Instrument/study	Internal consistency Max score: 6		Test–retest reliability Max score: 6		Interrater reliability Max score: 6		Criterion validity Max score: 6		Content validity Max score: 3	Construct validity Max score: 9			Sum score/%
	Sample (*N*) size	Size of coeffic	*N* size	Size of coeffic	*N* size	Size of coeffic	*N* size	Size of coeffic	Content validity	*N* size	Factor analysis	Correlation similar constructs	
*ABC-C*													
Brown et al. (2002)	3	3	0	0	0	0	0	0	0	3	2	0	11/31%
Chadwick et al. (2000)	0	0	0	0	1	2	0	0	0	1	0	2	6/17%
Freund & Reiss, 1991. *Parent*	2	3	1	3	1	1	0	0	0	2	3	0	16/44%
*Teacher*	2	3	1	2	1	1	0	0	0	2	3	0	15/42%
Marshburn & Aman, 1992)	3	3	0	0	0	0	0	0	0	3	2	0	11/31%
Rojahn & Helsel, 1991)	2	3	0	0	2	1	2	3	0	2	2	0	17/47%
Sansone et al. (2012)	0	0	0	0	0	0	0	0	0	3	1	0	4/11%
**ABC Total Score**													80
*ASEBA*													
Borthwick-Duffy et al. (1997)	0	0	0	0	0	0	0	0	0	1	2	0	3/8%
Braga et al. (2018)	0	0	0	0	0	0	0	0	0	1	0	3	4/11%
Dekker et al. (2002a, b, c)													
*Parent*	3	2	3	3	3	1	0	0	0	3	0	3	21/58%
*Teacher*	3	2	0	0	3	1	0	0	0	3	0	3	15/42%
Dieleman et al. (2018)	1	2	0	0	0	0	0	0	0	0	0	0	3/8%
Douma et al. (2006)	3	2	0	0	3	1	3	2	0	3	0	1	18/50%
Esbensen et al. (2018)	1	2	0	0	1	1	0	0	0	1	0	1	7/19%
Koskentausta et al. (2004)	0	0	0	0	0	0	1	1	0	1	0	3	6/17%
Jacola et al. (2014) Masi et al. (2002)	0 0	0 0	0 0	0 0	0 0	0 0	0 0	0 0	0 0	1 1	0 0	2 2	3/8% 3/8%
Van Lieshout et al. (1998)	1	2	0	0	0	0	0	0	0	0	0	0	3/8%
Wallander et al. (2006)	3	3	3	3	0	0	0	0	0	0	0	0	12/33%
Wright, 2010)	1	3	0	0	0	0	0	0	0	1	0	3	8/22%
**ASEBA Total**													106
*BPC*													
Coe et al. (1999) Matson et al. (1984)	0 0	0 0	0 0	0 0	1 0	1 0	0 0	0 0	0 0	0 3	0 1	0 0	2/6% 4/11%
Wolf, 1981)	0	0	1	1	1	1	0	0	0	0	0	0	4/11%
**BPC Total**													10
*BPI-01*													
Baraldi et al. (2013)	1	2	0	0	0	0	0	0	0	1	0	2	6/17%
Mircea et al. (2010)	2	3	0	0	0	0	0	0	0	2	0	3	10/28%
Rojahn et al. (2010)	3	2	1	3	1	2	0	0	0	3	1	3	19/53%
Oubrahim & Combalbe, 2019)	3	2	0	0	1	3	0	0	0	3	3	0	15/42%
**BPI-01 Total**													50
*CBI*													
Oliver et al. (2003)	0	0	0	0	0	0	0	0	3	1	0	2	6/17%
CBI Total													6
*DBC*													
Brereton et al. (2006)	0	0	0	0	0	0	3	3	0	0	0	0	6/17%
Clark et al. (2003)	0	0	0	0	0	0	1	2	0	0	0	0	3/8%
Dekker et al. (2002b)													
*Parent*	3	2	0	0	0	0	0	0	0	3	3	0	11/31%
*Teacher*	3	2	0	0	0	0	0	0	0	3	3	0	11/31%
Dekker et al. (2002c)													
*Parent*	3	2	1	2	1	3	3	3	0	3	0	3	24/67%
*Teacher*	3	2	1	3	1	3	3	3	0	3	0	3	25/69%
Einfeld & Tonge, 1995)	3	2	3	3	3	3	1	3	3	3	2	3	32/89%
Hassiotis & Turk, 2012)	0	0	0	0	0	0	1	2	0	0	0	0	3/8%
Hastings et al. (2001)	3	2	0	0	0	0	0	0	0	3	2	0	10/28%
Koskentausta & Almqvist, 2004)	0	0	0	0	1	2	2	1	0	2	0	3	11/31%
Tonge et al. (1996)	3	2	0	0	0	0	0	0	0	3	2	0	10/28%
Taffe et al. (2007)	0	0	0	0	0	0	3	3	0	0	0	0	6/17%
**DBC Total**													152
*NCBRF*													
Aman et al. (1996)													
*Parent*	3	3	0	0	2	1	0	0	3	3	3	3	21/58%
*Teacher*	3	3	0	0	2	1	0	0	3	3	3	0	18/58%
Mircea et al. (2010)	2	3	0	0	0	0	0	0	0	2	0	3	10/28%
Norris & Lecavalier, 2011)	3	3	0	0	0	0	1	2	0	3	2	2	16/44%
Rojahn et al. (2010)	3	2	1	3	1	2	0	0	0	3	1	3	19/53%
Tasse et al. (1996) *Parent*	0	0	0	0	2	1	0	0	0	0	0	0	3/8%
*Teacher*	0	0	0	0	2	1	0	0	0	0	0	0	3/8%
Tasse & Lecavalier, 2000)	0	0	0	0	0	0	0	0	0	0	0	0	0/0%
**NCBRF Total**													90
*Reiss Scales*													
Reiss & Valentin-Hein, 1994)	3	2	0	0	0	0	1	1	3	3	3	0	16/44%
**Reiss Total**													16
*SDQ*													
El-Keshky & Emam, 2015)	3	2	0	0	0	0	0	0	0	3	2	0	10/28%
Embregts et al. (2010) *Parent*	1	2	0	0	0	0	0	0	0	0	0	0	3/8%
*Teacher*	1	2	0	0	0	0	0	0	0	0	0	0	3/8%
*Self-report*	1	1	0	0	0	0	0	0	0	0	0	0	2/6%
Emerson, 2005)	1	1	0	0	1	1	1	1	0	0	0	0	6/17%
Haynes et al. (2013)	2	1	0	0	0	0	0	0	1	2	1	0	7/19%
Kaptein et al. (2008)	3	2	0	0	0	0	3	3	0	0	0	0	11/31%
Murray et al. (2020)	0	0	0	0	0	0	0	0	0	3	0	2	5/14%
Rice et al. (2018)	0	0	0	0	0	0	0	0	0	1	0	2	3/8%
**SDQ Total**													50
*WellSEQ*													
Bostrom et al. (2016)	2	2	1	2	1	1	0	0	3	1	0	2	15/42%
**WellSEQ Total**													15

Quality sum score (possible range 0–36)

*ABC*  Aberrant Behavior Checklist, *ASEBA*  Achenbach System of Empirically Based Assessment, *BPC*  Behavior Problem Checklist, *BPI-01*  Behavior Problem Inventory, *CBI*  Challenging Behavior Interview, *DBC*  Developmental Behavior Checklist, *NCBRF*  Nisonger Child Behavior Rating Form, *Reiss*  Reiss Scales for Children's Dual Diagnosis, *SDQ*  Strength and Difficulties Questionnaire, *WellSEQ* Well-being in Special Education Questionnaire

^a^Dekker et al. (2002b)

^b^Dekker et al. (2002c)

All measures except the BPC and CBI were supported by evidence regarding internal consistency, and in general, the internal consistency of the scales across instruments was adequate (i.e., 0.70–0.79) to good/excellent (≥ 0.80) (see Table 1 and Method for more details). Evidence of interrater reliability was found for all measures with the exception of the CBI and Reiss; however, with few exceptions (i.e., BPI-01 and DBC), the evidence indicated inadequate agreement (i.e., < 0.60) in most instances. In terms of consistency over time, although there was no evidence found (i.e., it was not examined/reported) for the CBI, Reiss, and SDQ, all other measures were supported by adequate (i.e., 0.60-0.69) or good/excellent test–retest reliability (i.e., ≥ 0.70) with the exception of the BPC (i.e., inadequate reliability: ≤ 0.60). However, studies examining test–retest reliability were inadequate in terms of small sample sizes (*N* < 100), although there were some exceptions (ASEBA: Dekker et al., 2002a, b, c; Wallander et al., 2006); DBC: (Einfeld & Tonge, 1995)).

Regarding the validity of the measures, little evidence of criterion-related validity and content validity was found. Most of the studies used clinician-rated diagnosis/caseness as a criterion or examined meaningful/hypothesized group differences in subscale scores across diagnostic groups (ABC: (Rojahn & Helsel, 1991); ASEBA: (Douma et al., 2006; Koskentausta et al., 2004); DBC: (Brereton et al., 2006; Clarke et al., 2003; Dekker et al., 2002a, b, c; Einfeld & Tonge, 1995; Hassiotis & Turk, 2012; Koskentausta & Almqvist, 2004); NCBRF: (Norris & Lecavalier, 2011; Reiss & Valenti-Hein, 1994)). Regarding the SDQ, Emerson (2005) reported correspondence between subscale scores and diagnoses from a diagnostic interview (Development and Well-Being Assessment; (Goodman et al., 2000) that had not been validated for persons with ID. We identified more reports of criterion-related validity (i.e., clinician-rated diagnosis/caseness/hypothesized group differences in subscale scores across diagnostic groups) that were reported as good/excellent (i.e., ≥ 0.35) for the DBC compared to the other measures, and no evidence on this aspect for the BPC, BPI-01, CBI, and WellSEQ (Table 4). Evidence of content validity was reported for most of the ID measures (CBI, DBC, NCBRF, Reiss, and WellSEQ) and for one non-ID measure (SDQ).

The majority of measures were supported by evidence of construct validity in terms of correlations between instruments assessing similar constructs, with the exception of the BPC and Reiss, where no evidence was found. Regarding the non-ID instruments, evidence of construct validity was reported for the ASEBA and SDQ, where ID instruments were the most commonly used benchmarks (in alphabetical order: ABC, BPI-01, DBC, NCBRF, and Psychopathology Instrument for Mentally Retarded Adults) (see Table 2). Likewise, evidence of construct validity was reported for the ID instruments, where the other ID measures were most often used as benchmarks (in alphabetical order: ABC, BPI-01, DBC, and NCBRF) (Tables 2 and 4). In relation to sample sizes and reported evidence of construct validity, the NCBRF was examined in the most studies that were large enough (*N* > 200 in four studies), followed by the BPI-01 and DBC (both had *N* > 200 in two studies and *N* = 100–200 in one study), ASEBA (*N* > 200 in two studies), and SDQ (*N* > 200 in one study). Moreover, papers/studies examining the CBI and WellSEQ both reported evidence of construct validity using inadequate sample sizes (*N* < 100).

Exploratory factor analysis (EFA) was used with all measures except the CBI and WellSEQ, and these studies most often used principal component analysis (Table 2). The measure that had the factor structure (FS) examined in the most papers/studies was the ABC (five studies), followed by the DBC (four studies), NCBRF (three studies), BPI-01 (two studies), and ASEBA/BPC/Reiss (all one study each). In regard to the ABC, all studies except one reported adequate to good/excellent FS (Tables 2 and 4). In addition, Sansone et al. (2012) reported an inadequate FS in a syndrome-specific sample (Fragile X) and suggested an alternative FS, with one factor unchanged (inappropriate speech), four modified (irritability, hyperactivity, lethality/withdrawal, and stereotypy), and a new social avoidance factor. Borthwick-Duffy et al. (1997) reported an adequate FS for ASEBA–CBCL only for the broadband internalizing and externalizing factors, although the analysis was based on an inadequate sample size (*N* < 100). An inadequate FS was also reported for the BPC in a large study (Matson et al., 1984). The FS of the BPI-01 using confirmatory FA (CFA) was found to be good/excellent in one large study in a specialized ID institution in France and inadequate in a large special education sample in the US (Table 4). In regard to the DBC, all studies were large; most studies reported an adequate FS, and one reported a good/excellent FS, all by means of EFA (Table 4). Regarding the NCBRF, good/excellent FS was reported in one large study among outpatients using EFA, adequate FS in a large CFA study among special education students/outpatients, and inadequate FS in another large CFA study among special education students (Table 4). The only study that examined the Reiss was large and reported a good/excellent FS (Reiss & Valenti-Hein, 1994). The FS of the SDQ in terms of the broader internalizing and externalizing subscales (alongside the fifth prosocial subscale) was found to be adequate in one large study among students from Saudi Arabia and Oman using CFA (El-Keshky & Emam, 2015) and inadequate in a smaller sample (*N* = 128) of students from Australia examining the original five-factor structure (Table 4).

In relation to self-report, the ASEBA–YSR and WellSEQ were the only measures with evidence of reported adequate aspects of reliability and an adequate aspect of validity (see Table 4). However, the evidence was not confirmed by supporting studies.

Based on the EFPA review model (see Method), all studies examining each individual measurement tool were then included *in the overall assessment of each measure* (Table 5), allowing us to establish the weight of evidence for each measure.

**Table 5:** Summary of overall quality of the psychometric properties of each assessment

Assessment	Reliability			Validity				Overall quality assessment score
	Internal consistency	Test–Retest	Inter-Rater	Criterion	Content	Construct		*M (SD)*
						Factor structure	Convergent	
ABC	GE (4)	GE (1)	A (1) IA (2)	GE (1)	NR	GE (1) A (3) IA (1)	A (1)	11.43 (4.99)
ASEBA	GE (2) A (5)	GE (2)	IA (3)	A (1) IA (1)	NR	A (1)	GE (4) A (2) IA (2)	8.15 (6.32)
BPC	NR	IA (1)	IA (2)	NR	NR	IA (1)	NR	3.33 (1.15)
BPI-01	GE (1) A (3)	GE (1)	GE (1) A (1)	NR	NR	GE (1) IA (1)	GE (2) A (1)	12.50 (5.69)
CBI	NR	NR	NR	NR	GE (1)	NR	A (1)	6 (–)
DBC	A (5)	GE (2)	GE (2) A (1)	GE (4) A (2) IA (1)	GE (1)	GE (1) A (3)	GE (3)	12.67 (9.31)
NCBRF	GE (3) A (1)	GE (1)	A (1) IA (2)	A (1)	GE (1)	GE (1) A (1) IA (1)	GE (3) A(1)	11.25 (8.35)
Reiss	A (1)	NR	NR	IA (1)	GE (1)	GE (1)	NR	16 (–)
SDQ	A (3) IA (3)	NR	IA (1)	GE (1) IA (1)	IA (1)	A (1) IA (1)	A (2)	5.56 (3.24)
WellSEQ	A (1)	A (1)	IA (1)	NR	GE (1)	NR	A (1)	15 (–)

Numbers in parentheses indicate the number of studies that reported on a given psychometric property.

*A*  adequate, *GE*  good–excellent, *IA* inadequate, *NR*  not reported, *ABC*  Aberrant Behavior Checklist, *ASEBA*  Achenbach System of Empirically Based Assessment, *BPC*  Behavior Problem Checklist, *BPI-01*  Behavior Problem Inventory, *CBI*  Challenging Behavior Interview, *DBC*  Developmental Behavior Checklist, *NCBRF*  Nisonger Child Behavior Rating Form, *Reiss*  Reiss Scales for Children's Dual Diagnosis, *SDQ*  Strength and Difficulties Questionnaire, *WellSEQ* Well-being in Special Education Questionnaire

As seen in Table 5, the DBC was the only measure with at least two aspects of reliability (i.e., test–retest and interrater) assessed as good/excellent by two studies, in addition to all validity aspects assessed with evidence of good/excellent with more than one supporting study in relation to criterion and construct validity (convergent validity). The ABC, NCBRF and BPI-01 had two or more aspects of reliability and validity assessed as good/excellent, but at least two aspects of reliability and validity, each in the good/excellent range, were not confirmed by a supporting study. The non-ID measure ASEBA had two aspects of reliability assessed as good/excellent, as reported by two studies, but only convergent validity was reported as good/excellent by supporting studies, and no other validity aspect was assessed in the good/excellent range. The remaining four measures (Reiss, CBI, SDQ, WellSEQ) had no aspects of reliability assessed as good/excellent with supporting studies, although the Reiss had two aspects of validity assessed as good/excellent with no supporting study, and comparably, the CBI/SDQ/WellSEQ had one aspect of validity assessed as good/excellent. The BPC had no aspect of reliability or validity assessed as good/excellent or adequate.

Furthermore, the average psychometric quality, based on the sum score (maximum possible quality score = 35; Table 4) for each measurement tool as they were scored during the quality assessment of the studies, indicated relatively large differences. In general, quality for the ID measures (*M* = 12.03, *SD* = 7.30) was better than for the non-ID measures (*M* = 6.64, *SD* = 5.16) (Table 5). Moreover, the average psychometric quality (Table 5) based on the quality assessment sum score was quite similar among the different ID measures, although the number of studies reporting psychometric properties for each measure greatly varied (e.g., DBC in 10 papers versus WellSEQ in 1 paper). Therefore, when examining, for instance, the ID measures Reiss and WellSEQ with a relatively high average psychometric quality score, it is important to be aware that the documentation was very limited, as shown in the associated standard deviation values in Table 5.

## Discussion

Careful assessment of MH is recommended among all people with ID due to the high vulnerability of this population for developing MH disorders. Our systematic review on the measurement properties of general MH instruments used with children and adolescents with ID identified documentation for ten instruments. The instruments can be divided into two main groups: instruments specifically developed or adapted for the ID population (ID instruments: Aberrant Behavior Checklist [ABC], Behavior Problems Inventory [BPI-01], Challenging Behavior Inventory [CBI], Developmental Behavior Checklist [DBC], Nisonger Child Behavior Rating Form [NCBRF], Reiss Scales for Children’s Dual Diagnosis [Reiss], and Well-Being in Special Education Questionnaire [WellSEQ]) and instruments developed for the general child population (non-ID instruments: Achenbach System of Empirically Based Assessment [ASEBA], Behavior Problem Checklist [BPC educational setting], and Strengths and Difficulties Questionnaire [SDQ]). All identified instruments were screening measures to be used in an initial assessment of MH problems. Of the identified instruments, only the ASEBA had DSM-oriented subscales. The other instruments, including the additional ASEBA subscales, were based on specific descriptors of children’s functioning (e.g., from a literature review, case files, expert consultations, or other existing measures), which were then refined through empirical results and most often from principal component analysis.

The main finding from the present systematic review was consistently better documentation of reliability and validity in terms of higher overall average quality assessment (sum) scores for the ID instruments than for the non-ID instruments. Overall, there were comparable average quality assessment sum scores among the different ID instruments in situations where we identified measures with the most papers reporting psychometric properties (i.e., ABC, BPI-01, DBC, and NCBRF). For the ID instruments CBI, Reiss, and WellSEQ, the findings were more limited due to very little documentation (i.e., one paper each reporting psychometric properties). Regarding the non-ID instruments, the ASEBA gained a higher overall quality score than the other non-ID instruments (i.e., BPC and SDQ). Nevertheless, the average overall quality score for the ASEBA was lower than that for the ID instruments ABC, BPI-01, DBC, and NCBRF.

When examining the overall assessment of each measure in more detail, the DBC was the only measure with most aspects of reliability (test–retest and interrater) and all aspects of validity (criterion, content, factor structure, and convergent validity) assessed as good/excellent, with more than one supporting study for at least two aspects of reliability and validity. The other ID instruments, the ABC, BPI-01, and NCBRF, had several aspects of reliability and validity assessed as good/excellent but fewer supporting studies than the DBC. Regarding the non-ID instruments, the ASEBA had two aspects of reliability (internal consistency and test–retest) assessed as good/excellent by two studies, however, with the exception of convergent validity, other validity aspects were not assessed as good/excellent. There was less evidence for SDQ suitability in terms of reliability and validity compared to ASEBA suitability. Based on the documentation identified for the BPC (i.e., no aspects assessed as good/excellent or adequate), we would not recommend the continued use of this instrument in its current form for this population, and this is probably reflected by the most recent identified study using the BPC being 22 years old (Coe et al., 1999). Regarding documentation of construct validity, in terms of correlations between instruments measuring similar constructs (convergent validity), most of the studies using non-ID measures (e.g., the ASEBA) used ID instruments as benchmarks. Moreover, documentation of construct validity in terms of factor structure was limited for both the non-ID measures ASEBA and SDQ, and these analyses favored the use of the broadband scales (i.e., internalizing and externalizing scales) over the more specific subscales.

It is important to emphasize that the vast majority of studies reporting psychometric properties in the present systematic review involved samples primarily consisting of children and adolescents with a borderline to moderate ID level. This finding is consistent with the findings from a relatively recent systematic review among people of all age groups with severe or profound ID, which found no eligible studies (i.e., at least 70% of the sample within a severe/profound ID level) reporting psychometric properties of measures for children and adolescents (Flynn et al., 2017). Whether the various instruments are suitable for children and adolescents with severe and profound ID is therefore largely unknown and should be investigated in future studies. Furthermore, regarding the ID status of the participants, the majority of the studies in the present systematic review used an administrative operationalization of ID status (e.g., school placement). Accordingly, with few exceptions, a formal IQ assessment or adaptive assessment was not conducted or reported. An implication of this is that the ID concept/condition was loosely defined; therefore, we cannot rule out that the studies included children and adolescents who would not qualify for a formal ID diagnosis.

The perspective of the child or adolescent in terms of self-report measures was very limited, as identified in the present review. The vast majority of studies used informant-based measures completed by parents/caregivers, teachers and (health) care staff. We identified three self-report measures (i.e., the ASEBA, SDQ, and WellSEQ), in which the non-ID measure ASEBA–YSR and the ID measure WellSEQ reported very limited data indicating adequate reliability and validity mainly in samples with mild ID (Bostrom et al., 2016; Douma et al., 2006). However, the evidence was not confirmed by supporting studies. Further development and refinement of the usage of self-reporting, if possible, will be an important development area for the field. The use of multiple informants, including the youths themselves, is recommended, as individuals who have difficulties conveying information on symptoms verbally may display these in varying ways, and no single informant is likely to have a complete overview of another person’s life (Stratis & Lecavalier, 2015). The heterogeneity of ID suggests that a single measure able to identify MH problems across the ID population is unlikely to be constructed in the near future, thereby underscoring the importance of individualized, multimodal and multi-informant approaches to assessment (Halvorsen et al., 2022). MH assessment is recommended by the use of standardized measures where the clinician also considers the strengths and weaknesses of the instrument, which has been the focus of the current systematic review.

Our findings should be interpreted in the context of the strengths and limitations of the study. To our knowledge, this review is the first recent systematic review to examine the psychometric properties of measurement tools used to assess general MH problems in children and adolescents across the whole ID spectrum. We did not limit the review to ID instruments only, as the field (clinic and research community) is characterized by the use of ID and non-ID instruments. We limited the review to studies using mainly children/adolescent samples and did not include findings from studies that used mixed-age samples that also included adults above 25 years of age. We chose to do so because a mixed age range than includes adults can provide findings that are not necessarily transferable to children. Additionally, studies that reported only prevalence rates (or instrument mean scores of MH problems/disorders) in children and adolescents with ID were not eligible because they did not report psychometric properties. We did not evaluate the measure's norms as norming data for the measures was only reported by two of the studies (ABC: Marshburn & Aman, 1992; NCBRF; Tasse et al., 1996), and norms for measures published in manuals through publishers were not included. Another limitation of the study was that we did not calculate inter-rater reliability for the full-text review and data extraction. We did however calculate inter-rater reliability for the quality assessment for 20 randomly chosen studies reporting psychometric properties, and these assessments showed an excellent degree of correspondence (*r* = 0.92) for the sum scores. Finally, the vast majority of the identified measures had one or more studies that reported psychometric properties for the non-English versions. It is important for future studies to establish linguistic equivalence and to determine consistency of the measure’s psychometric properties.

## Conclusion

This systematic review contributes to the field of MH assessment among children and adolescents with ID by examining the psychometric properties of measurement tools used to assess general MH problems in children and adolescents with a borderline to moderate level of ID. Our findings support the use of standardized ID instruments as the first choice in an initial assessment. Very few self-report measures have been developed for children and adolescents with ID, and very few studies have examined their suitability. How to integrate the youths’ perspectives in assessing MH problems is an important focus area in the future.

## Appendix III Supplementary material: Excluded studies with exclusion reasons

Abdelghani, E. A., Apollonsky, N., Bernstein, B., & Tarazi, R. (2017). Steady-state cognitive function and pain severity in youth with sickle cell disease. *Blood. Conference: 59th Annual Meeting of the American Society of Hematology, ASH, 130*(Supplement 1). Exclusion reason: Wrong patient population

Abozeid, M., Hamouda, M., Bahry, H., Elmadny, A., Alakbawy, A., & Ismail, A. (2011). Psychiatric morbidity among a sample of orphanage children in Cairo. *European Child and Adolescent Psychiatry, 1)*, S166-S167. 10.1007/s00787-011-0181-5. Exclusion reason: Conference Abstract

Accordino, R. E., Kidd, C., Politte, L. C., Henry, C. A., & McDougle, C. J. (2016). Psychopharmacological interventions in autism spectrum disorder. *Expert Opinion on Pharmacotherapy, 17*(7), 937-952. 10.1517/14656566.2016.1154536. Exclusion reason: Review

Acharya, A. K. (2016). A study on adolescent mental disorders prevalent in our country. a study. *Indian Journal of Psychiatry, 58 (5 Supplement 1)*, S120. Exclusion reason: Wrong patient population

Achenbach, T. M., & Dumenci, L. (2001). Advances in empirically based assessment: Revised cross-informant syndromes and new DSM-oriented scales for the CBCL, YSR, and TRF: Comment on Lengua, Sadowski, Friedrich, and Fisher (2001). *Journal of Consulting and Clinical Psychology, 69*(4), 699-702. 10.1037/0022-006X.69.4.699. Exclusion reason: Theoretical article/Comment

Achenbach, T. M., Dumenci, L., & Rescorla, L. A. (2003). DSM-Oriented and Empirically Based Approaches to Constructing Scales From the Same Item Pools. *Journal of Clinical Child and Adolescent Psychology, 32*(3), 328-340. 10.1207/S15374424JCCP3203_02. Exclusion reason: Wrong patient population

Achtergarde, S., Becke, J., Beyer, T., Postert, C., Romer, G., & Muller, J. M. (2014). Preschool-age male psychiatric patients with specific developmental disorders and those without: Do they differ in behavior problems and treatment outcome? *Infants & Young Children, 27*(4), 359-377. 10.1097/IYC.0000000000000020. Exclusion reason: Wrong patient population

Adamo, N., Michelini, G., Cheung, C. H. M., Buitelaar, J. K., Asherson, P., Rijsdijk, F., & Kuntsi, J. (2019). Does Co-Occurring Anxiety Modulate ADHD-Related Cognitive and Neurophysiological Impairments? *Journal of Attention Disorders*, 1087054719879499. 10.1177/1087054719879499. Exclusion reason: Wrong patient population

Adams, D., & Allen, D. (2001). Assessing the need for reactive behaviour management strategies in children with intellectual disability and severe challenging behaviour. *Journal of Intellectual Disability Research, 45*(4), 335-343. Exclusion reason: No psychometric information

Adams, D., Handley, L., Simkiss, D., Walls, E., Jones, A., Knapp, M., . . . Oliver, C. (2018). Service Use and Access in Young Children with an Intellectual Disability or Global Developmental Delay: Associations with Challenging Behaviour. *Journal of Intellectual & Developmental Disability, 43*(2), 232-241. Exclusion reason: No psychometric information

Adams, D., Paynter, J., Clark, M., Roberts, J., & Keen, D. (2019). The Developmental Behaviour Checklist (DBC) profile in young children on the autism spectrum: The impact of child and family factors. *Journal of Autism and Developmental Disorders, 49*(8), 3426-3439. 10.1007/s10803-019-04067-0. Exclusion reason: Wrong patient population

Adams, H., de Blieck, E. A., Mink, J. W., Marshall, F. J., Kwon, J., Dure, L., . . . Pearce, D. A. (2006). Standardized assessment of behavior and adaptive living skills in juvenile neuronal ceroid lipofuscinosis. *Developmental Medicine & Child Neurology, 48*(4), 259-264. 10.1017/S0012162206000570. Exclusion reason: Not relevant measurement tool

Adams, P. N. (1998). Utilizing behavioral diagnostics to reduce disruptive behavior in public school settings with children and adolescents with severe emotional and behavioral disorders. *Dissertation Abstracts International: Section B: The Sciences and Engineering, 58*(7-B), 3913. Exclusion reason: Not relevant outcome

Adeosun, I. I., Ogun, O. C., Ijarogbe, T., Bello, A. O., Adegbohun, A., & Omigbodun, O. O. (2012). Self-injurious behaviour in Nigerian children with intellectual disability. *Neuropsychiatrie de l'Enfance et de l'Adolescence, 1)*, S170. 10.1016/j.neurenf.2012.04.253. Exclusion reason: Conference Abstract

Adrien, J.-L., Roux, S., Couturier, G., Malvy, J., Guerin, P., Debuly, S., . . . Barthelemy, C. (2001). Towards a new functional assessment of autistic dysfunction in children with developmental disorders: The Behaviour Function Inventory. *Autism, 5*(3), 249-264. 10.1177/1362361301005003003. Exclusion reason: Wrong patient population

Advokat, C. D., Mayville, E. A., & Matson, J. L. (2000). Side effect profiles of atypical antipsychotics, typical antipsychotics, or no psychotropic medications in persons with mental retardation. *Research in Developmental Disabilities, 21*(1), 75-84. 10.1016/s0891-4222(99)00031-1. Exclusion reason: Wrong patient population

Agarwal, V., Sitholey, P., Kumar, S., & Prasad, M. (2001). Double-blind, placebo-controlled trial of clonidine in hyperactive children with mental retardation. *Mental Retardation, 39*(4), 259-267. 10.1352/0047-6765%282001%29039%3C0259:DBPCTO%3E2.0.CO;2. Exclusion reason: Outcome: psychopharmacology

Ahuja, A., Martin, J., Langley, K., & Thapar, A. (2013). Intellectual disability in children with attention deficit hyperactivity disorder. *Journal of Pediatrics, 163*(3), 890-895.e891. 10.1016/j.jpeds.2013.02.043. Exclusion reason: No psychometric information

Aishworiya, R., Chan, P. F., Kiing, J. S. H., Chong, S. C., & Tay, S. K. H. (2016). Sleep Patterns and Dysfunctions in Children with Learning Problems. *Annals Academy of Medicine Singapore, 45*(11), 507-512. Exclusion reason: Not relevant measurement tool

Aito, C., Mizoguchi, Y., Yamamoto, M., SeguchI, Y., Yatsuga, C., Nishimura, T., . . . Monji, A. (2019). Oxytocin levels and sex differences in autism spectrum disorder with severe intellectual disabilities. *Psychiatry Research, 273*, 67-74. 10.1016/j.psychres.2018.12.139. Exclusion reason: Results not reported separately for children and adolescents

Akefeldt, A., Gillberg, C., & Larsson, C. (1991). Prader-Willi syndrome in a Swedish rural county: Epidemiological aspects. *Developmental Medicine & Child Neurology, 33*(8), 715-721. 10.1111/j.1469-8749.1991.tb14950.x. Exclusion reason: No psychometric information

Aktepe, E., & Sonmez, Y. (2012). Psychiatric and organic comorbidities in children diagnosed with mental retardation in a university hospital. *Yeni Symposium: psikiyatri, noroloji ve davranis bilimleri dergisi, 50*(2), 67-75. Exclusion reason: Conference Abstract

Alaimo, J. T., Barton, L. V., Mullegama, S. V., Wills, R. D., Foster, R. H., & Elsea, S. H. (2015). Individuals with Smith-Magenis syndrome display profound neurodevelopmental behavioral deficiencies and exhibit food-related behaviors equivalent to Prader-Willi syndrome. *Research in Developmental Disabilities, 47*, 27-38. 10.1016/j.ridd.2015.08.011. Exclusion reason: Results not reported separately for children and adolescents

Alexander, R. T., Crouch, K., Halstead, S., & Piachaud, J. (2006). Long-term outcome from a medium secure service for people with intellectual disability. *Journal of Intellectual Disability Research, 50*(4), 305-315. 10.1111/j.1365-2788.2006.00806.x. Exclusion reason: Results not reported separately for children and adolescents

Alfieri, P., Demaria, F., Licchelli, S., Santonastaso, O., Caciolo, C., Digilio, M. C., . . . Vicari, S. (2019). Obsessive Compulsive Symptoms and Psychopathological Profile in Children and Adolescents with KBG Syndrome. *Brain Sciences, 9*(11). 10.3390/brainsci9110313. Exclusion reason: No psychometric information

Algozzine, B. (2012). *Disturbing Behavior Checklists" Technical Manual*. Retrieved from http://search.ebscohost.com/login.aspx?direct=true&db=eric&AN=ED529898&site=ehost-live

Algozzine, B., Ysseldyke, J., & Minnesota Univ, M. I. f. R. o. L. D. (1982). *Learning Disabilities as a Subset of School Failure: The Oversophistication of a Concept*. Retrieved from http://search.ebscohost.com/login.aspx?direct=true&db=eric&AN=ED218852&site=ehost-live

Algozzine, B., & Ysseldyke, J. E. (1983). Learning disabilities as a subset of school failure: The oversophistication of a concept. *Exceptional Children, 50*(3), 242-246. Exclusion reason: No psychometric information

Allen, D., Lowe, K., Brophy, S., & Moore, K. (2009). Predictors of restrictive reactive strategy use in people with challenging behaviour. *Journal of Applied Research in Intellectual Disabilities, 22*(2), 159-168. 10.1111/j.1468-3148.2008.00484.x. Exclusion reason: Results not reported separately for children and adolescents

Allen, D., Lowe, K., Moore, K., & Brophy, S. (2007). Predictors, costs and characteristics of out of area placement for people with intellectual disability and challenging behaviour. *Journal of Intellectual Disability Research, 51*(6), 409-416. 10.1111/j.1365-2788.2006.00877.x. Exclusion reason: Results not reported separately for children and adolescents

Allgaier, A. K., Pietsch, K., Fruhe, B., Sigl-Glockner, J., & Schulte-Korne, G. (2012). SCREENING FOR DEPRESSION IN ADOLESCENTS: VALIDITY OF THE PATIENT HEALTH QUESTIONNAIRE IN PEDIATRIC CARE. *Depression and Anxiety, 29*(10), 906-913. 10.1002/da.21971. Exclusion reason: Wrong patient population

Allgaier, A.-K., Fruhe, B., Pietsch, K., Saravo, B., Baethmann, M., & Schulte-Korne, G. (2012). Is the Children's Depression Inventory Short version a valid screening tool in pediatric care? A comparison to its full-length version. *Journal of Psychosomatic Research, 73*(5), 369-374. 10.1016/j.jpsychores.2012.08.016. Exclusion reason: Wrong patient population

Alrojolah, L., Beayno, A., Shamseddeen, W., Ghandour, L., Akoury Dirani, L., & Maalouf, F. (2019). 4.67 Chronic Physical Illness and Psychiatric Comorbidities in Lebanese Adolescents. *Journal of the American Academy of Child and Adolescent Psychiatry, 58 (10 Supplement)*, S242. 10.1016/j.jaac.2019.08.307. Exclusion reason: Conference Abstract

Altepeter, T. S., & Breen, M. J. (1989). The Home Situations Questionnaire (HSQ) and the School Situations Questionnaire (SSQ): Normative data and an evaluation of psychometric properties. *Journal of Psychoeducational Assessment, 7*(4), 312-322. 10.1177/073428298900700404. Exclusion reason: Wrong patient population

Althaus, M., Minderaa, R. B., & Dienske, H. (1994). The assessment of individual differences between young children with a pervasive developmental disorder by means of behaviour scales which are derived from direct observation. *Journal of Child Psychology & Psychiatry & Allied Disciplines, 35*(2), 333-349. Exclusion reason: Not relevant measurement tool

Alvarez, Z. C. (2016). Gender equivalence as perceived by students, parents, and teachers on the behavior assessment system for children, second edition. *Dissertation Abstracts International: Section B: The Sciences and Engineering, 76*(11-B(E)), No Pagination Specified. Exclusion reason: No intellectual disability information

Alves, D., Sousa, M., Henriques, M., & De Lemos, M. S. (2013). A triadic model for learning disabilities. *Atencion Primaria, 2)*, 48-49. 10.1016/S0212-6567%2813%2970032-5. Exclusion reason: Conference Abstract

Al-Yagon, M. (2012). Subtypes of attachment security in school-age children with learning disabilities. *Learning Disability Quarterly, 35*(3), 170-183. Exclusion reason: Not relevant measurement tool

Al-Yagon, M. (2013). Adolescents with LD: Socioemotional and behavioral functioning and attachment relationships with fathers, mothers, and teachers. *European Child and Adolescent Psychiatry, 1)*, S220. 10.1007/s00787-013-0423-9. Exclusion reason: Conference Abstract

Al-Yagon, M. (2016). Perceived Close Relationships with Parents, Teachers, and Peers: Predictors of Social, Emotional, and Behavioral Features in Adolescents With LD or Comorbid LD and ADHD. *Journal of Learning Disabilities, 49*(6), 597-615. Exclusion reason: Not relevant measurement tool

Amador, J. A., Forns, M., & Martorell, B. (2001). Sensitivity and specificity of parents' and teachers' ratings of Attention Deficit Hyperactivity Disorder. *Anuario de Psicologia, 32*(4), 65-78. Exclusion reason: Not relevant measurement tool

Aman, M., Buitelaar, J., De Smedt, G., Wapenaar, R., & Binder, C. (2005). Pharmacotherapy of Disruptive Behavior and Item Changes on a Standardized Rating Scale: Pooled Analysis of Risperidone Effects in Children with Subaverage IQ. *Journal of Child and Adolescent Psychopharmacology, 15*(2), 220-232. 10.1089/cap.2005.15.220. Exclusion reason: Outcome: psychopharmacology

Aman, M., Leone, S., Lecavalier, L., Park, L., Buican, B., & Coury, D. (2008). The Nisonger Child Behavior Rating Form: Typical IQ version. *International Clinical Psychopharmacology, 23*(4), 232-242. 10.1097/YIC.0b013e3282f94ad0. Exclusion reason: Wrong patient population

Aman, M. G. (1991). Review and evaluation of instruments for assessing emotional and behavioural disorders. *Australia and New Zealand Journal of Developmental Disabilities, 17*(2), 127-145. Exclusion reason: Review

Aman, M. G., Buican, B., & Arnold, L. (2003). Methylphenidate treatment in children with borderline IQ and mental retardation: Analysis of three aggregated studies. *Journal of Child and Adolescent Psychopharmacology, 13*(1), 29-40. 10.1089/104454603321666171. Exclusion reason: No psychometric information

Aman, M. G., Burrow, W. H., & Wolford, P. L. (1995). The Aberrant Behavior Checklist-Community: factor validity and effect of subject variables for adults in group homes. *American Journal of Mental Retardation, 100*(3), 283-292. Exclusion reason: Adult population

Aman, M. G., De Smedt, G., Derivan, A., Lyons, B., & Findling, R. L. (2002). Double-blind, placebo-controlled study of risperidone for the treatment of disruptive behaviors in children with subaverage intelligence. *The American Journal of Psychiatry, 159*(8), 1337-1346. 10.1176/appi.ajp.159.8.1337. Exclusion reason: Outcome: psychopharmacology

Aman, M. G., & Gharabawi, G. M. (2004). Treatment of Behavior Disorders in Mental Retardation: Report on Transitioning to Atypical Antipsychotics, With an Emphasis on Risperidone. *The Journal of Clinical Psychiatry, 65*(9), 1197-1210. 10.4088/JCP.v65n0907. Exclusion reason: Review

Aman, M. G., Kern, R. A., McGhee, D. E., & Arnold, L. (1993). Fenfluramine and methylphenidate in children with mental retardation and ADHD: Clinical and side effects. *Journal of the American Academy of Child & Adolescent Psychiatry, 32*(4), 851-859. 10.1097/00004583-199307000-00022. Exclusion reason: Outcome: psychopharmacology

Aman, M. G., Kern, R. A., McGhee, D. E., & Arnold, L. (1993). Fenfluramine and methylphenidate in children with mental retardation and attention deficit hyperactivity disorder: Laboratory effects. *Journal of Autism and Developmental Disorders, 23*(3), 491-506. 10.1007/BF01046052. Exclusion reason: No psychometric information

Aman, M. G., Marks, R. E., Turbott, S. H., Wilsher, C. P., & Merry, S. N. (1991). Clinical effects of methylphenidate and thioridazine in intellectually subaverage children. *Journal of the American Academy of Child & Adolescent Psychiatry, 30*(2), 246-256. 10.1097/00004583-199103000-00013. Exclusion reason: Outcome: psychopharmacology

Aman, M. G., Norris, M., Kaat, A. J., Andrews, H., Choo, T. H., Chen, C., . . . Erickson, C. (2020). Factor structure of the aberrant behavior checklist in individuals with fragile x syndrome: Clarifications and future guidance. *Journal of Child and Adolescent Psychopharmacology, 30*(8), 512-521. 10.1089/cap.2019.0177. Exclusion reason: Results not reported separately for children and adolescents

Aman, M. G., & Rojahn, J. (1994). THE PSYCHOMETRIC CHARACTERISTICS OF THE PRESCHOOL BEHAVIOR QUESTIONNAIRE IN PRESCHOOLERS WITH DEVELOPMENTAL HANDICAPS. *Journal of Developmental and Physical Disabilities, 6*(4), 311-325. 10.1007/bf02578418. Exclusion reason: Not relevant measurement tool

Aman, M. G., Singh, N. N., Stewart, A. W., & Field, C. J. (1985). The aberrant behavior checklist: a behavior rating scale for the assessment of treatment effects. *American Journal of Mental Deficiency, 89*(5), 485-491. Exclusion reason: Results not reported separately for children and adolescents

Aman, M. G., Watson, J. E., Singh, N. N., Turbott, S. H., & Wilsher, C. P. (1986). Psychometric and demographic characteristics of the psychopathology instrument for mentally retarded adults. *Psychopharmacology Bulletin, 22*(4), 1072-1076. Exclusion reason: Results not reported separately for children and adolescents

Ambrosini, P. J. (2000). Historical development and present status of the Schedule for Affective Disorders and Schizophrenia for School-Age Children (K-SADS). *Journal of the American Academy of Child & Adolescent Psychiatry, 39*(1), 49-58. 10.1097/00004583-200001000-00016. Exclusion reason: Review

Amerikaner, M., & Summerlin, M. L. (1982). Group counseling with learning disabled children: Effects of social skills and relaxation training on self-concept and classroom behavior. *Journal of Learning Disabilities, 15*(6), 340-343. 10.1177/002221948201500607. Exclusion reason: No psychometric information

Amon, P., Beck, B., Castell, R., Teicher, C., & et al. (1995). The course of psychiatric disorders and specific developmental disorders in children with learning disabilities. *Vol 23(3), 1995, pp 171-181, 23*(3), 171-181. Exclusion reason: No psychometric information

An, X., Rojahn, J., Curby, T. W., & Ding, Y. (2015). Psychometric properties of the Chinese Behavior Problems Inventory-01 in children and adolescents with or at risk for intellectual disabilities. *Research in Developmental Disabilities, 36*, 256-263. 10.1016/j.ridd.2014.10.006. Exclusion reason: Not relevant measurement tool

Anderson, C. M., Freeman, K. A., & Scotti, J. R. (1999). Evaluation of the generalizability (reliability and validity) of analog functional assessment methodology. *Behavior Therapy, 30*(1), 31-50. 10.1016/S0005-7894%2899%2980044-6. Exclusion reason: Not relevant measurement tool

Anderson, P., Doyle, L. W., & Victorian Infant Collaborative Study, G. (2003). Neurobehavioral outcomes of school-age children born extremely low birth weight or very preterm in the 1990s. *JAMA, 289*(24), 3264-3272. Exclusion reason: No intellectual disability information

Anderson, V. A., Anderson, P., Northam, E., Jacobs, R., & Mikiewicz, O. (2002). Relationships between cognitive and behavioral measures of executive function in children with brain disease. *Child Neuropsychology, 8*(4), 231-240. Exclusion reason: Not relevant measurement tool

Andrei, L. E., Cerlinca, A. I., Neacsu, R. D., Niculae, A. L., & Mihailescu, I. (2019). The course of ADHD diagnosis over a 6-year timeframe in a Romanian inpatient sample. *European Neuropsychopharmacology, 29 (Supplement 1)*, S434-S435. 10.1016/j.euroneuro.2018.11.655. Exclusion reason: Conference abstract

Andrei, L. E., Neacsu, R. D., Irimie-Ana, A., Dobrescu, I., & Rad, F. (2019). P.862 Oppositional defiant disorder - conduct disorder - antisocial personality disorder continuum investigated over a 14-year timespan. *European Neuropsychopharmacology, 29 (Supplement 6)*, S574-S575. 10.1016/j.euroneuro.2019.09.724. Exclusion reason: Conference abstract

Aneja, A., Fremont, W. P., Antshel, K. M., Faraone, S. V., AbdulSabur, N., Higgins, A. M., . . . Kates, W. R. (2007). Manic symptoms and behavioral dysregulation in youth with velocardiofacial syndrome (22q11.2 Deletion Syndrome). *Journal of Child and Adolescent Psychopharmacology, 17*(1), 105-114. 10.1089/cap.2006.0023. Exclusion reason: No psychometric information

Anonymous. (1998). Practice parameters for the assessment and treatment of children and adolescents with language and learning disorders. AACAP. *Journal of the American Academy of Child & Adolescent Psychiatry, 37*(10 Suppl), 46S-62S. Exclusion reason: Theoretical article/Comment

Anonymous. (2006). Mental health in the United States: parental report of diagnosed autism in children aged 4-17 years--United States, 2003-2004. *Mmwr, Morbidity and mortality weekly report. 55*(17), 481-486. Exclusion reasonhttps://www.cdc.gov/mmwr/preview/mmwrhtml/mm5517a3.htm; Exclusion reason:: No intellectual disability information.

Antonelli, C. J. (1983). Guidelines for Working with Clients/Students Who Engage in Disruptive-Aggressive Behavior and/or Have Adaptive Behavior Deficits. In Applegate, H., Matson, J. L., & Cherry, K. E. (1999). An evaluation of functional variables affecting severe problem behaviors in adults with mental retardation by using the Questions about Behavioral Function Scale (QABF). *Research in Developmental Disabilities, 20*(3), 229-237. 10.1016/S0891-4222%2899%2900005-0. Exclusion reason: Adult population

Appleton, H., Roberts, J., & Simpson, K. (2019). How is Anxiety Identified and Diagnosed in Individuals with Autism Spectrum Disorder and Intellectual Disability? A Scoping Review. *Journal of Mental Health Research in Intellectual Disabilities, 12*(3-4), 152-175. 10.1080/19315864.2019.1679299. Exclusion reason: Review

Aragon, A. S., Coriale, G., Fiorentino, D., Kalberg, W. O., Buckley, D., Gossage, J., . . . May, P. A. (2008). Neuropsychological characteristics of Italian children with fetal alcohol spectrum disorders. *Alcoholism: Clinical and Experimental Research, 32*(11), 1909-1919. Exclusion reason: Wrong patient population

Aram, D. M., Ekelman, B. L., & Nation, J. E. (1984). Preschoolers with language disorders: 10 years later. *Journal of Speech & Hearing Research, 27*(2), 232-244. 10.1044/jshr.2702.244. Exclusion reason: Wrong patient population

Arim, R. G., Kohen, D. E., Garner, R. E., Lach, L. M., Brehaut, J. C., MacKenzie, M. J., & Rosenbaum, P. L. (2015). Psychosocial functioning in children with neurodevelopmental disorders and externalizing behavior problems. *Disability & Rehabilitation, 37*(4), 345-354. 10.3109/09638288.2014.919361. Exclusion reason: No intellectual disability information

Arndorfer, R. E., Miltenberger, R. G., Woster, S. H., Rortvedt, A. K., & Gaffaney, T. (1994). Home-based descriptive and experimental analysis of problem behaviors in children. *Topics in Early Childhood Special Education, 14*(1), 64-87. 10.1177/027112149401400108. Exclusion reason: No intellectual disability information

Arnold, R., Yule, W., & Martin, N. (1985). The psychological characteristics of infantile hypercalcaemia: A preliminary investigation. *Developmental Medicine & Child Neurology, 27*(1), 49-59. 10.1111/j.1469-8749.1985.tb04524.x. Exclusion reason: No psychometric information

Aro, T., Eklund, K., Eloranta, A.-K., Närhi, V., Korhonen, E., & Ahonen, T. (2019). Associations Between Childhood Learning Disabilities and Adult-Age Mental Health Problems, Lack of Education, and Unemployment. *Journal of Learning Disabilities, 52*(1), 71-83. 10.1177/0022219418775118. Exclusion reason: Wrong patient population

Arora, S., Goodall, S., Viney, R., Einfeld, S., & Mhypedd, t. (2020). Health-related quality of life amongst primary caregivers of children with intellectual disability. *Journal of Intellectual Disability Research, 64*(2), 103-116. 10.1111/jir.12701. Exclusion reason: No psychometric information

Asadabadi, M., Mohammadi, M.-R., Ghanizadeh, A., Modabbernia, A., Ashrafi, M., Hassanzadeh, E., . . . Akhondzadeh, S. (2013). Celecoxib as adjunctive treatment to risperidone in children with autistic disorder: A randomized, double-blind, placebo-controlled trial. *Psychopharmacology, 225*(1), 51-59. 10.1007/s00213-012-2796-8. Exclusion reason: Outcome: psychopharmacology

Aunos, M., Feldman, M., & Goupil, G. (2008). Mothering with intellectual disabilities: Relationship between social support, health and well-being, parenting and child behaviour outcomes. *Journal of Applied Research in Intellectual Disabilities, 21*(4), 320-330. 10.1111/j.1468-3148.2008.00447.x. Exclusion reason: No psychometric information

Avrahamy, H., Pollak, Y., Shriki-Tal, L., Genstil, L., Hirsch, H. J., Gross-Tsur, V., & Benarroch, F. (2015). A disease specific questionnaire for assessing behavior in individuals with prader-willi syndrome. *Comprehensive Psychiatry, 58*, 189-197. 10.1016/j.comppsych.2014.12.005. Exclusion reason: Results not reported separately for children and adolescents

Babcock, S. E., Miller, J. L., Saklofske, D. H., & Zhu, J. (2018). WISC-V Canadian norms: Relevance and use in the assessment of Canadian children. *Canadian Journal of Behavioural Science / Revue canadienne des sciences du comportement, 50*(2), 97-104. 10.1037/cbs0000096. Exclusion reason: Not relevant measurement tool

Backes, M., Genc, B., Schreck, J., Doerfler, W., Lehmkuhl, G., & von Gontard, A. (2000). Cognitive and behavioral profile of fragile X boys: correlations to molecular data. *American Journal of Medical Genetics, 95*(2), 150-156. Exclusion reason: No psychometric information

Baerga, P. P., Pastrana, M. V., & Bauermeister, J. J. (2017). Assessment of executive functioning in children and adolescents: Validation of the Spanish language Barkley deficits in executive functioning scale for children and adolescents (BDEFS-CA). *ADHD Attention Deficit and Hyperactivity Disorders, 9 (1 Supplement)*, S15-S16. 10.1007/s12402-017-0224-y. Exclusion reason: Not relevant measurement tool

Baeza-Velasco, C., Michelon, C., Rattaz, C., & Baghdadli, A. (2014). Are aberrant behavioral patterns associated with the adaptive behavior trajectories of teenagers with Autism Spectrum Disorders? *Research in Autism Spectrum Disorders, 8*(3), 304-311. 10.1016/j.rasd.2013.12.004. Exclusion reason: No psychometric information

Bailey, D. B., Jr., Hatton, D. D., Mesibov, G., Ament, N., & Skinner, M. (2000). Early development, temperament, and functional impairment in autism and fragile X syndrome. *Journal of Autism & Developmental Disorders, 30*(1), 49-59. Exclusion reason: Not relevant measurement tool

Bailey, D. B., Jr., Raspa, M., Bishop, E., Mitra, D., Martin, S., Wheeler, A., & Sacco, P. (2012). Health and economic consequences of fragile X syndrome for caregivers. *Journal of Developmental and Behavioral Pediatrics, 33*(9), 705-712. 10.1097/DBP.0b013e318272dcbc. Exclusion reason: Results not reported separately for children and adolescents

Bailey, K. M., & Blair, K.-S. C. (2015). Feasibility and potential efficacy of the family-centered Prevent-Teach-Reinforce model with families of children with developmental disorders. *Research in Developmental Disabilities, 47*, 218-233. 10.1016/j.ridd.2015.09.019. Exclusion reason: Not relevant measurement tool

Baio, J., Wiggins, L., Christensen, D. L., Maenner, M. J., Daniels, J., Warren, Z., . . . Dowling, N. F. (2018). Prevalence of Autism Spectrum Disorder Among Children Aged 8 Years - Autism and Developmental Disabilities Monitoring Network, 11 Sites, United States, 2014. *Morbidity & Mortality Weekly Report. Surveillance Summaries, 67*(6), 1-23. 10.15585/mmwr.ss6706a1. Exclusion reason: Not relevant measurement tool

Baker, B. L., & Blacher, J. (2015). Disruptive Behavior Disorders in Adolescents With ASD: Comparisons to Youth With Intellectual Disability or Typical Cognitive Development. *Journal of Mental Health Research in Intellectual Disabilities, 8*(2), 98-116. 10.1080/19315864.2015.1018395. Exclusion reason: Wrong patient population

Baker, B. L., Blacher, J., Crnic, K. A., & Edelbrock, C. (2002). Behavior problems and parenting stress in families of three-year-old children with and without developmental delays. *American Journal on Mental Retardation, 107*(6), 433-444. 10.1352/0895-8017(2002)107<0433:BPAPSI>2.0.CO;2. Exclusion reason: Mean age < 4 years

Baker, B. L., Blacher, J., & Olsson, M. B. (2005). Preschool children with and without developmental delay: behaviour problems, parents' optimism and well-being. *Journal of Intellectual Disability Research, 49*(8), 575-590. Exclusion reason: Wrong patient population

Baker, B. L., McIntyre, L., Blacher, J., Crnic, K., Edelbrock, C., & Low, C. (2003). Pre-school children with and without developmental delay: Behaviour problems and parenting stress over time. *Journal of Intellectual Disability Research, 47*(4-5), 217-230. 10.1046/j.1365-2788.2003.00484.x. Exclusion reason: No intellectual disability information

Baker, D. B., & McCal, K. (1995). Parenting stress in parents of children with attention-deficit hyperactivity disorder and parents of children with learning disabilities. *Journal of Child and Family Studies, 4*(1), 57-68. 10.1007/BF02233954. Exclusion reason: Wrong patient population

Baker, E. K., Godler, D. E., Bui, M., Hickerton, C., Rogers, C., Field, M., . . . Bretherton, L. (2018). Exploring autism symptoms in an Australian cohort of patients with Prader-Willi and Angelman syndromes. *Journal of Neurodevelopmental Disorders, 10*(1), 24. 10.1186/s11689-018-9242-0. Exclusion reason: Not relevant measurement tool

Baker, J. K., Fenning, R. M., Crnic, K. A., Baker, B. L., & Blacher, J. (2007). Prediction of social skills in 6-year-old children with and without developmental delays: Contributions of early regulation and maternal scaffolding. *American Journal on Mental Retardation, 112*(5), 375-391. 10.1352/0895-8017%282007%29112%5B0375:POSSIY%5D2.0.CO;2. Exclusion reason: Not relevant measurement tool

Baker, J. K., Seltzer, M. M., & Greenberg, J. S. (2011). Longitudinal effects of adaptability on behavior problems and maternal depression in families of adolescents with autism. *Journal of Family Psychology, 25*(4), 601-609. 10.1037/a0024409. Exclusion reason: No intellectual disability information

Baker, J. K., Seltzer, M. M., & Greenberg, J. S. (2012). Behaviour problems, maternal internalising symptoms and family relations in families of adolescents and adults with fragile X syndrome. *Journal of Intellectual Disability Research, 56*(10), 984-995. 10.1111/j.1365-2788.2012.01580.x. Exclusion reason: Results not reported separately for children and adolescents

Baker, K., Scerif, G., Astle, D. E., Fletcher, P. C., & Raymond, F. L. (2015). Psychopathology and cognitive performance in individuals with membrane-associated guanylate kinase mutations: A functional network phenotyping study. *Journal of Neurodevelopmental Disorders, 7 (1) (no pagination)*(8). 10.1186/s11689-015-9105-x. Exclusion reason: No psychometric information

Bakhireva, L. N. (2011). Difficulties diagnosing fetal alcohol spectrum disorders and confirming maternal and fetal alcohol exposure: An epidemiologist's perspective. *Birth Defects Research Part A - Clinical and Molecular Teratology, 91 (5)*, 307. 10.1002/bdra.20834. Exclusion reason: Conference abstract

Bakken, T. L., Helverschou, S. B., Eilertsen, D. E., Heggelund, T., Myrbakk, E., & Martinsen, H. (2010). Psychiatric disorders in adolescents and adults with autism and intellectual disability: A representative study in one county in Norway. *Research in Developmental Disabilities, 31*(6), 1669-1677. 10.1016/j.ridd.2010.04.009. Exclusion reason: Results not reported separately for children and adolescents

Balboni, G., Battagliese, G., & Pedrabissi, L. (2000). The Psychopathology Inventory for Mentally Retarded Adults: Factor structure and comparisons between subjects with or without dual diagnosis. *Research in Developmental Disabilities, 21*(4), 311-321. 10.1016/S0891-4222%2800%2900044-5. Exclusion reason: Results not reported separately for children and adolescents

Balboni, G., Pedrabissi, L., Molteni, M., & Villa, S. (2001). Discriminant validity of the Vineland Scales: score profiles of individuals with mental retardation and a specific disorder. *American Journal of Mental Retardation, 106*(2), 162-172. Exclusion reason: Not relevant measurement tool

Balboni, G., Rebecchini, G., Elisei, S., & Tasse, M. J. (2020). Factors affecting the relationship between adaptive behavior and challenging behaviors in individuals with intellectual disability and co-occurring disorders. *Research in Developmental Disabilities Vol 104 2020, ArtID 103718, 104*. 10.1016/j.ridd.2020.103718. Exclusion reason: Wrong patient population

Balboni, G., Tasse, M. J., Schalock, R. L., Borthwick-Duffy, S. A., Spreat, S., Thissen, D., . . . Navas, P. (2014). The diagnostic adaptive behavior scale: evaluating its diagnostic sensitivity and specificity. *Research in Developmental Disabilities, 35*(11), 2884-2893. 10.1016/j.ridd.2014.07.032. Exclusion reason: Not relevant measurement tool

Baleja-Stawicka, I., Pawelczyk, T., Barasinska-Tarka, E., & Rabe-Jablonska, J. (2009). Relation between the mental health condition and the quality of life of a mother and the mental health condition of a child with mental retardation. [Polish, English]. *Psychiatria i Psychologia Kliniczna, 9*(3), 167-177. Exclusion reason: No psychometric information

Ballinger, B. R., Ballinger, C. B., Reid, A. H., & McQueen, E. (1991). The psychiatric symptoms, diagnoses and care needs of 100 mentally handicapped patients. *British Journal of Psychiatry, 158*, 251-254. Exclusion reason: Wrong patient population

Balthazar, E. E., et al., Central Wisconsin, C., & Training School, M. W. I. (1973). Absence of Intervention Training Programs: Effects Upon the Severely and Profoundly Retarded, Part I: Selected Cases of Emotional and Behavioral Disturbances. In. Bao, L., Brownlie, E., & Beitchman, J. H. (2016). Mental health trajectories from adolescence to adulthood: Language disorder and other childhood and adolescent risk factors. *Development and Psychopathology, 28*(2), 489-504. 10.1017/S0954579415001054. Exclusion reason: Wrong patient population

Baranek, G. T., Foster, L. G., & Berkson, G. (1997). Tactile defensivenss and stereotyped behaviors. *American Journal of Occupational Therapy, 51*(2), 91-95. 10.5014/ajot.51.2.91. Exclusion reason: Not relevant measurement tool

Barbarin, O. A. (2007). Mental Health Screening of Preschool Children: Validity and Reliability of ABLE. *American Journal of Orthopsychiatry, 77*(3), 402-418. 10.1037/0002-9432.77.3.402. Exclusion reason: No intellectual disability information

Barkley, R. A., Anastopoulos, A. D., Guevremont, D. C., & Fletcher, K. E. (1991). Adolescents with ADHD: Patterns of behavioral adjustment, academic functioning, and treatment utilization. *Journal of the American Academy of Child & Adolescent Psychiatry, 30*(5), 752-761. 10.1097/00004583-199109000-00009. Exclusion reason: Wrong patient population

Barnard-Brak, L., Rojahn, J., Richman, D. M., Chesnut, S. R., & Wei, T. (2015). Stereotyped behaviors predicting self-injurious behavior in individuals with intellectual disabilities. *Research in Developmental Disabilities, 36*, 419-427. 10.1016/j.ridd.2014.08.017. Exclusion reason: No psychometric information

Barnard-Brak, L., Rojahn, J., & Wei, T. (2013). Psychometric analysis of the behavior problems inventory using an item-response theory framework: A sample of individuals with intellectual disabilities. *Journal of Psychopathology and Behavioral Assessment, 35*(4), 564-577. 10.1007/s10862-013-9356-3. Exclusion reason: Results not reported separately for children and adolescents

Barnes, K. V., Coughlin, F. R., O'Leary, H. M., Bruck, N., Bazin, G. A., Beinecke, E. B., . . . Kaufmann, W. E. (2015). Anxiety-like behavior in Rett syndrome: Characteristics and assessment by anxiety scales. *Journal of Neurodevelopmental Disorders Vol 7(1), 2015, ArtID 30, 7*(1). Exclusion reason: Not relevant measurement tool

Barrera, F. J., & Graver, E. E. (2009). A comparison of behaviour functions in community and facility settings. *Journal on Developmental Disabilities, 15*(1), 30-34. Exclusion reason: Adult population

Barreto, S. d. O., Freitas, L. C., & Del Prette, Z. A. P. (2011). Social skills in the comorbidity between learning disabilities and behavior problems: A multimodal assessment. *Psico, 42*(4), 503-510. Exclusion reason: Not relevant outcome

Barron, D. A., Molosankwe, I., Romeo, R., & Hassiotis, A. (2013). Urban adolescents with intellectual disability and challenging behaviour: Costs and characteristics during transition to adult services. *Health & Social Care in the Community, 21*(3), 283-292. 10.1111/hsc.12015. Exclusion reason: No psychometric information

Barry-Walsh, J., Daffern, M., Duncan, S., & Ogloff, J. (2009). The prediction of imminent aggression in patients with mental illness and/or intellectual disability using the Dynamic Appraisal of Situational Aggression instrument. *Australasian Psychiatry, 17*(6), 493-496. 10.1080/10398560903289975. Exclusion reason: Results not reported separately for children and adolescents

Barthelemy, C., Adrien, J.-L., Roux, S., Garreau, B., Perrot, A., & Lelord, G. (1992). Sensitivity and specificity of the Behavioral Summarized Evaluation (BSE) for the assessment of autistic behaviors. *Journal of Autism and Developmental Disorders, 22*(1), 23-31. 10.1007/BF01046400. Exclusion reason: Not relevant measurement tool

Barthelemy, C., Roux, S., Adrien, J., Hameury, L., Guerin, P., Garreau, B., . . . Lelord, G. (1997). Validation of the Revised Behavior Summarized Evaluation Scale. *Journal of Autism and Developmental Disorders, 27*(2), 139-153. 10.1023/A:1025887723360. Exclusion reason: Not relevant measurement tool

Basgul, S. S., Etiler, N., Coskun, A., Karakaya, I., & Agaoglu, B. (2009). Reliability and validity of the Turkish version of ECI-4 parent scale. *Cocuk ve Genclik Ruh Sagligi Dergisi, 16*(2), 83-92. Exclusion reason: Wrong patient population

Basile, E., Villa, L., Selicorni, A., & Molteni, M. (2007). The behavioural phenotype of Cornelia de Lange syndrome: A study of 56 individuals. *Journal of Intellectual Disability Research, 51*(9), 671-681. 10.1111/j.1365-2788.2007.00977.x. Exclusion reason: Wrong patient population

Batabre, R., Kale, V. P., Shah, S., & Kadam, M. (2013). The prevalence of perceived stress and coping strategies in parents of intellectually disabled children. *Indian Journal of Psychiatry, 55 (SUPPL.1)*, S45-S46. Exclusion reason: Conference Abstract

Baumgardner, T. L., Reiss, A. L., Freund, L. S., & Abrams, M. T. (1995). Specification of the neurobehavioral phenotype in males with fragile X syndrome. *Pediatrics, 95*(5), 744-752. Exclusion reason: No psychometric information

Bausela Herreras, E. (2019). BRIEF-P: Validation Study in Children in Early Childhood with Neurodevelopmental Disorders. *SAGE Open, 9*(3). Exclusion reason: Not relevant measurement tool

Beck, B., Amon, P., Castell, R., Mall, W., & Wilkes, J. (1993). [Reliability of the Child Behavior Checklist in a population of 6 to 8-year-old special education students]. *Zeitschrift fur Kinder- und Jugendpsychiatrie, 21*(2), 101-108. Exclusion reason: Article in foreign language/not accessible language

Beitchman, J. H., Cantwell, D. P., Forness, S. R., Kavale, K. A., & Kauffman, J. M. (1998). Practice parameters for the assessment and treatment of children and adolescents with language and learning disorders. *Journal of the American Academy of Child & Adolescent Psychiatry, 37*(10, Suppl), 46S-62S. 10.1097/00004583-199810001-00004. Exclusion reason: Review

Bender, W. N. (1985). Differences between learning disabled and non-learning disabled children in temperament and behavior. *Learning Disability Quarterly, 8*(1), 11-18. 10.2307/1510903. Exclusion reason: No psychometric information

Benson, B. A., & Reiss, S. (1984). A factor analysis of emotional disorders in mentally retarded people. *Australia & New Zealand Journal of Developmental Disabilities, 10*(3), 135-139. Exclusion reason: Results not reported separately for children and adolescents

Beran, T. N. (2006). Test Review: Bracken, B. A., & Keith, L. K. (2004). "Clinical Assessment of Behavior." Lutz, FL: Psychological Assessment Resources. *Journal of Psychoeducational Assessment, 24*(4), 399-403. Exclusion reason: No intellectual disability information

Bernard, S. H. (2011). The mental health needs of children with intellectual disabilities. *Mental health in intellectual disabilities: A reader , 4th ed*, 197-205. Exclusion reason: Printed books

Bernard, S. H., Kannabiran, M., & Philips, N. (2010). Assessment. *Mental health needs of children and young people with learning disabilities*, 27-59. Exclusion reason: Theoretical article/Comment

Bernsen, A. H. (1980). An interview technique in assessing retarded children: A comparative study of the reliability of the Children's Handicaps, Behaviour and Skills (HBS) Schedule. *Journal of Mental Deficiency Research, 24*(3), 167-179. Exclusion reason: Not relevant measurement tool

Bhang, S., Kim, J., & Hwang, S. (2015). Assessing problematic behaviors in Korean children with developmental disorders. *European Child and Adolescent Psychiatry, 1)*, S180. 10.1007/s00787-015-0714-4. Exclusion reason: Conference abstract

Bhattacharyya, R., Sanyal, D., Roy, K., & Saha, S. (2009). A study of cluster behavioral abnormalities in Down syndrome. *Indian Journal of Medical Sciences, 63*(2), 58-65. Exclusion reason: Results not reported separately for children and adolescents

Bhuiyan, Z. A., Klein, M., Hammond, P., Van Haeringen, A., Mannens, M. M. A. M., Van Berckelaer-Onnes, I., & Hennekam, R. C. M. (2006). Genotype-phenotype correlations of 39 patients with Cornelia de Lange syndrome: The Dutch experience. *Journal of Medical Genetics, 43*(7), 568-575. 10.1136/jmg.2005.038240. Exclusion reason: Results not reported separately for children and adolescents

Biggs, E. E., & Carter, E. W. (2016). Quality of life for transition-age youth with autism or intellectual disability. *Journal of Autism and Developmental Disorders, 46*(1), 190-204. 10.1007/s10803-015-2563-x. Exclusion reason: Not relevant measurement tool

Bilgic, A., Uslu, R., & Kartal, O. O. (2011). Comparison of toddlers with pervasive developmental disorders and developmental delay based on diagnosis classification: 0-3 Revised. *Noropsikiyatri Arsivi, 48*(3), 188-194. Exclusion reason: Mean age < 4 years

Blacher, J., Baker, B. L., Eisenhower, A. S., Blacher, J., Baker, B. L., & Eisenhower, A. S. (2009). Student-teacher relationship stability across early school years for children with intellectual disability or typical development. *American Journal on Intellectual & Developmental Disabilities, 114*(5), 322-339. Exclusion reason: Not relevant measurement tool

Blacher, J., Howell, E., Lauderdale-Littin, S., DiGennaro Reed, F. D., & Laugeson, E. A. (2014). Autism Spectrum Disorder and the Student Teacher Relationship: A Comparison Study with Peers with Intellectual Disability and Typical Development. *Grantee Submission, 8*, 324-333. Exclusion reason: No psychometric information

Bolourian, Y. R. (2019). Co-occurring behavior problems in youth with intellectual and developmental disabilities: A developmental perspective. *Dissertation Abstracts International Section A: Humanities and Social Sciences, 80*(7-A(E)), No Pagination Specified. Exclusion reason: No psychometric information

Bolte, S., & Poustka, F. (2002). The relation between general cognitive level and adaptive behavior domains in individuals with autism with and without co-morbid mental retardation. *Child Psychiatry and Human Development, 33*(2), 165-172. 10.1023/A:1020734325815. Exclusion reason: Not relevant measurement tool

Bond, M. J., & Tustin, R. (1999). Identification of conduct and emotional syndromes using the Adelaide Behaviour Disorder Scale. *Journal of Intellectual and Developmental Disability, 24*(2), 133-138. 10.1080/13668259900033921. Exclusion reason: Results not reported separately for children and adolescents

Bonfield, J. R., & Pennsylvania State Univ, U. P. (1968). Predictors of Achievement for Educable Mentally Retarded Children. Final Report. In.Borthwick-Duffy, S. A., Lane, K. L., & Widaman, K. F. (1997). Measuring problem behaviors in children with mental retardation: dimensions and predictors. *Research in Developmental Disabilities, 18*(6), 415-433. Exclusion reason: Duplicate reference.

Bostrom, P. (2016). Protection and limitation: Experiences of the context that impact the mental health of young persons with intellectual and developmental disabilities. *Journal of Intellectual Disability Research, 60 (7-8)*, 641. Exclusion reason: Conference Abstract

Bostrom, P., Asberg, J., & Broberg, M. (2014). Development of a self-report mental health questionnaire (WellSEQ) for adolescents with intellectual and developmental disabilities. *Journal of Applied Research in Intellectual Disabilities, 27*(4), 313-313. Exclusion reason: Conference abstract

Bostrom, P., Asberg Johnels, J., & Broberg, M. (2018). Self-reported psychological wellbeing in adolescents: The role of intellectual/developmental disability and gender. *Journal of Intellectual Disability Research, 62*(2), 83-93. 10.1111/jir.12432. Exclusion reason: Not relevant measurement tool

Bostrom, P., & Broberg, M. (2018). Protection and restriction: A mixed-methods study of self-reported well-being among youth with intellectual disabilities. *Journal of Applied Research in Intellectual Disabilities, 31*(1), e164-e176. 10.1111/jar.12364. Exclusion reason: Not relevant outcome

Bostrom, P., Broberg, M., & Hwang, C. (2010). Different, difficult or distinct? Mothers' and fathers' perceptions of temperament in children with and without intellectual disabilities. *Journal of Intellectual Disability Research, 54*(9), 806-819. 10.1111/j.1365-2788.2010.01309.x. Exclusion reason: Not relevant measurement tool

Bourke-Taylor, H., Law, M., Howie, L., & Pallant, J. F. (2010). Development of the Child's Challenging Behaviour Scale (CCBS) for mothers of school-aged children with disabilities. *Child: Care, Health & Development, 36*(4), 491-498. 10.1111/j.1365-2214.2009.01055.x. Exclusion reason: Not relevant measurement tool

Bourke-Taylor, H., Pallant, J., & Law, M. (2014). Update on the child's challenging behaviour scale following evaluation using Rasch analysis. *Child: Care, Health and Development, 40*(2), 242-249. 10.1111/cch.12035. Exclusion reason: Not relevant measurement tool

Bower, E. M. (1969). Early Identification of Emotionally Handicapped Children in School, Second Edition. A Monograph in American Lectures in Psychology. In. Bowring, D. L., Totsika, V., Hastings, R. P., & Toogood, S. (2019). Outcomes from a community-based positive behavioural support team for children and adults with developmental disabilities. *Journal of Applied Research in Intellectual Disabilities*, No Pagination Specified. 10.1111/jar.12660. Exclusion reason: Duplicate reference

Bowring, D. L., Totsika, V., Hastings, R. P., & Toogood, S. (2020). Outcomes from a community-based Positive Behavioural Support team for children and adults with developmental disabilities. *Journal of Applied Research in Intellectual Disabilities, 33*(2), 193-203. 10.1111/jar.12660. Exclusion reason: Results not reported separately for children and adolescents

Bradshaw, J., Gore, N., & Darvell, C. (2018). Supporting the direct involvement of students with disabilities in functional assessment through use of Talking Mats®. *Tizard Learning Disability Review, 23*(2), 111-116. 10.1108/TLDR-01-2018-0004. Exclusion reason: No psychometric information

Bramston, P., & Fogarty, G. (2000). The assessment of emotional distress experienced by people with an intellectual disability: a study of different methodologies. *Research in Developmental Disabilities, 21*(6), 487-500. Exclusion reason: Results not reported separately for children and adolescents

Breau, L. M., Camfield, C. S., Symons, F. J., Bodfish, J. W., Mackay, A., Finley, G. A., & McGrath, P. J. (2003). Relation between pain and self-injurious behavior in nonverbal children with severe cognitive impairments. *Journal of Pediatrics, 142*(5), 498-503. Exclusion reason: Not relevant outcome

Brereton, A. V., Tonge, B. J., Mackinnon, A. J., & Einfeld, S. L. (2002). Screening young people for autism with the developmental behavior checklist. *Journal of the American Academy of Child & Adolescent Psychiatry, 41*(11), 1369-1375. Exclusion reason: Not relevant measurement tool

Breslau, N. (1990). Does brain dysfunction increased children's vulnerability to environmental stress? *Archives of General Psychiatry, 47*(1), 15-20. Exclusion reason: Wrong patient population

Briegel, W., Schneider, M., & Schwab, K. O. (2009). [22q11.2 deletion: handicap-related problems and coping strategies of primary caregivers]. *Zeitschrift fur Kinder-und Jugendpsychiatrie und Psychotherapie, 37*(6), 535-540. 10.1024/1422-4917.37.6.535. Exclusion reason: Not relevant measurement tool

Brier, N. (1986). The mildly retarded adolescent: a psychosocial perspective. *Journal of developmental and behavioral pediatrics : JDBP, 7*(5), 320-323. 10.1097/00004703-198610000-00010. Exclusion reason: Theoretical article/Comment

Brinkley, J., Nations, L., Abramson, R. K., Hall, A., Wright, H. H., Gabriels, R., . . . Cuccaro, M. L. (2007). Factor Analysis of the Aberrant Behavior Checklist in Individuals with Autism Spectrum Disorders. Journal of Autism and Developmental Disorders, 37(10), 1949-1959. 10.1007/s10803-006-0327-3. Exclusion reason: No intellectual disability information.

Brown, E. C., Aman, M. G., & Lecavalier, L. (2004). Empirical Classification of Behavioral and Psychiatric Problems in Children and Adolescents With Mental Retardation. *American Journal on Mental Retardation, 109*(6), 445-455. 10.1352/0895-8017%282004%29109%3C445:ECOBAP%3E2.0.CO;2. Exclusion reason: No psychometric information

Brown, L., Hammill, D. D., & Sherbenou, R. J. (1981). The reliability of four measures of children's behavior with deviant populations. *Behavioral Disorders, 6*(3), 180-182. 10.1177/019874298100600301. Exclusion reason: Wrong patient population

Buchholz, E. (2013). Gender and comorbid psychopathologies in toddlers with autism spectrum disorders. *Dissertation Abstracts International: Section B: The Sciences and Engineering, 74*(5-B(E)), No Pagination Specified. Exclusion reason: Mean age < 4 years

Buelow, J. M., Austin, J. K., Perkins, S. M., Shen, J., Dunn, D. W., & Fastenau, P. S. (2003). Behavior and mental health problems in children with epilepsy and low IQ. *Developmental Medicine & Child Neurology, 45*(10), 683-692. Exclusion reason: No psychometric information

Buelow, J. M., Perkins, S. M., Johnson, C. S., Byars, A. W., Fastenau, P. S., Dunn, D. W., & Austin, J. K. (2012). Adaptive functioning in children with epilepsy and learning problems. *Journal of Child Neurology, 27*(10), 1241-1249. 10.1177/0883073811432750. Exclusion reason: No psychometric information

Bull, L. E., Oliver, C., Tunnicliffe, P. L., & Woodcock, K. A. (2015). An informant report behavior diary for measuring temper outbursts in an intervention setting. *Journal of Developmental and Physical Disabilities, 27*(4), 489-504. 10.1007/s10882-015-9429-1. Exclusion reason: Not relevant measurement tool

Bullock, W. A., & et al. (1995). *Intellectual, Achievement, and Mental Health Evaluation of At-Risk Adolescents: Assessing Comorbidity of ADHD, LD, and Conduct Problems*. Retrieved from http://search.ebscohost.com/login.aspx?direct=true&db=eric&AN=ED389817&site=ehost-live Exclusion reason: Wrong patient population

Burakevych, N., McKinlay, C. J. D., Alsweiler, J. M., Wouldes, T. A., & Harding, J. E. (2016). Pre-school screening for developmental and emotional health: Comparison with neurodevelopmental assessment. *Journal of Paediatrics and Child Health, 52*(6), 600-607. 10.1111/jpc.13169. Exclusion reason: Not relevant measurement tool

Burbidge, C., Oliver, C., Moss, J., Arron, K., Berg, K., Furniss, F., . . . Woodock, K. (2010). The association between repetitive behaviours, impulsivity and hyperactivity in people with intellectual disability. *Journal of Intellectual Disability Research, 54*(12), 1078-1092. 10.1111/j.1365-2788.2010.01338.x. Exclusion reason: Results not reported separately for children and adolescents

Burdett, K. M. (1996). A comparison of the psychological wellness of Chapter I students to learning disabled and regular education students. *Dissertation Abstracts International Section A: Humanities and Social Sciences, 56*(12-A), 4724. Exclusion reason: Unable to obtain in full text

Burns, G., & Patterson, D. R. (1990). Conduct problem behaviors in a stratified random sample of children and adolescents: New standardization data on the Eyberg Child Behavior Inventory. *Psychological Assessment: A Journal of Consulting and Clinical Psychology, 2*(4), 391-397. 10.1037/1040-3590.2.4.391. Exclusion reason: Wrong patient population

Burns, G., & Patterson, D. R. (1991). Factor structure of the Eyberg Child Behavior Inventory: Unidimensional or multidimensional measure of disruptive behavior? *Journal of Clinical Child Psychology, 20*(4), 439-444. 10.1207/s15374424jccp2004_13. Exclusion reason: No intellectual disability information

Burns, G., Patterson, D. R., Nussbaum, B. R., & Parker, C. M. (1991). Disruptive behaviors in an outpatient pediatric population: Additional standardization data on the Eyberg Child Behavior Inventory. *Psychological Assessment: A Journal of Consulting and Clinical Psychology, 3*(2), 202-207. 10.1037/1040-3590.3.2.202. Exclusion reason: Wrong patient population

Byrne, J. M., Backman, J. E., Gates, R. D., & Clark-Touesnard, M. (1986). Interpretation of the Personality Inventory for Children-Revised (PIC-R): influence of cognitive impairment. *Journal of Abnormal Child Psychology, 14*(2), 287-296. Exclusion reason: Not relevant measurement tool

Calkins, M. E., Merikangas, K. R., Burstein, M., Satterthwaite, T., Wolf, D., Moore, T. M., . . . Gur, R. E. (2015). Clinical phenotypic characterization of the philadelphia neurodevelopmental cohort: Foundations for integrative investigations of psychiatric disorders. *Biological Psychiatry, 1)*, 288S-289S. Exclusion reason: Conference abstract

Calkins, M. E., Merikangas, K. R., Moore, T. M., Burstein, M., Behr, M. A., Satterthwaite, T. D., . . . Gur, R. E. (2015). The Philadelphia neurodevelopmental cohort: Constructing a deep phenotyping collaborative. *Journal of Child Psychology and Psychiatry, 56*(12), 1356-1369. 10.1111/jcpp.12416. Exclusion reason: Wrong patient population

Camfield, C., Breau, L., Camfield, P., Camfield, C., Breau, L., & Camfield, P. (2003). Assessing the impact of pediatric epilepsy and concomitant behavioral, cognitive, and physical/neurologic disability: Impact of Childhood Neurologic Disability Scale. *Developmental Medicine & Child Neurology, 45*(3), 152-159. Exclusion reason: Not relevant measurement tool

Campbell, L. E., Daly, E., Toal, F., Stevens, A., Azuma, R., Catani, M., . . . Murphy, K. C. (2006). Brain and behaviour in children with 22q11.2 deletion syndrome: a volumetric and voxel-based morphometry MRI study. *Brain, 129*(Pt 5), 1218-1228. Exclusion reason: No psychometric information

Campbell, M., & Malone, R. P. (1991). MENTAL-RETARDATION AND PSYCHIATRIC-DISORDERS. *Hospital and Community Psychiatry, 42*(4), 374-379. Exclusion reason: Theoretical article/Comment

Caplan, B., Neece, C. L., & Baker, B. L. (2015). Developmental level and psychopathology: Comparing children with developmental delays to chronological and mental age matched controls. *Research in Developmental Disabilities, 37*, 143-151. 10.1016/j.ridd.2014.10.045. Exclusion reason: No psychometric information

Capone, G., Goyal, P., Ares, W., & Lannigan, E. (2006). Neurobehavioral disorders in children, adolescents, and young adults with Down syndrome. *American Journal of Medical Genetics, Part C: Seminars in Medical Genetics, 142*(3), 158-172. 10.1002/ajmg.c.30097. Exclusion reason: Theoretical article/Comment

Capone, G. T., Aidikoff, J. M., & Goyal, P. (2011). Adolescents and young adults with Down syndrome presenting to a medical clinic with depression: Phenomenology and characterization using the Reiss Scales and Aberrant Behavior Checklist. *Journal of Mental Health Research in Intellectual Disabilities, 4*(4), 244-264. 10.1080/19315864.2011.599917. Exclusion reason: Results not reported separately for children and adolescents

Caraveo-Anduaga, J. J. (2007). Validity of the Brief Screening and Diagnostic Questionnaire (CBTD) for children and adolescents in clinical settings. *Salud Mental, 30*(2), 42-49. Exclusion reason: Wrong patient population

Carberry, A. T., & Handal, P. J. (1980). The use of the AML Scale with a Headstart population: Normative and validation studies. *American Journal of Community Psychology, 8*(3), 353-363. 10.1007/BF00894347. Exclusion reason: No intellectual disability information

Carcani-Rathwell, I., Rabe-Hasketh, S., & Santosh, P. J. (2006). Repetitive and stereotyped behaviours in pervasive developmental disorders. *Journal of Child Psychology & Psychiatry, 47*(6), 573-581. Exclusion reason: Not relevant measurement tool

Carmeli, E., Klein, N., & Sohn, M. (2007). The implications of having attention-deficit/hyperactivity disorder in male adolescents with intellectual disability. *International Journal of Adolescent Medicine and Health, 19*(2), 209-214. 10.1515/IJAMH.2007.19.2.209. Exclusion reason: No psychometric information

Carnazzo, K., Dowdy, E., Furlong, M. J., & Quirk, M. P. (2019). An evaluation of the Social Emotional Health SurveySecondary for use with students with learning disabilities. *Psychology in the Schools, 56*(3), 433-446. 10.1002/pits.22199. Exclusion reason: Not relevant outcome

Carnazzo, K. W. (2018). An evaluation of the Social Emotional Health Survey-Secondary for use with students with learning disabilities: Confirmatory factor analysis, measurement invariance, and comparative analyses. *Dissertation Abstracts International Section A: Humanities and Social Sciences, 78*(12-A(E)), No Pagination Specified. Exclusion reason: Not relevant outcome

Carter, A., & Briggs-Gowan, M. (2011). The Brief-Infant Toddler Social and Emotional Assessment (BITSEA): Clinical validation. *European Child and Adolescent Psychiatry, 1)*, S46. 10.1007/s00787-011-0181-5. Exclusion reason: Conference abstract

Caruana, J., Dossetor, D., Eisler, K., & Saleh, H. (2019). I thought you would never ask!: The english translation of wellseq, a self- report measure of well-being for adolescents with intellectual or developmental disability. *Journal of Intellectual Disability Research, 63 (7)*, 684. 10.1111/jir.12653. Exclusion reason: Conference abstract

Catani, C., & Sossalla, I. M. (2015). Child abuse predicts adult PTSD symptoms among individuals diagnosed with intellectual disabilities. *Frontiers in Psychology Vol 6 2015, ArtID 1600, 6*. Exclusion reason: Adult population.

Cervi, F., Vignoli, A., & La Briola, F. (2019). New strategies to assess neuropsychiatric involvement and improve the outcome in children and adolescents with NF1 and TSC. *Neuropediatrics. Conference: 47th Annual Meeting of the Societe Europeenne de Neurologie Pediatrique, SENP, 50*(Supplement 1). 10.1055/s00942716. Exclusion reason: Not relevant outcome

Chadwick, O., Kusel, Y., & Cuddy, M. (2008). Factors associated with the risk of behaviour problems in adolescents with severe intellectual disabilities. *Journal of Intellectual Disability Research, 52*(10), 864-876. Exclusion reason: No psychometric information

Chadwick, O., Kusel, Y., Cuddy, M., & Taylor, E. (2005). Psychiatric diagnoses and behaviour problems from childhood to early adolescence in young people with severe intellectual disabilities. *Psychological Medicine, 35*(5), 751-760. 10.1017/S0033291704003733. Exclusion reason: No psychometric information

Charlot, L., Deutsch, C., Hunt, A., Fletcher, K., & McLlvane, W. (2007). Validation of the Mood and Anxiety Semi-structured (MASS) Interview for patients with intellectual disabilities. *Journal of Intellectual Disability Research, 51*, 821-834. 10.1111/j.1365-2788.2007.00972.x. Exclusion reason: Results not reported separately for children and adolescents

Chaturvedi, P., Agarwal, A., & Gupta, S. (1984). Psychological study of inmates of a children's home with special reference to their intelligence and aggressive behaviour. *Indian Journal of Psychiatry, 26*(2), 133-140. Exclusion reason: Results not reported separately for children and adolescents

Cheramie, G. M. (1994). The AAMD Adaptive Behavior Scale--School Edition: II. Test-retest reliability and parent-teacher agreement in a behavior disordered sample. *Perceptual and Motor Skills, 79*(1, Pt 1), 275-283. 10.2466/pms.1994.79.1.275. Exclusion reason: Wrong patient population

Chiarello, L. A., Almasri, N., & Palisano, R. J. (2009). Factors related to adaptive behavior in children with cerebral palsy. *Journal of Developmental and Behavioral Pediatrics, 30*(5), 426-434. 10.1097/DBP.0b013e3181b4ec54. Exclusion reason: Wrong patient population

Chowdhury, M. (2013). Follow-up of maladaptive behaviors in youth with autism spectrum disorders: Changes and predictors over two to eight years. *Dissertation Abstracts International: Section B: The Sciences and Engineering, 74*(3-B(E)), No Pagination Specified. Exclusion reason: No intellectual disability information

Chowdhury, M., Aman, M., Scahill, L., Swiezy, N., Arnold, L., Lecavalier, L., . . . McDougle, C. (2010). The Home Situations Questionnaire-PDD version: Factor structure and psychometric properties. *Journal of Intellectual Disability Research, 54*(3), 281-291. 10.1111/j.1365-2788.2010.01259.x. Exclusion reason: Not relevant measurement tool

Chowdhury, M., Aman, M. G., Lecavalier, L., Smith, T., Johnson, C., Swiezy, N., . . . Scahill, L. (2016). Factor structure and psychometric properties of the revised Home Situations Questionnaire for autism spectrum disorder: The Home Situations Questionnaire-Autism Spectrum Disorder. *Autism, 20*(5), 528-537. 10.1177/1362361315593941. Exclusion reason: Wrong patient population

Christensen, L., Baker, B. L., & Blacher, J. (2013). Oppositional Defiant Disorder in Children With Intellectual Disabilities. *Journal of Mental Health Research in Intellectual Disabilities, 6*(3), 225-244. 10.1080/19315864.2012.661033. Exclusion reason: No psychometric information

Christensen, L. L. (2013). Dual diagnosis: Intellectual disability and oppositional defiant disorder. *Dissertation Abstracts International: Section B: The Sciences and Engineering, 74*(1-B(E)), No Pagination Specified. Exclusion reason: Not relevant measurement tool

Clapp, R. B., Jr. (2015). Demographic variables and intelligence test scores in disability applicants. *Dissertation Abstracts International: Section B: The Sciences and Engineering, 76*(5-B(E)), No Pagination Specified. Exclusion reason: Not relevant measurement tool

Clark, D., & Wilson, G. N. (2003). Behavioral assessment of children with Down syndrome using the Reiss psychopathology scale. *American Journal of Medical Genetics. Part A, 118A*(3), 210-216. Exclusion reason: Duplicate reference

Clark, D., & Wilson, G. N. (2003). Behavioral assessment of children with Down syndrome using the Reiss psychopathology scale. *American Journal of Medical Genetics, 118 A*(3), 210-216. Exclusion reason: No psychometric information

Clark, E. (1982). Construct validity and diagnostic potential of the personality inventory for children (PIC) with emotionally disturbed, learning disabled, and educable mentally retarded children. *Dissertation Abstracts International, 43*(5-A), 1473-1474. Exclusion reason: Not relevant measurement tool

Clark, E. (1987). Responses of mothers and fathers on the Personality Inventory for Children: Are they significantly different? *Journal of Psychoeducational Assessment, 5*(2), 138-148. 10.1177/073428298700500205. Exclusion reason: Not relevant measurement tool

Clark, E., Kehle, T. J., Bullock, D., & Jenson, W. R. (1987). Convergent and discriminant validity of the personality inventory for children. *Journal of Psychoeducational Assessment, 5*(2), 99-106. 10.1177/073428298700500202. Exclusion reason: Not relevant measurement tool

Clark, E., Kehle, T. J., & Bullock, D. S. (1988). Personality Inventory for Children: Profiles for learning disabled, emotionally disturbed, and intellectually handicapped children. *School Psychology International, 9*(1), 43-49. 10.1177/0143034388091007. Exclusion reason: Not relevant measurement tool

Clarke, D. J., & Boer, H. (1998). Problem behaviors associated with deletion Prader-Willi, Smith-Magenis, and cri du chat syndromes. *American Journal on Mental Retardation, 103*(3), 264-271. 10.1352/0895-8017%281998%29103%3C0264:PBAWDP%3E2.0.CO;2. Exclusion reason: Results not reported separately for children and adolescents

Cochran, L., Welham, A., Oliver, C., Arshad, A., & Moss, J. F. (2019). Age-related behavioural change in Cornelia de Lange and Cri du Chat syndromes: A seven year follow-up study. *Journal of Autism and Developmental Disorders, 49*(6), 2476-2487. 10.1007/s10803-019-03966-6. Exclusion reason: No psychometric information

Coe, D. A. (1995). An investigation of behavior problems of children with Down Syndrome and their relationship to life events. *Dissertation Abstracts International: Section B: The Sciences and Engineering, 56*(3-B), 1694. Exclusion reason: Duplicate reference

Cohen, I. L., Schmidt-Lackner, S., Romanczyk, R., & Sudhalter, V. (2003). The PDD behavior inventory: A rating scale for assessing response to intervention in children with pervasive developmental disorder. *Journal of Autism and Developmental Disorders, 33*(1), 31-45. 10.1023/A:1022226403878. Exclusion reason: Not relevant measurement tool

Cohen, M., & Hynd, G. W. (1986). The Conners Teacher Rating Scale: A different factor structure with special education children. *Psychology in the Schools, 23*(1), 13-23. 10.1002/1520-6807%28198601%2923:1%3C13::AID-PITS2310230103%3E3.0.CO;2-5. Exclusion reason: Not relevant measurement tool

Cohen, N. J., Gotlieb, H., Kershner, J., & Wehrspann, W. (1985). Concurrent validity of the internalizing and externalizing profile patterns of the Achenbach Child Behavior Checklist. *Journal of Consulting & Clinical Psychology, 53*(5), 724-728. Exclusion reason: No intellectual disability information

Cohen, N. J., Kolers, N., & Bradley, S. (1990). Relation of global ratings of functioning with behaviour and development in delayed and disturbed preschoolers. *Canadian Journal of Psychiatry - Revue Canadienne de Psychiatrie, 35*(6), 514-518. Exclusion reason: Not relevant outcome

Colmar, S., Maxwell, A., & Miller, L. (2006). Assessing intellectual disability in children: Are IQ measures sufficient, or even necessary? *Australian Journal of Guidance and Counselling, 16*(2), 177-188. 10.1375/ajgc.16.2.177. Exclusion reason: Theoretical article/Comment

Colombo, P., Nobile, M., Tesei, A., Civati, F., Gandossini, S., Mani, E., . . . D'Angelo, G. (2017). Assessing mental health in boys with Duchenne muscular dystrophy: Emotional, behavioural and neurodevelopmental profile in an Italian clinical sample. *European Journal of Paediatric Neurology, 21*(4), 639-647. 10.1016/j.ejpn.2017.02.007. Exclusion reason: No psychometric information

Conroy, M., Fox, J., Crain, L., Jenkins, A., & et al. (1996). Evaluating the social and ecological validity of analog assessment procedures for challenging behaviors in young children. *Education and Treatment of Children, 19*(3), 233-256. Exclusion reason: Not relevant measurement tool

Consoli, A., Cohen, J., Bodeau, N., Guinchat, V., Wachtel, L., & Cohen, D. (2013). Electroconvulsive therapy in adolescents with intellectual disability and severe self-injurious behavior and aggression: A retrospective study. *European Child & Adolescent Psychiatry, 22*(1), 55-62. 10.1007/s00787-012-0320-7. Exclusion reason: No psychometric information

Constantino, J. N., Przybeck, T., Friesen, D., & Todd, R. D. (2000). Reciprocal social behavior in children with and without pervasive developmental disorders. *Journal of Developmental & Behavioral Pediatrics, 21*(1), 2-11. Exclusion reason: Not relevant measurement tool

Conway, K. M. (2005). An evaluation of social skills training for youth with learning disabilities. *Dissertation Abstracts International: Section B: The Sciences and Engineering, 65*(9-B), 4822. Exclusion reason: Not relevant outcome

Coolidge, F. L., Thede, L. L., Stewart, S. E., & Segal, D. L. (2002). The Coolidge Personality and Neuropsychological Inventory for Children (CPNI): Preliminary psychometric characteristics. *Behavior Modification, 26*(4), 550-566. 10.1177/0145445502026004007. Exclusion reason: No intellectual disability information

Copeland, W. E., Simonoff, E., & Stringaris, A. (2016). Disruptive mood dysregulation disorder in children with autism spectrum disorder. *Journal of the American Academy of Child and Adolescent Psychiatry, 55 (10 Supplement 1)*, S269-S270. 10.1016/j.jaac.2016.07.164. Exclusion reason: No intellectual disability information

Coppola, G., Verrotti, A., Resicato, G., Ferrarelli, S., Auricchio, G., Operto, F. F., & Pascotto, A. (2008). Topiramate in children and adolescents with epilepsy and mental retardation: a prospective study on behavior and cognitive effects. *Epilepsy & Behavior, 12*(2), 253-256. Exclusion reason: Not relevant measurement tool

Cordeiro, L., Abucayan, F., Hagerman, R., Tassone, F., & Hessl, D. (2015). Anxiety disorders in fragile X premutation carriers: Preliminary characterization of probands and non-probands. *Irdr, 4*(3), 123-130. 10.5582/irdr.2015.01029. Exclusion reason: Results not reported separately for children and adolescents

Cordeiro, L., Ballinger, E., Hagerman, R., & Hessl, D. (2011). Clinical assessment of DSM-IV anxiety disorders in fragile X syndrome: Prevalence and characterization. *Journal of Neurodevelopmental Disorders, 3*(1), 57-67. 10.1007/s11689-010-9067-y. Exclusion reason: Results not reported separately for children and adolescents

Cornish, K., Munir, F., & Wilding, J. (2001). A neuropsychological and behavioural profile of attention deficits in fragile X syndrome. [Spanish]. *Revista de Neurologia, 33 Suppl 1*, S24-29. Exclusion reason: Conference Abstract

Cornish, K., Munir, F., & Wilding, J. (2001). [A neuropsychological and behavioural profile of attention deficits in fragile X syndrome]. *Revista de Neurologia, 33 Suppl 1*, S24-29. Exclusion reason: Duplicate reference

Costenbader, V. K., & Keller, H. R. (1990). Behavioral ratings of emotionally handicapped, learning disabled, and nonreferred children: Scale and source consistency. *Journal of Psychoeducational Assessment, 8*(4), 485-496. 10.1177/073428299000800404. Exclusion reason: No psychometric information

Coughlin, M., Sharry, J., Fitzpatrick, C., Guerin, S., & Drumm, M. (2009). A controlled clinical evaluation of the Parents Plus Children's Programme: a video-based programme for parents of children aged 6 to 11 with behavioural and developmental problems. *Clinical Child Psychology & Psychiatry, 14*(4), 541-558. 10.1177/1359104509339081. Exclusion reason: No psychometric information

Coutinho, V., Kemlin, I., Dorison, N., Billette de Villemeur, T., Rodriguez, D., & Dellatolas, G. (2016). Neuropsychological evaluation and parental assessment of behavioral and motor difficulties in children with neurofibromatosis type 1. *Research in Developmental Disabilities, 48*, 220-230. 10.1016/j.ridd.2015.11.010. Exclusion reason: Wrong patient population

Cowell, D. (1993). Screening for behaviour problems in a school for pupils with moderate learning difficulties. *AEP (Association of Educational Psychologists) Journal, 9*(2), 105-110. Exclusion reason: No intellectual disability information

Crews, W. D., Jr., Bonaventura, S., & Rowe, F. (1994). Dual diagnosis: prevalence of psychiatric disorders in a large state residential facility for individuals with mental retardation. *American Journal of Mental Retardation, 98*(6), 724-731. Exclusion reason: Not relevant outcome

Cullinan, D., & Epstein, M. H. (1985). Adjustment problems of mildly handicapped and nonhandicapped students. *RASE: Remedial & Special Education, 6*(2), 5-11. 10.1177/074193258500600203. Exclusion reason: No psychometric information

Cullinan, D., Epstein, M. H., & Lloyd, J. (1981). School behavior problems of learning disabled and normal girls and boys. *Learning Disability Quarterly, 4*(2), 163-169. 10.2307/1511001. Exclusion reason: Wrong patient population

Cullinan, D., Epstein, M. H., & Olinger, E. (1983). School behavior problems of mentally retarded and normal females. *Mental Retardation & Learning Disability Bulletin, 11*(3), 104-109. Exclusion reason: No psychometric information

Cullinan, D., & et al. (1979). Behavior Problems of Educationally Handicapped and Normal Pupils. *Journal of Abnormal Child Psychology, 7*(4), 495-502. Exclusion reason: Published prior to 1980

Cullinan, D., & et al. (1984). Behavior Problems of Mentally Retarded and Nonretarded Adolescent Pupils. *School Psychology Review, 13*(3), 381-384. Exclusion reason: No psychometric information

Cullinan, D., Gadow, K. D., & Epstein, M. H. (1987). Psychotropic drug treatment among learning-disabled, educable mentally retarded, and seriously emotionally disturbed students. *Journal of Abnormal Child Psychology, 15*(4), 469-477. 10.1007/BF00917234. Exclusion reason: Outcome: psychopharmacology

Cullinan, D., Schultz, R. M., Epstein, M. H., & Luebke, J. F. (1984). Behavior problems of handicapped adolescent female students. *Journal of Youth and Adolescence, 13*(1), 57-64. 10.1007/BF02088653. Exclusion reason: Wrong patient population

Curfs, L. M., Verhulst, F. C., & Fryns, J. P. (1991). Behavioral and emotional problems in youngsters with Prader-Willi syndrome. *Genetic Counseling, 2*(1), 33-41. Exclusion reason: Results not reported separately for children and adolescents

Curry, J. F., & Thompson, R. J. (1982). Patterns of behavioral disturbance in developmentally disabled children: A replicated cluster analysis. *Journal of Pediatric Psychology, 7*(1), 61-73. 10.1093/jpepsy/7.1.61. Exclusion reason: No intellectual disability information

Cuskelly, M., & Dadds, M. (1992). BEHAVIORAL-PROBLEMS IN CHILDREN WITH DOWNS-SYNDROME AND THEIR SIBLINGS. *Journal of Child Psychology and Psychiatry and Allied Disciplines, 33*(4), 749-761. 10.1111/j.1469-7610.1992.tb00910.x. Exclusion reason: Duplicate reference

Cuskelly, M., & Dadds, M. (1992). Behavioural problems in children with Down's syndrome and their siblings. *Journal of Child Psychology & Psychiatry & Allied Disciplines, 33*(4), 749-761. Exclusion reason: No psychometric information

Cuskelly, M., & Gunn, P. (1993). Maternal reports of behavior of siblings of children with Down syndrome. *American Journal of Mental Retardation, 97*(5), 521-529. Exclusion reason: Not relevant outcome

Dagnan, D. (2011). Phobias and anxiety-related problems in mental retardation and developmental disabilities. *Handbook of child and adolescent anxiety disorders*, 435-446. 10.1007/978-1-4419-7784-7_29. Exclusion reason: Theoretical article/Comment

Dale, N., Salt, A., Sakkalou, E., Glew, S., Eriksson, M., & Clarkson, H. (2018). Investigation of a new social communication observation schedule for assessment of autism spectrum disorder in young children with profound-severe visual impairment. *Developmental Medicine and Child Neurology, 60 (Supplement 2)*, 13. 10.1111/dmcn.13789. Exclusion reason: Not relevant measurement tool

Dall'Oglio, A. M., Rossiello, B., Coletti, M. F., Caselli, M. C., Rava, L., di Ciommo, V., . . . Pasqualetti, P. (2010). Developmental evaluation at age 4: Validity of an Italian parental questionnaire. *Journal of Paediatrics and Child Health, 46*(7-8), 419-426. 10.1111/j.1440-1754.2010.01748.x. Exclusion reason: Not relevant measurement tool

Danielsson, S., Viggedal, G., Steffenburg, S., Rydenhag, B., Gillberg, C., & Olsson, I. (2009). Psychopathology, psychosocial functioning, and IQ before and after epilepsy surgery in children with drug-resistant epilepsy. *Epilepsy & Behavior, 14*(2), 330-337. 10.1016/j.yebeh.2008.10.023. Exclusion reason: Results not reported separately for children and adolescents

Danov, S. E., Tervo, R., Meyers, S., & Symons, F. J. (2012). Using functional analysis methodology to evaluate effects of an atypical antipsychotic on severe problem behavior. *Journal of Mental Health Research in Intellectual Disabilities, 5*(3-4), 286-308. 10.1080/19315864.2011.594976. Exclusion reason: Outcome: psychopharmacology

Daraiseh, N. M., Summerville, L. A., Lin, L., Tucker, D., Hill, A. K., Salisbury, K., & Lind, M. A. (2018). Selection of employee personal protective equipment based on aggressive behavior in pediatric neuropsychiatry. *Developmental Neurorehabilitation, 21*(1), 32-39. 10.1080/17518423.2016.1238968. Exclusion reason: Not relevant outcome

Daveney, J., Hassiotis, A., Katona, C., Matcham, F., & Sen, P. (2019). Ascertainment and Prevalence of Post-Traumatic Stress Disorder (PTSD) in People with Intellectual Disabilities. *Journal of Mental Health Research in Intellectual Disabilities, 12*(3-4), 211-233. 10.1080/19315864.2019.1637979. Exclusion reason: Review

David, M., Dieterich, K., de Villemeur, A., Jouk, P., Counillon, J., Larroque, B., . . . Cans, C. (2014). Prevalence and characteristics of children with mild intellectual disability in a French county. *Journal of Intellectual Disability Research, 58*(7), 591-602. 10.1111/jir.12057. Exclusion reason: Not relevant measurement tool

Davidsson, M., Hult, N., Gillberg, C., Sarneo, C., Gillberg, C., & Billstedt, E. (2017). Anxiety and depression in adolescents with ADHD and autism spectrum disorders; correlation between parent- and self-reports and with attention and adaptive functioning. *Nordic Journal of Psychiatry, 71*(8), 614-620. 10.1080/08039488.2017.1367840. Exclusion reason: Wrong patient population

Davis, T. E., Moree, B. N., Dempsey, T., Reuther, E. T., Fodstad, J. C., Hess, J. A., . . . Matson, J. L. (2011). The relationship between autism spectrum disorders and anxiety: The moderating effect of communication. *Research in Autism Spectrum Disorders, 5*(1), 324-329. 10.1016/j.rasd.2010.04.015. Exclusion reason: No intellectual disability information

de Bildt, A., Kraijer, D., Sytema, S., & Minderaa, R. (2005). The psychometric properties of the Vineland Adaptive Behavior Scales in children and adolescents with mental retardation. *Journal of Autism & Developmental Disorders, 35*(1), 53-62. Exclusion reason: Not relevant measurement tool

de Bildt, A., Mulder, E. J., Hoekstra, P. J., van Lang, N. D. J., Minderaa, R. B., & Hartman, C. A. (2009). Validity of the Children's Social Behavior Questionnaire (CSBQ) in children with intellectual disability: comparing the CSBQ with ADI-R, ADOS, and clinical DSM-IV-TR classification. *Journal of Autism & Developmental Disorders, 39*(10), 1464-1470. 10.1007/s10803-009-0764-x. Exclusion reason: Not relevant measurement tool

de Bildt, A., Mulder, E. J., Scheers, T., Minderaa, R. B., & Tobi, H. (2006). Pervasive developmental disorder, behavior problems, and psychotropic drug use in children and adolescents with mental retardation. *Pediatrics, 118*(6), E1860-E1866. 10.1542/peds.2005-3101. Exclusion reason: No psychometric information

de Bildt, A., Sytema, S., Kraijer, D., Sparrow, S., & Minderaa, R. (2005). Adaptive functioning and behaviour problems in relation to level of education in children and adolescents with intellectual disability. *Journal of Intellectual Disability Research, 49*(9), 672-681. Exclusion reason: No psychometric information

de Boer, L., Röder, I., & Wit, J. M. (2006). Psychosocial, cognitive, and motor functioning in patients with suspected Sotos syndrome: a comparison between patients with and without NSD1 gene alterations. *Developmental Medicine & Child Neurology, 48*(7), 582-588. Exclusion reason: Wrong patient population

De Bruin, E. I., Ferdinand, R. F., Meester, S., De Nijs, P. F. A., & Verheij, F. (2007). High rates of psychiatric co-morbidity in PDD-NOS. *Journal of Autism and Developmental Disorders, 37*(5), 877-886. 10.1007/s10803-006-0215-x. Exclusion reason: Wrong patient population

de Ruiter, K. P., Dekker, M. C., Douma, J. C., Verhulst, F. C., & Koot, H. M. (2008). Development of parent- and teacher-reported emotional and behavioural problems in young people with intellectual disabilities: Does level of intellectual disability matter? *Journal of Applied Research in Intellectual Disabilities, 21*(1), 70-80. Exclusion reason: No psychometric information

De Smedt, B., Devriendt, K., Fryns, J., Vogels, A., Gewillig, M., & Swillen, A. (2007). Intellectual abilities in a large sample of children with Velo-Cardio-Facial Syndrome: an update. *Journal of Intellectual Disability Research, 51*(9), 666-670. Exclusion reason: No psychometric information

de Vries, P. J., Belousova, E., Benedik, M. P., Carter, T., Cottin, V., Curatolo, P., . . . O’Callaghan, F. (2018). TSC-associated neuropsychiatric disorders (TAND): findings from the TOSCA natural history study. *Orphanet Journal Of Rare Diseases, 13*(1), N.PAG-N.PAG. 10.1186/s13023-018-0901-8. Exclusion reason: No psychometric information

de Wolff, M. S., Theunissen, M. H., Vogels, A. G., & Reijneveld, S. A. (2013). Three questionnaires to detect psychosocial problems in toddlers: a comparison of the BITSEA, ASQ:SE, and KIPPPI. *Academic pediatrics, 13*(6), 587-592. 10.1016/j.acap.2013.07.007. Exclusion reason: Wrong patient population

Dekker, M. C., & Koot, H. M. (2003). DSM-IV Disorders in Children With Borderline to Moderate Intellectual Disability. II: Child and Family Predictors. *Journal of the American Academy of Child & Adolescent Psychiatry, 42*(8), 923-931. 10.1097/01.CHI.0000046891.27264.C1. Exclusion reason: No psychometric information

Dekker, M. C., & Koot, H. M. (2004). Problems with emotions and behaviour in young people with intellectual disability. *Kind en Adolescent, 25*(3), 211-223. 10.1007/BF03060916. Exclusion reason: Article in foreign language/not accessible language

Dekker, M. C., Nunn, R., & Koot, H. M. (2002). Psychometric properties of the revised Developmental Behaviour Checklist scales in Dutch children with intellectual disability [corrected] [published erratum appears in J INTELLECT DISABIL RES 2002 Mar;46(part 3):285]. *Journal of Intellectual Disability Research, 46*(1), 61-75. Exclusion reason: No psychometric information

Dekker, M. C., Nunn, R. J., Einfeld, S. E., Tonge, B. J., & Koot, H. M. (2002). Assessing Emotional and Behavioral Problems in Children with Intellectual Disability: Revisiting the Factor Structure of the Developmental Behaviour Checklist. *Journal of Autism and Developmental Disorders, 32*(6), 601-610. Exclusion reason: Duplicate reference

Delamater, A. M., & Lahey, B. B. (1983). Physiological correlates of conduct problems and anxiety in hyperactive and learning-disabled children. *Journal of Abnormal Child Psychology, 11*(1), 85-100. 10.1007/BF00912180. Exclusion reason: No psychometric information

Delforterie, M., Hesper, B., & Didden, R. (2018). Psychometric properties of the dynamic risk outcome scales (dros) for individuals with mild intellectual disability or borderline intellectual functioning and externalizing behaviour problems. *Journal of Applied Research in Intellectual Disabilities*, No Pagination Specified. 10.1111/jar.12546. Exclusion reason: Results not reported separately for children and adolescents

Demb, H. B., Brier, N., Huron, R., & Tomor, E. (1994). The Adolescent Behavior Checklist: normative data and sensitivity and specificity of a screening tool for diagnosable psychiatric disorders in adolescents with mental retardation and other development disabilities. *Research in Developmental Disabilities, 15*(2), 151-165. Exclusion reason: Wrong patient population

Demb, H. B., Brier, N., Huron, R., & Tomor, E. (1994). The Adolescent Behavior Checklist: Normative data and sensitivity and specificity of a screening tool for diagnosable psychiatric disorders in adolescents with mental retardation and other developmental disabilities. *Research in Developmental Disabilities, 15*(2), 151-165. 10.1016/0891-4222%2894%2990019-1. Exclusion reason: Wrong patient population

Demb, H. B. B. N. H. R. T. E. (1994). Adolescent Behavior Checklist. In.Descheemaeker, M. J., Vogels, A., Govers, V., Borghgraef, M., Willekens, D., Swillen, A., . . . Fryns, J. P. (2002). Prader-Willi syndrome: new insights in the behavioural and psychiatric spectrum. *Journal of Intellectual Disability Research, 46*(Pt 1), 41-50. Exclusion reason: No psychometric information

Di Nuovo, S. F., & Buono, S. (2007). Psychiatric syndromes comorbid with mental retardation: Differences in cognitive and adaptive skills. *Journal of Psychiatric Research, 41*(9), 795-800. 10.1016/j.jpsychires.2006.02.011. Exclusion reason: No psychometric information

Di Nuovo, S. F., Buono, S., Colucci, G., & Pellicciotta, A. (2004). Psychopathology and mental retardation: a study using the Rorschach Inkblot Test. *Psychological Reports, 94*(3 Pt 2), 1313-1321. Exclusion reason: Results not reported separately for children and adolescents

Dickson, K., Emerson, E., & Hatton, C. (2005). Self-reported anti-social behaviour: Prevalence and risk factors amongst adolescents with and without intellectual disability. *Journal of Intellectual Disability Research, 49*(11), 820-826. 10.1111/j.1365-2788.2005.00727.x. Exclusion reason: No psychometric information

Dietz, K. R., Lavigne, J. V., Arend, R., & Rosenbaum, D. (1997). Relation between intelligence and psychopathology among preschoolers. *Journal of Clinical Child Psychology, 26*(1), 99-107. Exclusion reason: Wrong patient population

Dietz, S., & Montague, M. (2006). Attention deficit hyperactivity disorder comorbid with emotional and behavioral disorders and learning disabilities in adolescents. *Exceptionality, 14*(1), 19-33. 10.1207/s15327035ex1401_3. Exclusion reason: Wrong patient population

DiLorenzo, T. M., & Michelson, L. (1982). Psychometric properties of the Behavioral Assertiveness Test for Children (BAT-C). *Child & Family Behavior Therapy, 4*(4), 71-76. 10.1300/J019v04n04_06. Exclusion reason: Not relevant outcome

Dinya, E., Csorba, J., Suli, A., & Grosz, Z. (2012). Behaviour profile of Hungarian adolescent outpatients with a dual diagnosis. *Research in Developmental Disabilities, 33*(5), 1574-1580. 10.1016/j.ridd.2012.03.001. Exclusion reason: No psychometric information

Doan, T., Ware, R., McPherson, L., van Dooren, K., Bain, C., Carrington, S., . . . Lennox, N. (2014). Psychotropic medication use in adolescents with intellectual disability living in the community. *Pharmacoepidemiology & Drug Safety, 23*(1), 69-76. 10.1002/pds.3484. Exclusion reason: No psychometric information

Dodd, H. F., & Porter, M. A. (2009). Psychopathology in Williams syndrome: The effect of individual differences across the life span. *Journal of Mental Health Research in Intellectual Disabilities, 2*(2), 89-109. 10.1080/19315860902725867. Exclusion reason: No psychometric information

Dossetor, D., White, D., & Whatson, L. (2011). Establishing a clinical framework for the mental health (MH) of children and adolescents (C&A) with intellectual disability (ID): Identifying factors that impede training and service development. *Journal of Paediatrics and Child Health, 2)*, 21. 10.1111/j.1440-1754.2011.02118.x. Exclusion reason: Conference abstract

Dovgan, K., Mazurek, M. O., & Hansen, J. (2019). Measurement invariance of the Child Behavior Checklist in children with autism spectrum disorder with and without intellectual disability: Follow-up study. *Research in Autism Spectrum Disorders, 58*, 19-29. 10.1016/j.rasd.2018.11.009. Exclusion reason: Wrong patient population

Doyle, A., Ostrander, R., Skare, S., Crosby, R. D., & August, G. J. (1997). Convergent and criterion-related validity of the Behavior Assessment System for Children-Parent Rating Scale. *Journal of Clinical Child Psychology, 26*(3), 276-284. 10.1207/s15374424jccp2603_6. Exclusion reason: No intellectual disability information

Dumont, E., Kroes, D., Korzilius, H., Didden, R., & Rojahn, J. (2014). Psychometric properties of a Dutch version of the Behavior Problems Inventory-01 (BPI-01). *Research in Developmental Disabilities, 35*(3), 603-610. 10.1016/j.ridd.2014.01.003. Exclusion reason: Results not reported separately for children and adolescents

Dykens, E. M., & Kasari, C. (1997). Maladaptive behavior in children with Prader-Willi syndrome, Down syndrome, and nonspecific mental retardation. *American Journal on Mental Retardation, 102*(3), 228-237. 10.1352/0895-8017(1997)102<0228:MBICWP>2.0.CO;2. Exclusion reason: No psychometric information

Einfeld, S., Tonge, B., Turner, G., Parmenter, T., & Smith, A. (1999). Longitudinal course of behavioural and emotional problems of young persons with Prader-Willi, Fragile X, Williams and Down syndromes. *Journal of Intellectual & Developmental Disability, 24*(4), 349-354. Exclusion reason: No psychometric information

Einfeld, S., & Tonge, J. (1996). Population prevalence of psychopathology in children and adolescents with intellectual disability: I. Rationale and methods. *Journal of Intellectual Disability Research, 40*(2), 91-98. 10.1111/j.1365-2788.1996.tb00610.x. Exclusion reason: Theoretical article/Comment

Einfeld, S. L., Piccinin, A. M., Mackinnon, A., Hofer, S. M., Taffe, J., Gray, K. M., . . . Tonge, B. J. (2006). Psychopathology in young people with intellectual disability. *JAMA: Journal of the American Medical Association, 296*(16), 1981-1989. 10.1001/jama.296.16.1981. Exclusion reason: No psychometric information

Einfeld, S. L., Smith, A., Durvasula, S., Florio, T., & Tonge, B. J. (1999). Behavior and emotional disturbance in Prader-Willi syndrome. *American Journal of Medical Genetics, 82*(2), 123-127. Exclusion reason: No psychometric information

Einfeld, S. L., & Tonge, B. (1999). Observations on the use of the ICD-10 Guide for Mental Retardation. *Journal of Intellectual Disability Research, 43*(5), 408-412. 10.1046/j.1365-2788.1999.043005408.x. Exclusion reason: No psychometric information

Einfeld, S. L., & Tonge, B. J. (1991). Psychometric and clinical assessment of psychopathology in developmentally disabled children. *Australia & New Zealand Journal of Developmental Disabilities, 17*(2), 147-154. Exclusion reason: Theoretical article/Comment

Einfeld, S. L., & Tonge, B. J. (1996). Population prevalence of psychopathology in children and adolescents with intellectual disability: II. Epidemiological findings. *Journal of Intellectual Disability Research, 40*(Pt 2), 99-109. Exclusion reason: No psychometric information

Einfeld, S. L., Tonge, B. J., & Florio, T. (1994). BEHAVIORAL AND EMOTIONAL DISTURBANCE IN FRAGILE-X-SYNDROME. *American Journal of Medical Genetics, 51*(4), 386-391. 10.1002/ajmg.1320510417. Exclusion reason: Duplicate reference

Einfeld, S. L., Tonge, B. J., & Florio, T. (1994). Behavioural and emotional disturbance in fragile X syndrome. *American Journal of Medical Genetics, 51*(4), 386-391. Exclusion reason: No psychometric information

Einfeld, S. L., Tonge, B. J., & Florio, T. (1997). Behavioral and emotional disturbance in individuals with Williams syndrome. *American Journal of Mental Retardation, 102*(1), 45-53. Exclusion reason: No psychometric information

Einfeld, S. L. T. B. J. (1995). Developmental Behavior Checklist. In.

Einfeld, S. L. T. B. J. (1995). Developmental Behavior Checklist--Teachers Version. In.

Eklund, H., Findon, J., Cadman, T., Hayward, H., Murphy, D., Asherson, P., . . . Xenitidis, K. (2018). Needs of adolescents and young adults with neurodevelopmental disorders: Comparisons of young people and parent perspectives. *Journal of Autism and Developmental Disorders, 48*(1), 83-91. 10.1007/s10803-017-3295-x. Exclusion reason: No intellectual disability information

Elander, J., & Rutter, M. (1996). Use and development of the Rutter parents' and teachers' scales. *International Journal of Methods in Psychiatric Research, 6*(2), 63-78. 10.1002/(sici)1234-988x(199607)6:2<63::Aid-mpr151>3.3.Co;2-m. Exclusion reason: Review

Elliott, S. N., & McKinnie, D. M. (1994). Relationships and differences among social skills, problem behaviors, and academic competence for mainstreamed learning-disabled and nonhandicapped students. *Canadian Journal of School Psychology, 10*(1), 1-14. 10.1177/082957359401000102. Exclusion reason: No intellectual disability information

Embregts, P., Didden, R., Huitink, C., & Schreuder, N. (2009). Contextual variables affecting aggressive behaviour in individuals with mild to borderline intellectual disabilities who live in a residential facility. *Journal of Intellectual Disability Research, 53*(3), 255-264. 10.1111/j.1365-2788.2008.01132.x. Exclusion reason: Results not reported separately for children and adolescents

Embregts, P. J. (2000). Reliability of the Child Behavior Checklist for the assessment of behavioral problems of children and youth with mild mental retardation. *Research in Developmental Disabilities, 21*(1), 31-41. 10.1016/S0891-4222%2899%2900028-1. Exclusion reason: Wrong patient population

Emerson, E. (2003). Prevalence of psychiatric disorders in children and adolescents with and without intellectual disability. *Journal of Intellectual Disability Research, 47*(1), 51-58. Exclusion reason: No psychometric information

Emerson, E. (2005). Use of the Strengths and Difficulties Questionnaire to access the mental health needs of children and adolescents with intellectual disabilities. *Journal of Intellectual & Developmental Disability, 30*(1), 14-23. Exclusion reason: Duplicate reference

Emerson, E., Einfeld, S., Stancliffe, R. J., Emerson, E., Einfeld, S., & Stancliffe, R. J. (2010). The mental health of young children with intellectual disabilities or borderline intellectual functioning. *Social Psychiatry & Psychiatric Epidemiology, 45*(5), 579-587. 10.1007/s00127-009-0100-y. Exclusion reason: No psychometric information

Emerson, E., Felce, D., & Stancliffe, R. J. (2013). Issues Concerning Self-Report Data and Population-Based Data Sets Involving People With Intellectual Disabilities. *Intellectual and Developmental Disabilities, 51*(5), 333-348. 10.1352/1934-9556-51.5.333. Exclusion reason: Theoretical article/Comment

Emerson, E., Kiernan, C., Alborz, A., Reeves, D., Mason, H., Swarbrick, R., . . . Hatton, C. (2001). The prevalence of challenging behaviors: A total population study. *Research in Developmental Disabilities, 22*(1), 77-93. 10.1016/S0891-4222%2800%2900061-5. Exclusion reason: No psychometric information

Emser, T. S., Mazzucchelli, T. G., Christiansen, H., & Sanders, M. R. (2016). Child Adjustment and Parent Efficacy Scale-Developmental Disability (CAPES-DD): First psychometric evaluation of a new child and parenting assessment tool for children with a developmental disability. *Research in Developmental Disabilities, 53-54*, 158-177. 10.1016/j.ridd.2015.09.006. Exclusion reason: Wrong patient population

Epstein, M. H. (1999). The development and validation of a scale to assess the emotional and behavioral strengths of children and adolescents. *Remedial and Special Education, 20*(5), 258-263. 10.1177/074193259902000501. Exclusion reason: Not relevant measurement tool

Epstein, M. H., & Cullinan, D. (1984). Behavior problems of mildly handicapped and normal adolescents. *Journal of Clinical Child Psychology, 13*(1), 33-37. 10.1080/15374418409533166. Exclusion reason: No psychometric information

Epstein, M. H., Cullinan, D., & Bursuck, W. D. (1985). Prevalence of behavior problems among learning disabled and nonhandicapped students. *Mental Retardation & Learning Disability Bulletin, 13*(1), 30-39. Exclusion reason: No psychometric information

Epstein, M. H., Cullinan, D., & Nieminen, G. (1984). Social behavior problems of learning disabled and normal girls. *Journal of Learning Disabilities, 17*(10), 609-611. 10.1177/002221948401701007. Exclusion reason: No intellectual disability information

Epstein, M. H., Cullinan, D., & Polloway, E. A. (1986). Patterns of maladjustment among mentally retarded children and youth. *American Journal of Mental Deficiency, 91*(2), 127-134. Exclusion reason: No psychometric information

Epstein, M. H., & et al. (1983). Behavior Problem Patterns among the Learning Disabled: Boys Aged 6-11. *Learning Disability Quarterly, 6*(3), 305-311. Exclusion reason: Wrong patient population

Esbensen, A., & Hoffman, E. (2017). Reliability of parent report measures of sleep in children with Down syndrome. *Journal of Intellectual Disability Research, 61*(3), 210-220. 10.1111/jir.12315. Exclusion reason: Not relevant measurement tool

Esbensen, A. J., Rojahn, J., Aman, M. G., & Ruedrich, S. (2003). Reliability and validity of an assessment instrument for anxiety, depression, and mood among individuals with mental retardation. *Journal of Autism and Developmental Disorders, 33*(6), 617-629. 10.1023/b:Jadd.0000005999.27178.55. Exclusion reason: Results not reported separately for children and adolescents

Esbensen, A. J., Seltzer, M. M., Greenberg, J. S., & Benson, B. A. (2005). Psychometric evaluation of a self-report measure of depression for individuals with mental retardation. *American Journal on Mental Retardation, 110*(6), 469-481. 10.1352/0895-8017(2005)110[469:Peoasm]2.0.Co;2. Exclusion reason: Results not reported separately for children and adolescents

Esteba-Castillo, S., Torrents-Rodas, D., Garcia-Alba, J., Ribas-Vidal, N., & Novell-Alsina, R. (2018). Translation and validation of the Spanish version of the Health of the Nation Outcome Scales for People with Learning Disabilities (HoNOS-LD). *Revista de Psiquiatria y Salud Mental, 11*(3), 141-150. 10.1016/j.rpsm.2016.11.002. Exclusion reason: Adult population

Evans, E., Mowat, D., Wilson, M., & Einfeld, S. (2016). Sleep disturbance in Mowat-Wilson syndrome. *American Journal of Medical Genetics. Part A, 170*(3), 654-660. 10.1002/ajmg.a.37502. Exclusion reason: Results not reported separately for children and adolescents

Excoffier, E., Vila, G., Taupiac, E., Mouren-Simeoni, M., & Bouvard, M. (2007). Dimensional approach of social behaviour deficits in children. Preliminary validation study of the French version of the Children's Social Behaviour Questionnaire (CSBQ). *L'Encephale: Revue de psychiatrie clinique biologique et therapeutique, 33*(4, Pt 1), 585-591. 10.1016/S0013-7006%2807%2992057-6. Exclusion reason: Wrong patient population

Ezell, J., Hogan, A., Fairchild, A., Hills, K., Klusek, J., Abbeduto, L., & Roberts, J. (2019). Prevalence and Predictors of Anxiety Disorders in Adolescent and Adult Males with Autism Spectrum Disorder and Fragile X Syndrome. *Journal of Autism and Developmental Disorders, 49*(3), 1131-1141. 10.1007/s10803-018-3804-6. Exclusion reason: Results not reported separately for children and adolescents

Farmer, C. A., & Aman, M. G. (2009). Development of the Children's Scale of Hostility and Aggression: Reactive/Proactive (C-SHARP). *Research in Developmental Disabilities, 30*(6), 1155-1167. 10.1016/j.ridd.2009.03.001. Exclusion reason: No intellectual disability information

Farmer, C. A., & Aman, M. G. (2010). Psychometric properties of the Children's Scale of Hostility and Aggression: Reactive/Proactive (C-SHARP). *Research in Developmental Disabilities, 31*(1), 270-280. 10.1016/j.ridd.2009.09.014. Exclusion reason: No intellectual disability information

Fee, V. E., Matson, J. L., Moore, L. A., & Benavidez, D. A. (1993). The differential validity of hperactivity/attention deficits and conduct problems among mentally retarded children. *Journal of Abnormal Child Psychology, 21*(1), 1-11. Exclusion reason: Duplicate reference

Fee, V. E., Matson, J. L., Moore, L. A., & Benavidez, D. A. (1993). The differential validity of hyperactivity/attention deficits and conduct problems among mentally retarded children. *Journal of Abnormal Child Psychology, 21*(1), 1-11. 10.1007/BF00910485. Exclusion reason: Wrong patient population

Feinstein, C., Kaminer, Y., Barrett, R. P., & Tylenda, B. (1988). The assessment of mood and affect in developmentally disabled children and adolescents: The Emotional Disorders Rating Scale. *Research in Developmental Disabilities, 9*(2), 109-121. 10.1016/0891-4222%2888%2990045-5. Exclusion reason: Results not reported separately for children and adolescents

Fertrin, K. Y., Goncalves, M. S., Saad, S. T. O., & Costa, F. F. (2002). Problem behavior in boys with fragile X syndrome. *American Journal of Medical Genetics, 108*(2), 105-116. 10.1002/ajmg.10216. Exclusion reason: No psychometric information

Fidan, T., Kirpinar, I., Oral, M., & Kocak, K. (2011). Is there a relationship between attention deficit/hyperactivity disorder and manic symptoms among children with mental retardation of unknown etiology? *Comprehensive Psychiatry, 52*(6), 644-649. 10.1016/j.comppsych.2010.11.007. Exclusion reason: No psychometric information

Fidler, D. J., Most, D. E., Booth-LaForce, C., & Kelly, J. F. (2006). Temperament and behaviour problems in young children with Down syndrome at 12, 30, and 45 months. *Down Syndrome: Research & Practice, 10*(1), 23-29. Exclusion reason: Mean age < 4 years

Findling, R. L., Aman, M. G., Eerdekens, M., Derivan, A., & Lyons, B. (2004). Long-term, open-label study of risperidone in children with severe disruptive behaviors and below-average IQ. *American Journal of Psychiatry, 161*(4), 677-684. Exclusion reason: Outcome: psychopharmacology

Fletcher, R. J., Havercamp, S. M., Ruedrich, S. L., Benson, B. A., Barnhill, L., Cooper, S. A., & Stavrakaki, C. (2009). Clinical usefulness of the Diagnostic Manual-Intellectual Disability for mental disorders in persons with intellectual disability: Results from a brief field survey. *The Journal of Clinical Psychiatry, 70*(7), 967-974. 10.4088/JCP.08m04429. Exclusion reason: Results not reported separately for children and adolescents

Floyd, E. M., Rayfield, A., Eyberg, S. M., & Riley, J. L., III. (2004). Psychometric Properties of the Sutter-Eyberg Student Behavior Inventory With Rural Middle School and High School Children. *Assessment, 11*(1), 64-72. 10.1177/1073191103260945. Exclusion reason: No intellectual disability information

Floyd, F. J., & Zmich, D. E. (1991). Marriage and the parenting partnership: Perceptions and interactions of parents with mentally retarded and typically developing children. *Child Development, 62*(6), 1434-1448. 10.2307/1130817. Exclusion reason: Not relevant measurement tool

Foley, K. R., Taffe, J., Bourke, J., Einfeld, S. L., Tonge, B. J., Trollor, J., & Leonard, H. (2016). Young People with Intellectual Disability Transitioning to Adulthood: Do Behaviour Trajectories Differ in Those with and without Down Syndrome? *PLoS ONE [Electronic Resource], 11*(7), e0157667. 10.1371/journal.pone.0157667. Exclusion reason: Not relevant outcome

Fombonne, E., Simmons, H., Ford, T., Meltzer, H., & Goodman, R. (2003). Prevalance of pervasive developmental disorders in the British nationalwide survey of child mental heath. *International Review of Psychiatry, 15*(1-2), 158-165. 10.1080/0954026021000046119. Exclusion reason: No psychometric information

Fombonne, E., Simmons, H., Ford, T., Meltzer, H., & Goodman, R. (2003). Prevalence of pervasive developmental disorders in the British nationwide survey of child mental health. *International Review of Psychiatry, 15*(1-2), 158-165. Exclusion reason: Duplicate reference

Forster, S., Gray, K. M., Taffe, J., Einfeld, S. L., & Tonge, B. J. (2011). Behavioural and emotional problems in people with severe and profound intellectual disability. *Journal of Intellectual Disability Research, 55*, 190-198. 10.1111/j.1365-2788.2010.01373.x. Exclusion reason: No psychometric information

Fox, R. A., Keller, K. M., Grede, P. L., & Bartosz, A. M. (2007). A mental health clinic for toddlers with developmental delays and behavior problems. *Research in Developmental Disabilities, 28*(2), 119-129. 10.1016/j.ridd.2006.02.001. Exclusion reason: Mean age < 4 years

Franzese, A., Mozzillo, E., Zito, E., Ferrentino, R. I., De Nitto, E., Idelson, P. I., . . . Fattorusso, V. (2013). Prader-Willi syndrome: Influence of the genotype on the mental performances. *Hormone research in paediatrics, 1)*, 322. Exclusion reason: Conference abstract

Fraser, W. I., Leudar, I., Gray, J., & Campbell, I. (1986). Psychiatric and behaviour disturbance in mental handicap. *Journal of Mental Deficiency Research, 30*(Pt 1), 49-57. Exclusion reason: Adult population

Freeman, B. J., Ritvo, E. R., Tonick, I., Guthrie, D., & Schroth, P. (1981). Behavior observation system for autism: analysis of behaviors among autistic, mentally retarded, and normal children. *Psychological Reports, 49*(1), 199-208. Exclusion reason: Not relevant measurement tool

Freeman, K., Williams, T. I., Farran, E., & Brown, J. (2010). Williams Syndrome: The extent of agreement between parent and self report of psychological. *The European Journal of Psychiatry, 24*(3), 167-175. 10.4321/S0213-61632010000300005. Exclusion reason: Results not reported separately for children and adolescents

Freeman, K. A., Walker, M., & Kaufman, J. (2007). Psychometric properties of the Questions About Behavioral Function Scale in a child sample. *American Journal on Mental Retardation, 112*(2), 122-129. 10.1352/0895-8017%282007%29112%5B122:PPOTQA%5D2.0.CO;2. Exclusion reason: Wrong patient population

Friedlander, R. I., & Donnelly, T. (2004). Early-onset psychosis in youth with intellectual disability. *Journal of Intellectual Disability Research, 48*(6), 540-547. 10.1111/j.1365-2788.2004.00622.x. Exclusion reason: Adult population

Friedman, D. H. (1998). Social skills and problem behaviors of adolescents with learning disabilities with and without attention/impulsivity problems: A comparison of teacher and student perceptions. *Dissertation Abstracts International Section A: Humanities and Social Sciences, 59*(5-A), 1525. Exclusion reason: Not relevant outcome

Frolli, A., Piscopo, S., & Conson, M. (2015). Developmental changes in cognitive and behavioural functioning of adolescents with fragile-X syndrome. *Journal of Intellectual Disability Research, 59*(7), 613-621. 10.1111/jir.12165. Exclusion reason: No psychometric information

Gabis, L. V., Shilon-Hadass, A., Misgav-Tzuberi, N., Sofrin, R., & Shefer, S. (2010). Evaluation of ability and comorbidity in children with cerebral palsy. *Annals of Neurology, 14)*, S100. 10.1002/ana.22199. Exclusion reason: Conference abstract

Gabriels, R. L., Cuccaro, M. L., Hill, D. E., Ivers, B. J., & Goldson, E. (2005). Repetitive behaviors in autism: Relationships with associated clinical features. *Research in Developmental Disabilities, 26*(2), 169-181. 10.1016/j.ridd.2004.05.003. Exclusion reason: Wrong patient population

Gadow, K. D., DeVincent, C. J., Pomeroy, J., & Azizian, A. (2004). Psychiatric Symptoms in Preschool Children with PDD and Clinic and Comparison Samples. *Journal of Autism and Developmental Disorders, 34*(4), 379-393. 10.1023/B:JADD.0000037415.21458.93. Exclusion reason: Wrong patient population

Gadow, K. D., Devincent, C. J., Pomeroy, J., & Azizian, A. (2005). Comparison of DSM-IV symptoms in elementary school-age children with PDD versus clinic and community samples. *Autism, 9*(4), 392-415. 10.1177/1362361305056079. Exclusion reason: No intellectual disability information

Gadow, K. D., Sprafkin, J., & Nolan, E. E. (2001). DSM-IV symptoms in community and clinic preschool children. *Journal of the American Academy of Child & Adolescent Psychiatry, 40*(12), 1383-1392. 10.1097/00004583-200112000-00008. Exclusion reason: No intellectual disability information

Gagliardi, C., Martelli, S., Tavano, A., & Borgatti, R. (2011). Behavioural features of Italian infants and young adults with Williams-Beuren syndrome. *Journal of Intellectual Disability Research, 55*(2), 121-131. 10.1111/j.1365-2788.2010.01376.x. Exclusion reason: Results not reported separately for children and adolescents

Gajar, A. H., & Hale, R. L. (1982). Factor analysis of the Quay-Peterson Behavior Problem Check-list across racially different exceptional children. *The Journal of Psychology: Interdisciplinary and Applied, 112*(2), 287-293. 10.1080/00223980.1982.9915386. Exclusion reason: No intellectual disability information

Galera, C., Taupiac, E., Fraisse, S., Naudion, S., Toussaint, E., Rooryck-Thambo, C., . . . Bouvard, M. P. (2009). Socio-behavioral characteristics of children with Rubinstein-Taybi syndrome. *Journal of Autism & Developmental Disorders, 39*(9), 1252-1260. 10.1007/s10803-009-0733-4. Exclusion reason: No intellectual disability information

Gallegos, J., Langley, A., & Villegas, D. (2012). Anxiety, Depression, and Coping Skills among Mexican School Children: A Comparison of Students with and without Learning Disabilities. *Learning Disability Quarterly, 35*(1), 54-61. Exclusion reason: Wrong patient population

Gallo, F. J. (2009). Executive functions in young children with Williams syndrome. *Dissertation Abstracts International: Section B: The Sciences and Engineering, 70*(6-B), 3808. Exclusion reason: Not relevant outcome

Gardiner, E., Miller, A. R., & Lach, L. M. (2020). Topography of behavior problems among children with neurodevelopmental conditions: Profile differences and overlaps. *Child: Care, Health and Development, 46*(1), 149-153. 10.1111/cch.12720. Exclusion reason: No psychometric information

Gardner, L. M., Campbell, J. M., Bush, A. J., & Murphy, L. (2018). Comparing Behavioral Profiles for Autism Spectrum Disorders and Intellectual Disabilities Using the BASC-2 Parent Rating Scales--Preschool Form. *Journal of Psychoeducational Assessment, 36*(6), 535-551. Exclusion reason: Adult population

Garner, K. S. (2015). Comparison of the behavior of individuals with early-onset morbid obesity and prader-willi syndrome using the behavior assessment system for children, second edition. *Dissertation Abstracts International: Section B: The Sciences and Engineering, 75*(7-B(E)), No Pagination Specified. Exclusion reason: Wrong patient population

Gath, A., & Gumley, D. (1986). Behaviour problems in retarded children with special reference to Down's Syndrome. *The British Journal of Psychiatry, 149*, 156-161. 10.1192/bjp.149.2.156. Exclusion reason: No psychometric information

Gerber, M. L. (2001). Prader-Wwilli syndrome: Emotional and behavioral characteristics of children and adolescents, A comparative study. *Dissertation Abstracts International: Section B: The Sciences and Engineering, 62*(1-B), 547. Exclusion reason: No psychometric information

Giarelli, E., Clarke, D. L., Catching, C., & Ratcliffe, S. J. (2009). Developmental disabilities and behavioral problems among school children in the Western Cape of South Africa. *Research in Developmental Disabilities, 30*(6), 1297-1305. 10.1016/j.ridd.2009.05.006. Exclusion reason: No intellectual disability information

Gillberg, C. (1987). Associated neuropsychiatric problems in Swedish school children with mild mental retardation. *Upsala Journal of Medical Sciences - Supplement, 44*, 111-114. Exclusion reason: Duplicate reference

Gillberg, C. (1987). Associated neuropsychiatric problems in Swedish school children with mild mental retardation. *Upsala journal of medical sciences, Supplement. 44*, 111-114. Exclusion reason: Not relevant outcome

Giltaij, H. P., Sterkenburg, P. S., & Schuengel, C. (2016). Adaptive behaviour, comorbid psychiatric symptoms, and attachment disorders. *Advances in Mental Health & Intellectual Disabilities, 10*(1), 82-91. 10.1108/AMHID-07-2015-0035. Exclusion reason: Printed books;

Gimpel, G. A. (1996). Construct validation of the Devereux Behavior Rating Scale--School Form and the Devereux Scales of Mental Disorders. *Dissertation Abstracts International Section A: Humanities and Social Sciences, 56*(8-A), 3056. Exclusion reason: Duplicate reference

Gimpel, G. A., & Nagle, R. J. (1999). Psychometric properties of the Devereux Scales of Mental Disorders. *Journal of Psychoeducational Assessment, 17*(2), 127-144. 10.1177/073428299901700203. Exclusion reason: No intellectual disability information

Gjevik, E., Eldevik, S., Fjaeran-Granum, T., & Sponheim, E. (2011). Kiddie-SADS reveals high rates of DSM-IV disorders in children and adolescents with autism spectrum disorders. *Journal of Autism and Developmental Disorders, 41*(6), 761-769. 10.1007/s10803-010-1095-7. Exclusion reason: Wrong patient population

Gjevik, E., Sandstad, B., Andreassen, O. A., Myhre, A. M., & Sponheim, E. (2015). Exploring the agreement between questionnaire information and DSM-IV diagnoses of comorbid psychopathology in children with autism spectrum disorders. *Autism, 19*(4), 433-442. 10.1177/1362361314526003. Exclusion reason: Wrong patient population

Glazebrook, C., Hollis, C., Heussler, H., Goodman, R., & Coates, L. (2003). Detecting emotional and behavioural problems in paediatric clinics. *Child: Care, Health & Development, 29*(2), 141-149. Exclusion reason: Wrong patient population

Glenn, S., Cunningham, C., Nananidou, A., Prasher, V., & Glenholmes, P. (2015). Routinised and compulsive-like behaviours in individuals with Down syndrome. *Journal of Intellectual Disability Research, 59*(11), 1061-1070. 10.1111/jir.12199. Exclusion reason: Adult population

Gobrial, E., & Raghavan, R. (2012). Prevalence of anxiety disorder in children and young people with intellectual disabilities and autism. *Advances in Mental Health and Intellectual Disabilities, 6*(3), 130-140. 10.1108/20441281211227193. Exclusion reason: No psychometric information

Goker, Z., Guney, E., Dinc, G., Hekim, O., & Uneri, O. (2014). The clinical and demographic features of cases with pervasive developmental disorder evaluated in a training and research hospital. *Cocuk ve Genclik Ruh Sagligi Dergisi, 21*(2), 95-104. Exclusion reason: Article in foreign language/not accessible language

Goldin, R. L., Matson, J. L., & Cervantes, P. E. (2014). The effect of intellectual disability on the presence of comorbid symptoms in children and adolescents with autism spectrum disorder. *Research in Autism Spectrum Disorders, 8*(11), 1552-1556. 10.1016/j.rasd.2014.08.006. Exclusion reason: No psychometric information

Goodman, R., Yude, C., Richards, H., & Taylor, E. (1996). Rating child psychiatric caseness from detailed case histories. *Child Psychology & Psychiatry & Allied Disciplines, 37*(4), 369-379. 10.1111/j.1469-7610.1996.tb01418.x. Exclusion reason: No psychometric information

Goretti, B., Ghezzi, A., Portaccio, E., Lori, S., Zipoli, V., Razzolini, L., . . . Amato, M. P. (2010). Fatigue and psychosocial issues in children and adolescents with multiple sclerosis. *Multiple Sclerosis, 1)*, S276. 10.1177/1352458510383204. Exclusion reason: Wrong patient population

Gothelf, D. (2015). Neurodevelopmental risk factors for psychosis in 22q11.2 deletion syndrome compared to williams syndrome. *Biological Psychiatry, 1)*, 150S. Exclusion reason: Conference abstract

Gothelf, D., Feinstein, C., Thompson, T., Gu, E., Penniman, L., Van Stone, E., . . . Reiss, A. L. (2007). Risk factors for the emergence of psychotic disorders in adolescents with 22q11.2 deletion syndrome. *American Journal of Psychiatry, 164*(4), 663-669. Exclusion reason: Wrong patient population

Gothelf, D., Goraly, O., Avni, S., Stawski, M., Hartmann, I., Basel-Vanagaite, L., & Apter, A. (2008). Psychiatric morbidity with focus on obsessive-compulsive disorder in an Israeli cohort of adolescents with mild to moderate mental retardation. *Journal of Neural Transmission, 115*(6), 929-936. 10.1007/s00702-008-0037-4. Exclusion reason: No psychometric information

Gothelf, D., & State, M. (2018). 22Q11 Deletion Syndrome as a Genetic High-Risk Model for Developmental Neuropsychiatric Disorders: From Circuitry to Treatments. *Journal of the American Academy of Child and Adolescent Psychiatry, 57 (10 Supplement)*, S283. 10.1016/j.jaac.2018.07.668. Exclusion reason: Conference abstract

Gray, K., Keating, C., Taffe, J., Brereton, A., Einfeld, S., & Tonge, B. (2012). Trajectory of Behavior and Emotional Problems in Autism. *Ajidd-American Journal on Intellectual and Developmental Disabilities, 117*(2), 121-133. 10.1352/1944-7588-117-2.121. Exclusion reason: No psychometric information

Gray, K., Melvin, G., & McGivern, L. (2016). How I'm Feeling: The development of a new self-report measure for adolescents with intellectual disabilities. *Journal of Intellectual Disability Research, 60 (7-8)*, 673. Exclusion reason: Conference abstract

Gray, K. M., Piccinin, A. M., Hofer, S. M., Mackinnon, A., Bontempo, D. E., Einfeld, S. L., . . . Tonge, B. J. (2011). The longitudinal relationship between behavior and emotional disturbance in young people with intellectual disability and maternal mental health. *Research in Developmental Disabilities, 32*(3), 1194-1204. 10.1016/j.ridd.2010.12.044. Exclusion reason: No psychometric information

Green, T., Avda, S., Dotan, I., Zarchi, O., Basel-Vanagaite, L., Zalsman, G., . . . Gothelf, D. (2012). Phenotypic psychiatric characterization of children with Williams syndrome and response of those with ADHD to methylphenidate treatment. *American Journal of Medical Genetics Part B-Neuropsychiatric Genetics, 159B*(1), 13-20. 10.1002/ajmg.b.31247. Exclusion reason: No psychometric information

Green, T., Gothelf, D., Glaser, B., Debbane, M., Frisch, A., Kotler, M., . . . Eliez, S. (2009). Psychiatric disorders and intellectual functioning throughout development in velocardiofacial (22q11.2 deletion) syndrome. *Journal of the American Academy of Child & Adolescent Psychiatry, 48*(11), 1060-1068. 10.1097/CHI.0b013e3181b76683. Exclusion reason: Wrong patient population

Green, V. A., O'Reilly, M., Itchon, J., & Sigafoos, J. (2005). Persistence of early emerging aberrant behavior in children with developmental disabilities. *Research in Developmental Disabilities, 26*(1), 47-55. 10.1016/j.ridd.2004.07.003. Exclusion reason: No intellectual disability information

Guran, T., Arman, A., Akcay, T., Kayan, E., Atay, Z., Turan, S., & Bereket, A. (2011). Cognitive and psychosocial development in children with familial hypomagnesaemia. *Magnesium Research, 24*(1), 7-12. 10.1684/mrh.2011.0278. Exclusion reason: Wrong patient population

Hall, D., Kaytser, V., Ouyang, B., & Berry-Kravis, E. (2012). Motor stereotypies in Fragile X syndrome. *Movement Disorders, 1)*, S437-S438. 10.1002/mds.25051. Exclusion reason: Conference abstract

Hall, S., Barnett, R., & Hustyi, K. (2016). Problem behaviour in adolescent boys with fragile X syndrome: Relative prevalence, frequency and severity. *Journal of Intellectual Disability Research, 60*(12), 1189-1199. 10.1111/jir.12341. Exclusion reason: Not relevant measurement tool

Halvorsen, M., Aman, M. G., Mathiassen, B., Brondbo, P. H., Steinsvik, O. O., & Martinussen, M. (2019). Psychometric properties of the norwegian aberrant behavior checklist and diagnostic relationships in a neuro-pediatric sample. *Journal of Mental Health Research in Intellectual Disabilities*, No Pagination Specified. 10.1080/19315864.2019.1630872. Exclusion reason: Wrong patient population

Halvorsen, M., Myrbakk, E., Mathiassen, B., Steinsvik, O., & Martinussen, M. (2015). The aberrant behavior checklist: Psychometric properties in a neuro-paediatric sample. *European Child and Adolescent Psychiatry, 1)*, S168-S169. 10.1007/s00787-015-0714-4. Exclusion reason: Conference abstract

Handwerk, M. L., & Marshall, R. M. (1998). Behavioral and emotional problems of students with learning disabilities, serious emotional disturbance, or both conditions. *Journal of Learning Disabilities, 31*(4), 327-338. 10.1177/002221949803100402. Exclusion reason: Wrong patient population

Hanson, E. M., Sideridis, G., Jackson, F. I., Porche, K., Campe, K. L., & Huntington, N. (2016). Behavior and Sensory Interests Questionnaire: Validation in a sample of children with autism spectrum disorder and other developmental disability. *Research in Developmental Disabilities, 48*, 160-175. 10.1016/j.ridd.2015.09.004. Exclusion reason: Not relevant outcome

Hanssen-Bauer, K., Heyerdahl, S., & Eriksson, A.-S. (2007). Mental health problems in children and adolescents referred to a national epilepsy center. *Epilepsy & Behavior, 10*(2), 255-262. 10.1016/j.yebeh.2006.11.011. Exclusion reason: Wrong patient population

Hardan, A., & Sahl, R. (1997). Psychopathology in children and adolescents with developmental disorders. *Research in Developmental Disabilities, 18*(5), 369-382. Exclusion reason: Not relevant measurement tool

Harris, J. C., King, S. L., Reifler, J. P., & Rosenberg, L. A. (1984). Emotional and learning disorders in 6-12-year-old boys attending special schools. *Journal of the American Academy of Child Psychiatry, 23*(4), 431-437. 10.1016/S0002-7138%2809%2960321-6. Exclusion reason: No intellectual disability information

Hartley, S., Sikora, D., & McCoy, R. (2008). Prevalence and risk factors of maladaptive behaviour in young children with Autistic Disorder. *Journal of Intellectual Disability Research, 52*(10), 819-829. 10.1111/j.1365-2788.2008.01065.x. Exclusion reason: Mean age < 4 years

Hassiotis, A., & Barron, D. A. (2007). Mental health, learning disabilities and adolescence: A developmental perspective. *Advances in Mental Health and Learning Disabilities, 1*(3), 32-39. 10.1108/17530180200700029. Exclusion reason: No psychometric information

Hassiotis, A., Serfaty, M., Azam, K., Strydom, A., Blizard, R., Romeo, R., . . . King, M. (2013). Manualised Individual Cognitive Behavioural Therapy for mood disorders in people with mild to moderate intellectual disability: a feasibility randomised controlled trial. *Journal of Affective Disorders, 151*(1), 186-195. 10.1016/j.jad.2013.05.076. Exclusion reason: Adult population

Hattier, M. A., Matson, J. L., Belva, B., & Kozlowski, A. (2012). The effects of diagnostic group and gender on challenging behaviors in infants and toddlers with cerebral palsy, Down syndrome or seizures. *Research in Developmental Disabilities, 33*(1), 258-264. 10.1016/j.ridd.2011.09.014. Exclusion reason: Mean age < 4 years

Hattier, M. A., Matson, J. L., Belva, B. C., & Horovitz, M. (2011). The occurrence of challenging behaviours in children with autism spectrum disorders and atypical development. *Developmental Neurorehabilitation, 14*(4), 221-229. 10.3109/17518423.2011.573836. Exclusion reason: Mean age < 4 years

Hatton, C., & Emerson, E. (2019). Mental health and people with intellectual disabilities in england: What does secondary analysis of national cohort surveys tell us? *Journal of Intellectual Disability Research, 63 (7)*, 681. 10.1111/jir.12653. Exclusion reason: Conference abstract

Hatton, C., Emerson, E., Robertson, J., & Baines, S. (2018). The mental health of adolescents with and without mild/moderate intellectual disabilities in England: Secondary analysis of a longitudinal cohort study. *Journal of Applied Research in Intellectual Disabilities, 31*(5), 768-777. 10.1111/jar.12428. Exclusion reason: No psychometric information

Havercamp, S., & Reiss, S. (1996). Composite versus multiple-rating scales in the assessment of psychopathology in people with mental retardation. *Journal of Intellectual Disability Research, 40*(2), 167-179. 10.1111/j.1365-2788.1996.tb00619.x. Exclusion reason: Results not reported separately for children and adolescents

Havercamp, S. M., & Reiss, S. (1997). The Reiss Screen for Maladaptive Behavior: Confirmatory factor analysis. *Behaviour Research and Therapy, 35*(10), 967-971. 10.1016/S0005-7967%2897%2900043-0. Exclusion reason: Results not reported separately for children and adolescents

Heikura, U., Ebeling, H., Rodriguez, A., Nordstrom, T., & Taanila, A. (2011). Leisure time activities and behavioral/emotional problems in adolescents with mild cognitive limitations. *European Child and Adolescent Psychiatry, 1)*, S98-S99. 10.1007/s00787-011-0181-5. Exclusion reason: Conference abstract

Hellings, J. A., Nickel, E. J., Weckbaugh, M., McCarter, K., Mosier, M., & Schroeder, S. R. (2005). The overt aggression scale for rating aggression in outpatient youth with autistic disorder: Preliminary findings. *Journal of Neuropsychiatry and Clinical Neurosciences, 17*(1), 29-35. 10.1176/jnp.17.1.29. Exclusion reason: Not relevant measurement tool

Hellings, J. A., Zarcone, J. R., Reese, R. M., Valdovinos, M. G., Marquis, J. G., Fleming, K. K., & Schroeder, S. R. (2006). A crossover study of risperidone in children, adolescents and adults with mental retardation. *Journal of Autism & Developmental Disorders, 36*(3), 401-411. Exclusion reason: Outcome: psychopharmacology

Hepburn, S. L., & Maclean, W. E. (2009). Maladaptive and repetitive behaviors in children with Down syndrome and autism spectrum disorders: Implications for screening. *Journal of Mental Health Research in Intellectual Disabilities, 2*(2), 67-88. 10.1080/19315860802627627. Exclusion reason: No psychometric information

Herring, S., Gray, K., Taffe, J., Tonge, B., Sweeney, D., & Einfeld, S. (2006). Behaviour and emotional problems in toddlers with pervasive developmental disorders and developmental delay: associations with parental mental health and family functioning. *Journal of Intellectual Disability Research, 50*(Pt 12), 874-882. Exclusion reason: Mean age < 4 years

Hill, J., & Furniss, F. (2006). Patterns of Emotional and Behavioural Disturbance Associated with Autistic Traits in Young People with Severe Intellectual Disabilities and Challenging Behaviours. *Research in Developmental Disabilities: A Multidisciplinary Journal, 27*(5), 517-528. Exclusion reason: Results not reported separately for children and adolescents

Hill, J., Powlitch, S., & Furniss, F. (2008). Convergent validity of the aberrant behavior checklist and behavior problems inventory with people with complex needs. *Research in Developmental Disabilities, 29*(1), 45-60. 10.1016/j.ridd.2006.10.002. Exclusion reason: Results not reported separately for children and adolescents

Hirota, T., Deserno, M., & McElroy, E. The Network Structure of Irritability and Aggression in Individuals with Autism Spectrum Disorder. *Journal of Autism and Developmental Disorders*. 10.1007/s10803-019-04354-w. Exclusion reason: No psychometric information

Hoch, J. D., & Youssef, A. M. (2019). Predictors of trauma exposure and trauma diagnoses for children with autism and developmental disorders served in a community mental health clinic. *Journal of Autism and Developmental Disorders*, No Pagination Specified. 10.1007/s10803-019-04331-3. Exclusion reason: No psychometric information

Hodapp, R. M., Fidler, D., & Smith, A. (1998). Stress and coping in families of children with Smith-Magenis syndrome. *Journal of Intellectual Disability Research, 42*(5), 331-340. 10.1046/j.1365-2788.1998.00148.x. Exclusion reason: No psychometric information

Hofer, S. M., Gray, K. M., Piccinin, A. M., Mackinnon, A., Bontempo, D. E., Einfeld, S. L., . . . Tonge, B. J. (2009). Correlated and coupled within-person change in emotional and behavioral disturbance in individuals with intellectual disability. *American Journal on Intellectual & Developmental Disabilities, 114*(5), 307-321. Exclusion reason: No psychometric information

Holland, J. E., Cassidy, A. R., Stopp, C., White, M. T., Bellinger, D. C., Rivkin, M. J., . . . DeMaso, D. R. (2017). Psychiatric Disorders and Function in Adolescents with Tetralogy of Fallot. *Journal of Pediatrics, 187*, 165-173. 10.1016/j.jpeds.2017.04.048. Exclusion reason: No psychometric information

Holmes, N., Shah, A., & Wing, L. (1982). The Disability Assessment Schedule: A brief screening device for use with the mentally retarded. *Psychological Medicine, 12*(4), 879-890. 10.1017/S0033291700049175. Exclusion reason: Results not reported separately for children and adolescents

Howland, R. H. (1995). Psychopathology and mental retardation. *American Journal of Psychiatry, 152*(12), 1837-1838. Exclusion reason: Theoretical article/Comment

Howlin, P., Warner, G., & Moss, J. (2016). The need for caution in using standardised autism measures with children with genetic conditions such as Down syndrome. *Journal of Intellectual Disability Research, 60 (7-8)*, 684. Exclusion reason: Conference abstract

Hustyi, K. M., Hall, S. S., Jo, B., Lightbody, A. A., & Reiss, A. L. (2014). Longitudinal trajectories of aberrant behavior in fragile X syndrome. *Research in Developmental Disabilities, 35*(11), 2691-2701. 10.1016/j.ridd.2014.07.003. Exclusion reason: No intellectual disability information

Hutton, J. B., & Roberts, T. (1983). Factor structure of problem behavior for mildly handicapped and non-handicapped students. *Psychological Reports, 52*(3), 703-707. Exclusion reason: No intellectual disability information

Hutzelmeyer-Nickels, A., & Noterdaeme, M. (2007). Usefulness of the Child Behavior Checklist in the assessment of preschool children with developmental problems. *Praxis der Kinderpsychologie und Kinderpsychiatrie, 56*(7), 573-588. 10.13109/prkk.2007.56.7.573. Exclusion reason: Duplicate reference

Hutzelmeyer-Nickels, A., & Noterdaeme, M. (2007). Usefulness of the Child Behavior Checklist in the assessment of preschool children with developmental problems. [German]. *Praxis der Kinderpsychologie und Kinderpsychiatrie, 56*(7), 573-588. Exclusion reason: Article in foreign language/not accessible language

Inada, N., Ito, H., Yasunaga, K., Kuroda, M., Iwanaga, R., Hagiwara, T., . . . Tsujii, M. (2015). Psychometric properties of the Repetitive Behavior Scale-Revised for individuals with autism spectrum disorder in Japan. *Research in Autism Spectrum Disorders, 15-16*, 60-68. 10.1016/j.rasd.2015.01.002. Exclusion reason: Wrong patient population

Infante Toscano, L., & Rodriguez Sacristan, J. (1998). Maladapted behavior in the mentally retarded: A cooperative study of Reiss's Screen and the Bouras Subscale of Behavioral Problems. *Anales de Psiquiatria, 14*(4), 133-146. Exclusion reason: Article in foreign language/not accessible language

Inoue, M., Inada, N., Gomi, Y., Aita, C., & Shiga, T. (2021). Reliability and validity of the japanese version of the behavior problem inventory-short form. *Brain & Development*, No Pagination Specified. 10.1016/j.braindev.2021.01.007. Exclusion reason: Results not reported separately for children and adolescents

Jamiolkowski, D., Kolker, S., Glahn, E. M., Baric, I., Zeman, J., Baumgartner, M. R., . . . consortium, E. I. (2016). Behavioural and emotional problems, intellectual impairment and health-related quality of life in patients with organic acidurias and urea cycle disorders. *Journal of Inherited Metabolic Disease, 39*(2), 231-241. 10.1007/s10545-015-9887-8. Exclusion reason: Not relevant measurement tool

Jansen, P. W., Duijff, S. N., Beemer, F. A., Vorstman, J. A., Klaassen, P. W., Morcus, M. E., & Heineman-de Boer, J. A. (2007). Behavioral problems in relation to intelligence in children with 22q11.2 deletion syndrome: a matched control study. *American Journal of Medical Genetics. Part A, 143A*(6), 574-580. Exclusion reason: No intellectual disability information

Jansen, P. W., Duijff, S. N., Beemer, F. A., Vorstman, J. A. S., Klaassen, P. W. J., Morcus, M. E. J., & Heineman-De Boer, J. A. (2007). Behavioral problems in relation to intelligence in children with 22q11.2 deletion syndrome: A matched control study. *American Journal of Medical Genetics, Part A, 143*(6), 574-580. 10.1002/ajmg.a.31623. Exclusion reason: Wrong patient population

Jeong, B., Yoo, E., Jung, M., Kang, D., Park, S., & Park, S. H. (2013). Validity and reliability of the Korean version of the Behaviour Problems Inventory. *Journal of Applied Research in Intellectual Disabilities, 26*(6), 578-590. Exclusion reason: Not relevant outcome

Jesmin, A., Mullick, M. S. I., Rahman, K. M., & Muntasir, M. M. (2016). Psychiatric Disorders in Children and Adolescents Attending Pediatric Out Patient Departments of Tertiary Hospitals. *Oman Medical Journal, 31*(4), 258-262. 10.5001/omj.2016.51. Exclusion reason: No intellectual disability information

Ji, N., Capone, G., & Kaufmann, W. (2011). Autism spectrum disorder in Down syndrome: Cluster analysis of Aberrant Behaviour Checklist data supports diagnosis. *Journal of Intellectual Disability Research, 55*(11), 1064-1077. 10.1111/j.1365-2788.2011.01465.x. Exclusion reason: Not relevant outcome

Johnels, J. A., Bostrom, P., & Broberg, M. (2014). Self-reported mental well-being in adolescents with intellectual and developmental disabilities: an initial psychometric evaluation of the WellSEQ. *Journal of Applied Research in Intellectual Disabilities, 27*(4), 313-313. Exclusion reason: Conference abstract

Johnson, C. R., Handen, B. L., Lubetsky, M. J., & Sacco, K. A. (1995). Affective disorders in hospitalized children and adolescents with mental retardation: a retrospective study. *Research in Developmental Disabilities, 16*(3), 221-231. Exclusion reason: No psychometric information

Johnson, K. (1999). Reliability and comorbidity measures of repetitive movement disorders in children and adolescents with severe mental retardation. *Dissertation Abstracts International: Section B: The Sciences and Engineering, 59*(9-B), 5066. Exclusion reason: Unable to obtain in full text

Johnson, S., Hollis, C., Marlow, N., Simms, V., & Wolke, D. (2014). Screening for childhood mental health disorders using the Strengths and Difficulties Questionnaire: The validity of multi-informant reports. *Developmental Medicine & Child Neurology, 56*(5), 453-459. 10.1111/dmcn.12360. Exclusion reason: No intellectual disability information

Johnstone, E. C., Owens, D. G. C., Hoare, P., Gaur, S., Spencer, M. D., Harris, J., . . . Muir, W. J. (2007). Schizotypal cognitions as a predictor of psychopathology in adolescents with mild intellectual impairment. *British Journal of Psychiatry, 191*, 484-492. 10.1192/bjp.bp.106.033514. Exclusion reason: No psychometric information

Jones, J., Siddarth, P., Gurbani, S., & Caplan, R. (2009). A comprehensive assessment of children with epilepsy and suicidality. *Epilepsia, 11)*, 497. 10.1111/j.1528-1167.2009.02377.x. Exclusion reason: Conference abstract

Jones, J. E., Watson, R., Sheth, R., Caplan, R., Koehn, M., Seidenberg, M., & Hermann, B. (2007). Psychiatric comorbidity in children with new onset epilepsy. *Developmental Medicine & Child Neurology, 49*(7), 493-497. Exclusion reason: Wrong patient population

Kaat, A. J., Lecavalier, L., & Aman, M. G. (2014). Validity of the Aberrant Behavior Checklist in Children with Autism Spectrum Disorder. Journal of Autism and Developmental Disorders, 44(5), 1103-1116. 10.1007/s10803-013-1970-0. Exclusion reason: No intellectual disability information.

Kaminer, Y., Feinstein, C., & Barrett, R. P. (1986). A relationship between pervasive developmental disorders and affective disorders? *Journal of the American Academy of Child Psychiatry, 25*(3), 434-435. 10.1016/S0002-7138%2809%2960272-7. Exclusion reason: Theoretical article/Comment

Kamio, Y., & Ishisaka, Y. (2002). Psychiatric Comorbidity in Children and Adolescents with Autism and Mental Retardatioon. *Japanese Journal of Child and Adolescent Psychiatry, 43*(3), 260-279. Exclusion reason: Not relevant outcome

Kapur, M., Reddy, M., Uma, H., Padma, B., & Shahul, M. (2000). Dual diagnoses in the intellectually disabled children and adolescents. *NIMHANS Journal, 18*(1-2), 19-32. Exclusion reason: No psychometric information

Karabekiroglu, K., & Aman, M. G. (2009). Validity of the aberrant behavior checklist in a clinical sample of toddlers. *Child Psychiatry & Human Development, 40*(1), 99-110. 10.1007/s10578-008-0108-7. Exclusion reason: Mean age < 4 years

Keenan, N. (2012). Parent-rated strengths of children and adolescents with Down syndrome. *Dissertation Abstracts International: Section B: The Sciences and Engineering, 73*(6-B), 3953. Exclusion reason: Not relevant outcome

Keith, L. K. (1997). Construction and concurrent and contrasted groups validation of the Clinical Assessment of Behavior Scale: Parent form. *Dissertation Abstracts International Section A: Humanities and Social Sciences, 57*(12-A), 5049. Exclusion reason: No intellectual disability information

Kellett, S., Beail, N., Newman, D. W., & Frankish, P. (2003). Utility of the Brief Symptom Inventory in the Assessment of Psychological Distress. *Journal of Applied Research in Intellectual Disabilities, 16*(2), 127-134. 10.1046/j.1468-3148.2003.00152.x. Exclusion reason: Results not reported separately for children and adolescents

Kellett, S., Beail, N., Newman, D. W., & Hawes, A. (2004). The factor structure of the Brief Symptom Inventory: Intellectual disability evidence. *Clinical Psychology & Psychotherapy, 11*(4), 275-281. 10.1002/cpp.410. Exclusion reason: Results not reported separately for children and adolescents

Kim, J. I., Shin, M. S., Lee, Y., Lee, H., Yoo, H. J., Kim, S. Y., . . . Kim, B. N. (2018). Reliability and Validity of a New Comprehensive Tool for Assessing Challenging Behaviors in Autism Spectrum Disorder. *Psychiatry Investigation, 15*(1), 54-61. 10.4306/pi.2018.15.1.54. Exclusion reason: No intellectual disability information

Kim, J. Y., & Ha, E. H. (2019). Cluster Analysis of the Child Behavior Checklist 1.5-5 for Preschool Children Diagnosed With a Mental Disorder. *Psychological Reports*, 33294119844980. 10.1177/0033294119844980. Exclusion reason: Mean age < 4 years

Kishore, M., Nizamie, A., Nizamie, S., & Jahan, M. (2004). Psychiatric diagnosis in persons with intellectual disability in India. *Journal of Intellectual Disability Research, 48*(1), 19-24. 10.1111/j.1365-2788.2004.00579.x. Exclusion reason: Results not reported separately for children and adolescents

Kishore, M., Nizamie, S., & Nizamie, A. (2005). The behavioural profile of psychiatric disorders in persons with intellectual disability. *Journal of Intellectual Disability Research, 49*(11), 852-857. 10.1111/j.1365-2788.2005.00763.x. Exclusion reason: Results not reported separately for children and adolescents

Kleczek-Atkins, A. (2004). Parents' and teachers' reports of behavioral and emotional problems in children with learning disabilities. *Dissertation Abstracts International: Section B: The Sciences and Engineering, 64*(9-B), 4620. Exclusion reason: No intellectual disability information

Klein-Tasman, B. P. (2001). Distinctive personality and behavioral characteristics of 8-, 9-, and 10-year-old children with Williams syndrome. *Dissertation Abstracts International: Section B: The Sciences and Engineering, 61*(8-B), 4464. Exclusion reason: Not relevant measurement tool

Koller, H., Richardson, S. A., Katz, M., & McLaren, J. (1983). Behavior disturbance since childhood among a 5-year birth cohort of all mentally retarded young adults in a city. *American Journal of Mental Deficiency, 87*(4), 386-395. Exclusion reason: No psychometric information

Koulopoulou, A. (2010). Anxiety and depression symptoms in children-commorbidity with learning disabilities. *European Psychiatry. Conference: 18th European Congress of Psychiatry. Munich Germany. Conference Publication:, 25*(SUPPL. 1). 10.1016/S0924-9338%2810%2970427-2. Exclusion reason: Conference Abstract

Kourkounasiou, M. A., & Skordilis, E. K. (2014). Validity and reliability evidence of the TOCA-C in a sample of Greek students. *Psychological Reports, 115*(3), 766-783. 10.2466/08.11.PR0.115c31z5. Exclusion reason: Results not reported separately for children and adolescents

Krener, P., & Simmons, M. K. (1989). The Child Consultation Rating Scale. *International Journal of Psychiatry in Medicine, 19*(1), 23-39. 10.2190/77MR-G1JC-AH3Y-KFK3. Exclusion reason: No intellectual disability information

Kuhn, D. E., & Matson, J. L. (2002). A validity study of the Screening Tool of Feeding Problems (STEP). *Journal of Intellectual and Developmental Disability, 27*(3), 161-167. 10.1080/1366825021000008594. Exclusion reason: Not relevant measurement tool

Kurtz, P. F., Chin, M. D., Huete, J. M., & Cataldo, M. F. (2012). Identification of Emerging Self-Injurious Behavior in Young Children: A Preliminary Study. *Journal of Mental Health Research in Intellectual Disabilities, 5*(3-4), 260-285. Exclusion reason: Mean age < 4 years

la Greca, A. M., Stone, W. L., & Bell, C. R. (1982). Assessing the problematic interpersonal skills of mentally retarded individuals in a vocational setting. *Applied Research in Mental Retardation, 3*(1), 37-53. 10.1016/0270-3092%2882%2990057-1. Exclusion reason: Not relevant measurement tool

Lachiewicz, A. M., & Dawson, D. V. (1994). Behavior problems of young girls with fragile X syndrome: factor scores on the Conners' Parent's Questionnaire. *American Journal of Medical Genetics, 51*(4), 364-369. Exclusion reason: No psychometric information

Lecavalier, L. (2006). Behavioral and emotional problems in young people with pervasive developmental disorders: Relative prevalence, effects of subject characteristics, and empirical classification. *Journal of Autism and Developmental Disorders, 36*(8), 1101-1114. 10.1007/s10803-006-0147-5. Exclusion reason: No intellectual disability information

Lecavalier, L., Aman, M. G., Hammer, D., Stoica, W., & Mathews, G. L. (2004). Factor Analysis of the Nisonger Child Behavior Rating Form in Children with Autism Spectrum Disorders. *Journal of Autism and Developmental Disorders, 34*(6), 709-721. 10.1007/s10803-004-5291-1. Exclusion reason: No intellectual disability information

Lecavalier, L., Gadow, K. D., DeVincent, C. J., & Edwards, M. C. (2009). Validation of DSM-IV Model of Psychiatric Syndromes in Children with Autism Spectrum Disorders. *Journal of Autism and Developmental Disorders, 39*(2), 278-289. 10.1007/s10803-008-0622-2. Exclusion reason: Wrong patient population

Lecavalier, L., Gadow, K. D., Devincent, C. J., Houts, C. R., & Edwards, M. C. (2011). Validity of DSM-IV syndromes in preschoolers with autism spectrum disorders. *Autism, 15*(5), 527-543. 10.1177/1362361310391115. Exclusion reason: Wrong patient population

Lecavalier, L., McCracken, C. E., Aman, M. G., McDougle, C. J., McCracken, J. T., Tierney, E., . . . Scahill, L. (2019). An exploration of concomitant psychiatric disorders in children with autism spectrum disorder. *Comprehensive Psychiatry, 88*, 57-64. 10.1016/j.comppsych.2018.10.012. Exclusion reason: Review

Lehotkay, R., Saraswathi Devi, T., Raju, M., Bada, P., Nuti, S., Kempf, N., & Carminati, G. (2015). Factor validity and reliability of the Aberrant Behavior Checklist-Community (ABC-C) in an Indian population with intellectual disability. *Journal of Intellectual Disability Research, 59*(3), 208-214. 10.1111/jir.12128. Exclusion reason: Adult population

Leung, P. W., Luk, S., & Lee, P. L. (1989). Problem behaviour among special school children in Hong Kong: A factor-analytical study with Conners' Teacher Rating Scale. *Psychologia: An International Journal of Psychology in the Orient, 32*(2), 120-128. Exclusion reason: Not relevant outcome

Leyfer, O. T., Folstein, S. E., Bacalman, S., Davis, N. O., Dinh, E., Morgan, J., . . . Lainhart, J. E. (2006). Comorbid psychiatric disorders in children with autism: Interview development and rates of disorders. *Journal of Autism and Developmental Disorders, 36*(7), 849-861. 10.1007/s10803-006-0123-0. Exclusion reason: Wrong patient population

Liddon, C. J., Zarcone, J. R., Pisman, M., & Rooker, G. W. (2016). Examination of behavioral flexibility and function of severe challenging behavior in individuals with autism and intellectual disability. *International Journal of Developmental Disabilities, 62*(3), 167-173. 10.1080/20473869.2016.1185248. Exclusion reason: Not relevant measurement tool

Lindblad, I., Gillberg, C., & Fernell, E. (2011). ADHD and other associated developmental problems in children with mild mental retardation. The use of the "Five-To-Fifteen" questionnaire in a population-based sample. *Research in Developmental Disabilities, 32*(6), 2805-2809. 10.1016/j.ridd.2011.05.026. Exclusion reason: No psychometric information

Linna, S. L., Moilanen, I., Ebeling, H., Piha, J., Kumpulainen, K., Tamminen, T., & Almqvist, F. (1999). Psychiatric symptoms in children with intellectual disability. *European Child & Adolescent Psychiatry, 8 Suppl 4*, 77-82. Exclusion reason: Duplicate reference

Linna, S.-L., Moilanen, I., Ebeling, H., Piha, J., Kumpulainen, K., Tamminen, T., & Almqvist, F. (1999). Psychiatric symptoms in children with intellectual disability. *European Child & Adolescent Psychiatry, 8*(Suppl 4), 77-82. 10.1007/PL00010704. Exclusion reason: No psychometric information

Linsell, L., Johnson, S., Wolke, D., Morris, J., Kurinczuk, J. J., & Marlow, N. (2018). A trajectories of behavior, attention, social and emotional problems from childhood to early adulthood following extremely preterm birth: A prospective cohort study. *European Child & Adolescent Psychiatry*, No Pagination Specified. 10.1007/s00787-018-1219-8. Exclusion reason: Wrong patient population

Liu, J., Jia, M.-X., Yang, X.-L., Shao, C.-X., Li, Y., Shi, J.-L., . . . Liang, A.-M. (2012). A 3-year follow-up study of 2-6 years children with diagnosis of pervasive developmental disorder. *Chinese Mental Health Journal, 26*(6), 460-465. Exclusion reason: Article in foreign language/not accessible language

Livingston, R. L., Dykman, R. A., & Ackerman, P. T. (1990). The frequency and significance of additional self-reported psychiatric diagnoses in children with attention deficit disorder. *Journal of Abnormal Child Psychology, 18*(5), 465-478. Exclusion reason: No intellectual disability information

Louwerse, A., Eussen, M., Ende, J., Nijs, P., Gool, A., Dekker, L., . . . Greaves-Lord, K. (2015). ASD Symptom Severity in Adolescence of Individuals Diagnosed with PDD-NOS in Childhood: Stability and the Relation with Psychiatric Comorbidity and Societal Participation. *Journal of Autism & Developmental Disorders, 45*(12), 3908-3918. 10.1007/s10803-015-2595-2. Exclusion reason: No intellectual disability information

Lu, T.-F., Shuai, L., Zhang, J.-S., Wang, Y.-F., Qian, Y., Zhang, H.-F., . . . Tan, X. (2017). Validity and reliability of the Behavior Rating Scale of Executive Function-Preschool Version parent form in China. *Chinese Mental Health Journal, 31*(2), 138-143. Exclusion reason: Not relevant outcome

Lund, J., & Merrell, K. W. (2001). Social and anitsocial behavior of children with learning and behavioral disorders: Construct validity of the Home and Community Social Behavior Scales. *Journal of Psychoeducational Assessment, 19*(2), 112-122. 10.1177/073428290101900201. Exclusion reason: No intellectual disability information

Lund, J., & Merrell, K. W. (2001). Social and antisocial behavior of children with learning and behavioral disorders: Construct validity of the Home and Community Social Behavior Scales. *Journal of Psychoeducational Assessment, 19*(2), 112-122. 10.1177/073428290101900201. Exclusion reason: No intellectual disability information

Lyon, M. A., Albertus, C., Birkinbine, J., & Naibi, J. (1996). A validity study of the social skills rating system-teacher version with disabled and nondisabled preschool children. *Perceptual and Motor Skills, 83*(1), 307-316. 10.2466/pms.1996.83.1.307. Exclusion reason: Not relevant measurement tool

Lyons, G. L., Huber, H. B., Carter, E. W., Chen, R., & Asmus, J. M. (2016). Assessing the social skills and problem behaviors of adolescents with severe disabilities enrolled in general education classes. *American Journal on Intellectual and Developmental Disabilities, 121*(4), 327-345. 10.1352/1944-7558-121.4.327. Exclusion reason: Duplicate reference

Maag, J. W., Irvin, D. M., Reid, R., & Vasa, S. F. (1994). Prevalence and predictors of substance use: A comparison between adolescents with and without learning disabilities. *Journal of Learning Disabilities, 27*(4), 223-234. 10.1177/002221949402700404. Exclusion reason: Not relevant measurement tool

Maag, J. W., & Reid, R. (2006). Depression among Students with Learning Disabilities: Assessing the Risk. *Journal of Learning Disabilities, 39*(1), 3-10. Exclusion reason: Review

Maag, J. W., & Rutherford, R. B. (1986). Perceived social competence of behaviorally disordered, learning disabled, and nonlabeled students. *Journal of Instructional Psychology, 13*(1), 10-18. Exclusion reason: Wrong patient population

Maas, A. P., Didden, R., Korzilius, H., Braam, W., Collin, P., Smits, M. G., & Curfs, L. M. (2011). Psychometric properties of a sleep questionnaire for use in individuals with intellectual disabilities. *Research in Developmental Disabilities, 32*(6), 2467-2479. 10.1016/j.ridd.2011.07.013. Exclusion reason: Results not reported separately for children and adolescents

MacDonald, L., & Barton, L. E. (1986). Measuring severity of behavior: A revision of Part II of the Adaptive Behavior Scale. *American Journal of Mental Deficiency, 90*(4), 418-424. Exclusion reason: Not relevant measurement tool

Maes, B., Broekman, T., Dosen, A., & Nauts, J. (2003). Caregiving burden of families looking after persons with intellectual disability and behavioural or psychiatric problems. *Journal of Intellectual Disability Research, 47*(6), 447-455. 10.1046/j.1365-2788.2003.00513.x. Exclusion reason: No psychometric information

Magyar, C. I., & Pandolfi, V. (2017). Utility of the CBCL DSM-oriented scales in assessing emotional disorders in youth with autism. *Research in Autism Spectrum Disorders, 37*, 11-20. 10.1016/j.rasd.2017.01.009. Exclusion reason: No intellectual disability information

Mahan, S., & Matson, J. L. (2011). Convergent and discriminant validity of the Autism Spectrum Disorder-Problem Behavior for Children (ASD-PBC) against the Behavioral Assessment System for Children, Second Edition (BASC-2). *Research in Autism Spectrum Disorders, 5*(1), 222-229. 10.1016/j.rasd.2010.04.003. Exclusion reason: No intellectual disability information

Maheady, L., & Harper, G. F. (1986). The Social Perception Behavior Rating Scale: Initial Evidence. *Diagnostique, 11*(2), 91-103. Exclusion reason: Not relevant measurement tool

Maiano, C., Morin, A. J. S., Begarie, J., & Ninot, G. (2011). The intellectual disability version of the very short form of the physical self-inventory (PSI-VS-ID): Cross-validation and measurement invariance across gender, weight, age and intellectual disability level. *Research in Developmental Disabilities, 32*(5), 1652-1662. 10.1016/j.ridd.2011.02.019. Exclusion reason: Adult population

Malovic, A., Cygan, L., Richards, S., Murphy, G., & Rossiter, R. (2016). Adolescents with intellectual and developmental disabilities who display harmful sexual behaviours: Adaptation of measures. *Journal of Intellectual Disability Research, 60 (7-8)*, 700. Exclusion reason: Not relevant measurement tool

Mammarella, I. C., Ghisi, M., Bomba, M., Bottesi, G., Caviola, S., Broggi, F., & Nacinovich, R. (2016). Anxiety and Depression in Children With Nonverbal Learning Disabilities, Reading Disabilities, or Typical Development. *Journal of Learning Disabilities, 49*(2), 130-139. 10.1177/0022219414529336. Exclusion reason: Wrong patient population

Manikam, R., Matson, J. L., Coe, D. A., & Hillman, N. (1995). Adolescent depression: Relationships of self-report to intellectual and adaptive functioning. *Research in Developmental Disabilities, 16*(5), 349-364. 10.1016/0891-4222%2895%2900018-I. Exclusion reason: Not relevant outcome

Margalit, M., Shulman, S., & Stuchiner, N. (1989). Behavior disorders and mental retardation: The family system perspective. *Research in Developmental Disabilities, 10*(3), 315-326. 10.1016/0891-4222%2889%2990019-X. Exclusion reason: No psychometric information

Marland, C., Lichtenstein, P., Degl'Innocenti, A., Larson, T., Rastam, M., Anckarsater, H., . . . Lundstrom, S. (2017). The Autism-Tics, ADHD and other Comorbidities inventory (A-TAC): Previous and predictive validity. *BMC Psychiatry Vol 17 2017, ArtID 403, 17*. 10.1186/s12888-017-1563-0. Exclusion reason: Wrong patient population

Marshall, K., Coiffait, F.-M., & Willoughby-Booth, S. (2013). Assessing distress in people with intellectual disabilities. *Learning Disability Practice, 16*(3), 26-30. Exclusion reason: Adult population

Masi, G., Mucci, M., Favilla, L., & Poli, P. (1999). Dysthymic disorder in adolescents with intellectual disability. *Journal of Intellectual Disability Research, 43*(2), 80-87. 10.1046/j.1365-2788.1999.00195.x. Exclusion reason: No psychometric information

Matson, J. L., Belva, B. C., Hattier, M. A., & Matson, M. L. (2012). Scaling methods to measure psychopathology in persons with intellectual disabilities. *Research in Developmental Disabilities, 33*(2), 549-562. 10.1016/j.ridd.2011.10.023. Exclusion reason: Review

Matson, J. L., Bielecki, J., Mayville, E. A., Smalls, Y., Bamburg, J. W., & Baglio, C. S. (1999). The development of a reinforcer choice assessment scale for persons with severe and profound mental retardation. *Research in Developmental Disabilities, 20*(5), 379-384. 10.1016/s0891-4222(99)00018-9. Exclusion reason: Not relevant outcome

Matson, J. L., Boisjoli, J., Rojahn, J., & Hess, J. (2009). A factor analysis of challenging behaviors assessed with the Baby and Infant Screen for Children with aUtism Traits (BISCUIT-Part 3). *Research in Autism Spectrum Disorders, 3*(3), 714-722. 10.1016/j.rasd.2009.01.008. Exclusion reason: Mean age < 4 years

Matson, J. L., Fodstad, J. C., & Mahan, S. (2009). Cutoffs, norms, and patterns of comorbid difficulties in children with developmental disabilities on the Baby and Infant Screen for Children with aUtIsm Traits (BISCUIT-Part 2). *Research in Developmental Disabilities, 30*(6), 1221-1228. 10.1016/j.ridd.2009.04.004. Exclusion reason: Mean age < 4 years

Matson, J. L., Fodstad, J. C., Mahan, S., & Rojahn, J. (2010). Cut-offs, norms and patterns of problem behaviours in children with developmental disabilities on the Baby and Infant Screen for Children with aUtIsm Traits (BISCUIT-Part 3). *Developmental Neurorehabilitation, 13*(1), 3-9. 10.3109/17518420903074887. Exclusion reason: Mean age < 4 years

Matson, J. L., Fodstad, J. C., Mahan, S., & Sevin, J. A. (2009). Cutoffs, norms, and patterns of comorbid difficulties in children with an ASD on the Baby and Infant Screen for Children with aUtIsm Traits (BISCUIT-Part 2). *Research in Autism Spectrum Disorders, 3*(4), 977-988. 10.1016/j.rasd.2009.06.001. Exclusion reason: Mean age < 4 years

Matson, J. L., Gonzalez, M. L., & Rivet, T. T. (2008). Reliability of the Autism Spectrum Disorder-Behavior Problems for Children (ASD-BPC). *Research in Autism Spectrum Disorders, 2*(4), 696-706. 10.1016/j.rasd.2008.02.003. Exclusion reason: Wrong patient population

Matson, J. L., Hess, J. A., & Boisjoli, J. A. (2010). Comorbid psychopathology in infants and toddlers with autism and pervasive developmental disorders-not otherwise specified (PDD-NOS). *Research in Autism Spectrum Disorders, 4*(2), 300-301. 10.1016/j.rasd.2009.10.001. Exclusion reason: Mean age < 4 years

Matson, J. L., & Kuhn, D. E. (2001). Identifying feeding problems in mentally retarded persons: Development and reliability of the screening tool of feeding problems (STEP). *Research in Developmental Disabilities, 22*(2), 165-172. 10.1016/S0891-4222%2801%2900065-8. Exclusion reason: Not relevant measurement tool

Matson, J. L., Kuhn, D. E., Dixon, D. R., Mayville, S. B., Laud, R. B., Cooper, C. L., . . . Matson, M. L. (2003). The development and factor structure of the Functional Assessment for multiple causaliTy (FACT). *Research in Developmental Disabilities, 24*(6), 485-495. 10.1016/j.ridd.2003.07.001. Exclusion reason: Results not reported separately for children and adolescents

Matson, J. L., LoVullo, S. V., Rivet, T. T., & Boisjoli, J. A. (2009). Validity of the Autism Spectrum Disorder-Comorbid for Children (ASD-CC). *Research in Autism Spectrum Disorders, 3*(2), 345-357. 10.1016/j.rasd.2008.08.002. Exclusion reason: Results not reported separately for children and adolescents

Matson, J. L., Macklin, G., & Helsel, W. J. (1985). Psychometric properties of the Matson Evaluation of Social Skills with youngsters (MESSY) with emotional problems and self concept in deaf children. *Journal of Behavior Therapy and Experimental Psychiatry, 16*(2), 117-123. 10.1016/0005-7916%2885%2990046-1. Exclusion reason: Not relevant measurement tool

Matson, J. L., Mahan, S., Hess, J. A., Fodstad, J. C., & Neal, D. (2010). Progression of challenging behaviors in children and adolescents with Autism Spectrum Disorders as measured by the Autism Spectrum Disorders-Problem Behaviors for Children (ASD-PBC). *Research in Autism Spectrum Disorders, 4*(3), 400-404. 10.1016/j.rasd.2009.10.010. Exclusion reason: No intellectual disability information

Matson, J. L., & Malone, C. J. (2006). Validity of the sleep subscale of the Diagnostic Assessment for the Severely Handicapped-II (DASH-II). *Research in Developmental Disabilities, 27*(1), 85-92. Exclusion reason: Not relevant measurement tool

Matson, J. L., Mayville, S. B., & Laud, R. B. (2003). A System of Assessment for Adaptive Behavior, Social Skills, Behavioral Function, Medication Side-Effects, and Psychiatric Disorders. In (Vol. 24, pp. 75-81): Research in Developmental Disabilities. Exclusion reason: Theoretical article/Comment

Matson, J. L., & Minshawi, N. F. (2007). Functional assessment of challenging behavior: Toward a strategy for applied settings. *Research in Developmental Disabilities, 28*(4), 353-361. 10.1016/j.ridd.2006.01.005. Exclusion reason: Review

Matson, J. L., Minshawi, N. F., Gonzalez, M. L., & Mayville, S. B. (2006). The relationship of comorbid problem behaviors to social skills in persons with profound mental retardation. *Behavior Modification, 30*(4), 496-506. 10.1177/0145445505283415. Exclusion reason: Adult population

Matson, J. L., & Neal, D. (2009). Diagnosing comorbid psychiatric conditions: autism spectrum disorders. *Psychiatric Times, 26*(4), 38-44. Exclusion reason: Theoretical article/Comment

Matson, J. L., Neal, D., Fodstad, J. C., & Hess, J. A. (2010). The relation of social behaviours and challenging behaviours in infants and toddlers with autism spectrum disorders. *Developmental Neurorehabilitation, 13*(3), 164-169. 10.3109/17518420903270683. Exclusion reason: Mean age < 4 years

Matson, J. L., Wilkins, J., Sevin, J. A., Knight, C., Boisjoli, J. A., & Sharp, B. (2009). Reliability and item content of the Baby and Infant Screen for Children with aUtIsm Traits (BISCUIT): Parts 1-3. *Research in Autism Spectrum Disorders, 3*(2), 336-344. 10.1016/j.rasd.2008.08.001. Exclusion reason: Mean age < 4 years

Mattison, R. E., Bagnato, S. J., Mayes, S. D., & Felix, B. C. (1990). Reliability and validity of teacher diagnostic ratings for children with behavioral and emotional disorders. *Journal of Psychoeducational Assessment, 8*(4), 509-517. 10.1177/073428299000800406. Exclusion reason: Wrong patient population

May, M. E., Sheng, Y., Chitiyo, M., Brandt, R. C., & Howe, A. P. (2014). Internal consistency and inter-rater reliability of the Questions About Behavioral Function (QABF) rating scale when used by teachers and paraprofessionals. *Education & Treatment of Children, 37*(2), 347-364. 10.1353/etc.2014.0013. Exclusion reason: Not relevant measurement tool

Mayo-Ortega, L., Oyama-Ganiko, R., Leblanc, J., Schroeder, S. R., Brady, N., Butler, M. G., . . . Marquis, J. (2012). Mass screening for severe problem behavior among infants and toddlers in Peru. *Journal of Mental Health Research in Intellectual Disabilities, 5*(3-4), 246-259. 10.1080/19315864.2011.590626. Exclusion reason: Mean age < 4 years

McCarthy, J. (2008). Behaviour problems and adults with Down syndrome: childhood risk factors. *Journal of Intellectual Disability Research, 52*(10), 877-882. Exclusion reason: No psychometric information

McCarthy, J., & Boyd, J. (2002). Mental health services and young people with intellectual disability: Is it time to do better? *Journal of Intellectual Disability Research, 46*(3), 250-256. 10.1046/j.1365-2788.2002.00401.x. Exclusion reason: No psychometric information

McClean, B., & Grey, I. (2012). A component analysis of positive behaviour support plans. *Journal of Intellectual & Developmental Disability, 37*(3), 221-231. 10.3109/13668250.2012.704981. Exclusion reason: Not relevant measurement tool

McConaughy, S. H., & Achenbach, T. M. (1996). Contributions of a child interview to multimethod assessment of children with EBD and LD. *School Psychology Review, 25*(1), 24-39. Exclusion reason: Wrong patient population

McConaughy, S. H., Mattison, R. E., & Peterson, R. L. (1994). Behavioral/emotional problems of children with serious emotional disturbances and learning disabilities. *School Psychology Review, 23*(1), 81-98. Exclusion reason: Wrong patient population

McCormick, C., Hepburn, S., Young, G. S., & Rogers, S. J. (2016). Sensory Symptoms in Children with Autism Spectrum Disorder, Other Developmental Disorders and Typical Development: A Longitudinal Study. *Autism: The International Journal of Research and Practice, 20*(5), 572-579. Exclusion reason: Not relevant measurement tool

McDaniel, W. (1997). Criterion-related diagnostic validity and test-retest reliability of the MMPI-168(L) in mentally retarded adolescents and adults. *Journal of Clinical Psychology, 53*(5), 485-489. 10.1002/%28SICI%291097-4679%28199708%2953:5%3C485::AID-JCLP10%3E3.0.CO;2-A. Exclusion reason: Adult population

McDaniel, W., Childers, L., & Compton, D. (1997). Construct validity of the MMPI-168 (L) with mentally retarded adults and adolescents. *Journal of Clinical Psychology, 53*(7), 727-732. 10.1002/%28SICI%291097-4679%28199711%2953:7%3C727::AID-JCLP10%3E3.0.CO;2-N. Exclusion reason: Not relevant measurement tool

McGill, P., Hughes, D., Teer, K., & Rye, L. (2001). Variability in staff reports of the frequency of challenging behavior. *Research in Developmental Disabilities, 22*(3), 221-231. 10.1016/S0891-4222%2801%2900069-5. Exclusion reason: Results not reported separately for children and adolescents

McGrew, K. S., Ittenbach, R. F., Bruininks, R. H., & Hill, B. K. (1991). Factor structure of maladaptive behavior across the lifespan of persons with mental retardation. *Research in Developmental Disabilities, 12*(2), 181-199. 10.1016/0891-4222%2891%2990005-D. Exclusion reason: Not relevant measurement tool

McGrew, S., Malow, B. A., Henderson, L., Wang, L., Song, Y., & Stone, W. L. (2007). Developmental and behavioral questionnaire for autism spectrum disorders. *Pediatric Neurology, 37*(2), 108-116. Exclusion reason: Wrong patient population

McLennan, J. D. (2016). Measures to assess outcomes in routine psychotropic medication treatment of youth with autism or intellectual disabilities. *Journal of the American Academy of Child and Adolescent Psychiatry, 55 (10 Supplement 1)*, S169-S170. 10.1016/j.jaac.2016.09.216. Exclusion reason: Conference abstract

McMahon, J., Harvey, A., May, T., Reid, S., & Antolovich, G. (2018). Anxiety in children with cerebral palsy - Underdiagnosed and poorly measured. *Developmental Medicine and Child Neurology, 60 (Supplement 1)*, 17. 10.1111/dmcn.13665. Exclusion reason: Conference abstract

Medeiros, K., Curby, T. W., Bernstein, A., Rojahn, J., & Schroeder, S. R. (2013). The progression of severe behavior disorder in young children with intellectual and developmental disabilities. *Research in Developmental Disabilities, 34*(11), 3639-3647. 10.1016/j.ridd.2013.08.002. Exclusion reason: Mean age < 4 years

Medeiros, K., Kozlowski, A. M., Beighley, J. S., Rojahn, J., & Matson, J. L. (2012). The effects of developmental quotient and diagnostic criteria on challenging behaviors in toddlers with developmental disabilities. *Research in Developmental Disabilities, 33*(4), 1110-1116. 10.1016/j.ridd.2012.02.005. Exclusion reason: Mean age < 4 years

Meehan, M. L., & Appalachia Educational Lab, C. W. V. (1980). *Third year Evaluation of a Model Home-Based Program for Severely Orthopedically Impaired/Mentally Retarded Children & Youth*. Retrieved from http://search.ebscohost.com/login.aspx?direct=true&db=eric&AN=ED216012&site=ehost-live Exclusion reason: Not relevant measurement tool

Meester-Delver, A., Beelen, A., Hennekam, R., Nollet, F., & Hadders-Algra, M. (2007). The Capacity Profile: A method to classify additional care needs in children with neurodevelopmental disabilities. *Developmental Medicine and Child Neurology, 49*(5), 355-360. 10.1111/j.1469-8749.2007.00355.x. Exclusion reason: No intellectual disability information

Mekori-Domachevsky, E., Guri, Y., Yi, J., Weisman, O., Calkins, M. E., Tang, S. X., . . . Gothelf, D. (2017). Negative subthreshold psychotic symptoms distinguish 22q11.2 deletion syndrome from other neurodevelopmental disorders: A two-site study. *Schizophrenia Research, 188*, 42-49. 10.1016/j.schres.2016.12.023. Exclusion reason: Results not reported separately for children and adolescents

Merikle, E., Patel, V., Sebree, T. B., Kreusser, C., & Heussler, H. (2019). PRO10 QUALITATIVE STUDY TO ESTABLISH THE CONTENT VALIDITY OF THE ABC-C<inf>FXS</inf> FOR EVALUATION OF TREATMENT EFFICACY IN FRAGILE X SYNDROME. *Value in Health, 22 (Supplement 3)*, S842. 10.1016/j.jval.2019.09.2342. Exclusion reason: Conference abstract

Merrell, K. W., & Holland, M. L. (1997). Social-emotional behavior of preschool-age children with and without developmental delays. *Research in Developmental Disabilities, 18*(6), 393-405. Exclusion reason: No intellectual disability information

Merrell, K. W., & Popinga, M. R. (1994). The alliance of adaptive behavior and social competence: an examination of relationship between the scales of Independent Behavior and the Social Skills Rating System. *Research in Developmental Disabilities, 15*(1), 39-47. Exclusion reason: Wrong patient population

Merrell, K. W., & Popinga, M. R. (1994). The alliance of adaptive behavior and social competence: An examination of relationships between the Scales of Independent Behavior and the Social Skills Rating System. *Research in Developmental Disabilities, 15*(1), 39-47. 10.1016/0891-4222%2894%2990037-X. Exclusion reason: Not relevant outcome

Messinger, D., Lambert, B., Bauer, C. R., Bann, C. M., Hamlin-Smith, K., & Das, A. (2010). The relationship between behavior ratings and concurrent and subsequent mental and motor performance in toddlers born at extremely low birth weight. *Journal of Early Intervention, 32*(3), 214-233. 10.1177/1053815110380917. Exclusion reason: Mean age < 4 years

Mevissen, L., Didden, R., Korzilius, H., & de Jongh, A. (2016). Assessing posttraumatic stress disorder in children with mild to borderline intellectual disabilities. *European Journal of Psychotraumatology Vol 7 2016, ArtID 29786, 7*. 10.3402/ejpt.v7.29786. Exclusion reason: Not relevant measurement tool

Mildenberger, K., Noterdaeme, M., Sitter, S., & Amorosa, H. (2001). Behavioral problems in children with specific and pervasive developmental disorders, evaluated with the psychopathology assessment scale (AMDP). [German]. *Praxis der Kinderpsychologie und Kinderpsychiatrie, 50*(8), 649-663. Exclusion reason: Article in foreign language/not accessible language

Moehle, K. A., & Fitzhugh-Bell, K. B. (1989). Factor analysis of the Conners Teacher Rating Scale with brain-damaged and learning-disabled children. *Psychology in the Schools, 26*(2), 113-125. 10.1002/1520-6807%28198904%2926:2%3C113::AID-PITS2310260202%3E3.0.CO;2-6. Exclusion reason: Wrong patient population

Mohr, C., & Gray, K. M. (2005). Assessment in intellectual disability. *Current Opinion in Psychiatry, 18*(5), 476-483. 10.1097/01.yco.0000179483.62391.12. Exclusion reason: Review

Molteno, G., Molteno, C. D., Finchilescu, G., & Dawes, A. R. (2001). Behavioural and emotional problems in children with intellectual disability attending special schools in Cape Town, South Africa. *Journal of Intellectual Disability Research, 45*(Pt 6), 515-520. Exclusion reason: No psychometric information

Moore, L. S. (1995). The relationship between neuropsychological, academic, and psychosocial variables in determining behavioral/emotional problems in learning disabled youth. *Dissertation Abstracts International: Section B: The Sciences and Engineering, 56*(2-B), 1116. Exclusion reason: No psychometric information

Morgan, L., Wetherby, A. M., & Barber, A. (2008). Repetitive and Stereotyped Movements in Children with Autism Spectrum Disorders Late in the Second Year of Life. *Journal of Child Psychology and Psychiatry, 49*(8), 826-837. Exclusion reason: Mean age < 4 years

Morris, C., Janssens, A., Allard, A., Thompson Coon, J., Shilling, V., Tomlinson, R., . . . Logan, S. (2014). *NIHR Journals Library. Health Services and Delivery Research, 05*, 05. 10.3310/hsdr02150. Exclusion reason: No intellectual disability information

Moskowitz, L. J., Mulder, E., Walsh, C. E., McLaughlin, D. M., Zarcone, J. R., Proudfit, G. H., & Carr, E. G. (2013). A multimethod assessment of anxiety and problem behavior in children with autism spectrum disorders and intellectual disability. *American Journal on Intellectual and Developmental Disabilities, 118*(6), 419-434. 10.1352/1944.7558.118.6.419. Exclusion reason: Not relevant measurement tool

Mosner, M. G., Kinard, J. L., Shah, J. S., McWeeny, S., Greene, R. K., Lowery, S. C., . . . Dichter, G. S. (2019). Rates of Co-occurring Psychiatric Disorders in Autism Spectrum Disorder Using the Mini International Neuropsychiatric Interview. *Journal of Autism & Developmental Disorders, 49*(9), 3819-3832. 10.1007/s10803-019-04090-1. Exclusion reason: Wrong patient population

Murphy, O., Healy, O., & Leader, G. (2009). Risk factors for challenging behaviors among 157 children with autism spectrum disorder in Ireland. *Research in Autism Spectrum Disorders, 3*(2), 474-482. 10.1016/j.rasd.2008.09.008. Exclusion reason: Wrong patient population

Myrbakk, E., & von Tetzchner, S. (2008). Screening individuals with intellectual disability for psychiatric disorders: Comparison of four measures. *American Journal on Mental Retardation, 113*(1), 54-70. 10.1352/0895-8017%282008%29113%5B54:SIWIDF%5D2.0.CO;2. Exclusion reason: Results not reported separately for children and adolescents

Naglieri, J. A., & Gottling, S. H. (1995). Use of the Teacher Report Form and the Devereux Behavior Rating Scale-School Form with learning disordered/emotionally disordered students. *Journal of Clinical Child Psychology, 24*(1), 71-76. 10.1207/s15374424jccp2401_9. Exclusion reason: No psychometric information

Nair, R., Dutt, A., & Nielsen, T. (2019). Brief report: Psychometric properties of the ability in behavior assessment and interventions for teachers-revised (abait-r). *Journal of Autism and Developmental Disorders*, No Pagination Specified. 10.1007/s10803-019-04286-5. Exclusion reason: Not relevant measurement tool

Nair, R., Dutt, A., & Nielsen, T. (2020). Brief Report: Psychometric Properties of the Ability in Behavior Assessment and Interventions for Teachers-Revised (ABAIT-R). *Journal of Autism & Developmental Disorders, 50*(3), 1081-1087. 10.1007/s10803-019-04286-5. Exclusion reason: Not relevant outcome

Nakai, Y., Miyawaki, D., Kusaka, H., Okamoto, H., Futoo, E., Goto, A., . . . Inoue, K. (2013). Anxiety in children with high-functioning pervasive developmental disorder. *Osaka City Medical Journal, 59*(1), 23-34. Exclusion reason: Wrong patient population

Navas, P., Verdugo, M. A., Arias, B., & Gomez, L. E. (2012). Development of an instrument for diagnosing significant limitations in adaptive behavior in early childhood. *Research in Developmental Disabilities, 33*(5), 1551-1559. 10.1016/j.ridd.2012.03.006. Exclusion reason: Results not reported separately for children and adolescents

Neo, W. S., Suzuki, T., & Kelleher, B. L. (2021). Structural validity of the Child Behavior Checklist (CBCL) for preschoolers with neurogenetic syndromes. *Research in Developmental Disabilities, 109*, 103834. 10.1016/j.ridd.2020.103834. Exclusion reason: Mean age < 4 years

Newcomer, P. L., Barenbaum, E., & Pearson, N. (1995). Depression and anxiety in children and adolescents with learning disabilities, conduct disorders, and no disabilities. *Journal of Emotional and Behavioral Disorders, 3*(1), 27-39. 10.1177/106342669500300104. Exclusion reason: Wrong patient population

Newman, I., Leader, G., Chen, J. L., & Mannion, A. (2015). An analysis of challenging behavior, comorbid psychopathology, and Attention-Deficit/Hyperactivity Disorder in Fragile X Syndrome. *Research in Developmental Disabilities, 38*, 7-17. 10.1016/j.ridd.2014.11.003. Exclusion reason: No psychometric information

Niarchou, M., Chawner, S., Doherty, J. L., Maillard, A. M., Jacquemont, S., Chung, W. K., . . . Bree, M. (2019). Psychiatric disorders in children with 16p11.2 deletion and duplication. *Transl Psychiatry Psychiatry, 9*(1), 8. 10.1038/s41398-018-0339-8. Exclusion reason: Wrong patient population

Niarchou, M., Moore, T. M., Tang, S. X., Calkins, M. E., McDonald-McGuinn, D. M., Zackai, E. H., . . . Gur, R. E. (2017). The dimensional structure of psychopathology in 22q11.2 Deletion Syndrome. *Journal of Psychiatric Research, 92*, 124-131. 10.1016/j.jpsychires.2017.04.006. Exclusion reason: Wrong patient population

Niarchou, M., Zammit, S., van Goozen, S. H. M., Thapar, A., Tierling, H. M., Owen, M. J., & van den Bree, M. B. M. (2014). Psychopathology and cognition in children with 22q11.2 deletion syndrome. *British Journal of Psychiatry, 204*(1), 46-54. 10.1192/bjp.bp.113.132324. Exclusion reason: No psychometric information

Nicholson, J., Konstantinidi, E., & Furniss, F. (2006). On some psychometric properties of the questions about behavioral function (QABF) scale. *Research in Developmental Disabilities, 27*(3), 337-352. 10.1016/j.ridd.2005.04.001. Exclusion reason: Not relevant measurement tool

Nihira, K., Price-Williams, D. R., & White, J. F. (1988). Social competence and maladaptive behavior of people with dual diagnosis. *Journal of the Multihandicapped Person, 1*(3), 185-199. 10.1007/BF01102623. Exclusion reason: Not relevant measurement tool

Nodrick, W. S., & Li, A. K. (1992). Concurrent validity of the School Problem Screening Inventory for behavior-disordered students. *Psychology in the Schools, 29*(2), 126-131. 10.1002/1520-6807%28199204%2929:2%3C126::AID-PITS2310290206%3E3.0.CO;2-7. Exclusion reason: Wrong patient population

Nordness, P. D., Epstein, M. H., Cullinan, D., & Pierce, C. D. (2014). Emotional and Behavioral Screener: Test-Retest Reliability, Inter-Rater Reliability, and Convergent Validity. *Remedial and Special Education, 35*(4), 211-217. 10.1177/0741932513497596. Exclusion reason: Wrong patient population

O'Brien, G., Pearson, J., Berney, T., & Barnard, L. (2001). Measuring behaviour in developmental disability: a review of existing schedules. *Developmental Medicine & Child Neurology - Supplementum, 87*, 1-72. Exclusion reason: Conference abstract

Ono, Y. (1996). Factor validity and reliability for the Aberrant Behavior Checklist-Community in a Japanese population with mental retardation. *Research in Developmental Disabilities, 17*(4), 303-309. 10.1016/0891-4222%2896%2900015-7. Exclusion reason: Results not reported separately for children and adolescents

Ono, Y. (1996). Factor validity and reliability for the Aberrant behavior checklist-community in a Japanese population with mental retardation. Research in Developmental Disabilities, 17(4), 303-309. 10.1016/0891-4222(96)00015-7. Exclusion reason: Results not reported separately for children and adolescents

Oubrahim, L., & Combalbert, N. (2019). Frequency and origin (reactive/proactive) of aggressive behavior in young people with intellectual disability and autism spectrum disorder. *International Journal of Developmental Disabilities*, No Pagination Specified. 10.1080/20473869.2019.1640972. Exclusion reason: No psychometric information

Overall, J. E., & Campbell, M. (1988). Behavioral assessment of psychopathology in children: Infantile autism. *Journal of Clinical Psychology, 44*(5), 708-716. 10.1002/1097-4679%28198809%2944:5%3C708::AID-JCLP2270440507%3E3.0.CO;2-T. Exclusion reason: Wrong patient population

Paclawskyj, T. R., Matson, J. L., Rush, K. S., Smalls, Y., & Vollmer, T. R. (2001). Assessment of the convergent validity of the Questions About Behavioral Function scale with analogue functional analysis and the Motivation Assessment Scale. *Journal of Intellectual Disability Research, 45*(Pt 6), 484-494. Exclusion reason: Not relevant measurement tool

Painter, J., Trevithick, L., Hastings, R. P., Ingham, B., & Roy, A. (2016). Development and validation of the Learning Disabilities Needs Assessment Tool (LDNAT), a HoNOS-based needs assessment tool for use with people with intellectual disability. *Journal of Intellectual Disability Research, 60*(12), 1178-1188. 10.1111/jir.12340. Exclusion reason: Adult population

Pavlovic, M., Zunic-Pavlovic, V., & Glumbic, N. (2013). Students' and teachers' perceptions of aggressive behaviour in adolescents with intellectual disability and typically developing adolescents. *Research in Developmental Disabilities, 34*(11), 3789-3797. 10.1016/j.ridd.2013.07.035. Exclusion reason: Not relevant outcome

Pelham, W. E., Evans, S. W., Gnagy, E. M., & Greenslade, K. E. (1992). Teacher ratings of DSM-III-R symptoms for the disruptive behavior disorders: Prevalence, factor analyses, and conditional probabilities in a special education sample. *School Psychology Review, 21*(2), 285-299. Exclusion reason: No intellectual disability information

Pisecco, S., Lachar, D., Gruber, C. P., Gallen, R. T., Kline, R. B., & Huzinec, C. (1999). Development and validation of disruptive behavior scales for the student behavior survey (SBS). *Journal of Psychoeducational Assessment, 17*(4), 314-331. 10.1177/073428299901700402. Exclusion reason: Wrong patient population

Platt, J. M., Keyes, K. M., McLaughlin, K. A., & Kaufman, A. S. (2019). Intellectual disability and mental disorders in a US population representative sample of adolescents. *Psychological Medicine, 49*(6), 952-961. 10.1017/S0033291718001605. Exclusion reason: No psychometric information

Poindexter, A. R., & Glidden, L. M. (2006). Diagnosis of depression in people with developmental disabilities: Progress and problems. *International Review of Research in Mental Retardation, Vol 32, 32*, 261-281. 10.1016/s0074-7750(06)32009-5. Exclusion reason: Review

Porter, M., Byrne, A., Campbell, L., Mathieson, N., & Boulton, K. (2016). Social, psychological and behavioural functioning in Williams syndrome, Down syndrome and velo-cardiofacial syndrome: A cross-syndrome and individual difference approach. *Journal of Intellectual Disability Research, 60 (7-8)*, 721. Exclusion reason: Conference abstract

Porter, M. A., Dodd, H., & Cairns, D. (2009). Psychopathological and behavior impairments in Williams-Beuren syndrome: The influence of gender, chronological age, and cognition. *Child Neuropsychology, 15*(4), 359-374. 10.1080/09297040802577881. Exclusion reason: Results not reported separately for children and adolescents

Predescu, E., Sipos, R., Dobrean, A., & Miclutia, I. (2013). The discriminative power of the CBCL 1.5-5 between autism spectrum disorders and other psychiatric disorders. *Journal of Cognitive and Behavioral Psychotherapies, 13*(1), 75-87. Exclusion reason: Mean age < 4 years

Pruijssers, A., van Meijel, B., Maaskant, M., Teerenstra, S., & van Achterberg, T. (2017). The Diagnostic Guideline for Anxiety and Challenging Behaviour for Persons with Intellectual Disabilities: Preliminary Outcomes on Internalizing Problems, Challenging Behaviours, Quality of Life and Clients' Satisfaction. *Journal of Applied Research in Intellectual Disabilities, 30*(2), 242-254. 10.1111/jar.12235. Exclusion reason: Adult population

Quay, H. C., & Gredler, Y. (1981). Dimensions of problem behavior in institutionalized retardates. *Journal of Abnormal Child Psychology, 9*(4), 523-528. 10.1007/BF00917801. Exclusion reason: Results not reported separately for children and adolescents

Quinn, M., Carr, A., Carroll, L., & O'Sullivan, D. (2007). Parents Plus Programme 1: Evaluation of Its Effectiveness for Pre-School Children with Developmental Disabilities and Behavioural Problems. *Journal of Applied Research in Intellectual Disabilities, 20*(4), 345-359. Exclusion reason: Wrong patient population

Quinn, M., Carr, A., Carroll, L., & O'Sullivan, D. (2007). Parents Plus programmes I: Evaluation of its effectiveness for pre-school children with developmental disabilities and behavioural problems. *Journal of Applied Research in Intellectual Disabilities, 20*(4), 345-359. 10.1111/j.1468-3148.2006.00352.x. Exclusion reason: Wrong patient population

Rackauskaite, G., Bilenberg, N., Bech, B. H., Uldall, P., & Østergaard, J. R. (2016). Screening for psychopathology in a national cohort of 8- to 15-year-old children with cerebral palsy. *Research in Developmental Disabilities, 49*, 171-180. 10.1016/j.ridd.2015.11.019. Exclusion reason: Wrong patient population

Ralston, M. B., Fuerst, D. R., & Rourke, B. P. (2003). Comparison of the psychosocial typology of children with below average IQ to that of children with learning disabilities. *Journal of Clinical and Experimental Neuropsychology, 25*(2), 255-273. 10.1076/jcen.25.2.255.13645. Exclusion reason: Wrong patient population

Ram Gopal, C., & Rao, M. (1994). A study of behaviour disorders in moderately mentally retarded children and their relation to parental attitude. *Indian Journal of Clinical Psychology, 21*(2), 27-31. Exclusion reason: Not relevant outcome

Raspa, M., Sacco, P., Candrilli, S., Bishop, E., & Petrillo, J. (2016). Validity of a condition specific outcome measure for fragile X syndrome: The Aberrant Behaviour Checklist-utility index. *Journal of Intellectual Disability Research, 60*(9), 844-855. 10.1111/jir.12264. Exclusion reason: Results not reported separately for children and adolescents

Ray, A. E. (1990). Acquiescent response patterns of mildly and moderately mentally retarded adolescents on a self-report questionnaire: Implications for the assessment of emotional disorders. *Dissertation Abstracts International, 51*(2-B), 1016. Exclusion reason: Not relevant measurement tool

Reardon, T. C., Gray, K. M., & Melvin, G. A. (2015). Anxiety disorders in children and adolescents with intellectual disability: Prevalence and assessment. *Research in Developmental Disabilities, 36*, 175-190. 10.1016/j.ridd.2014.10.007. Exclusion reason: Review

Reddy, L. A., Pfeiffer, S. I., & Files-Hall, T. M. (2007). Use of the Devereux Scales of Mental Disorders for children and adolescents with emotional disturbance. *Journal of Psychoeducational Assessment, 25*(4), 356-372. 10.1177/0734282907303121. Exclusion reason: Wrong patient population

Reilly, C., Atkinson, P., Das, K. B., Chin, R. F., Aylett, S. E., Burch, V., . . . Neville, B. G. (2014). Screening for mental health disorders in active childhood epilepsy: population-based data. *Epilepsy Research, 108*(10), 1917-1926. 10.1016/j.eplepsyres.2014.09.028. Exclusion reason: No intellectual disability information

Reiss, S., & Havercamp, S. M. (1998). Toward a comprehensive assessment of fundamental motivation: Factor structure of the reiss profiles. *Psychological Assessment, 10*(2), 97-106. 10.1037/1040-3590.10.2.97. Exclusion reason: Adult population

Reiss, S., & Rojahn, J. (1993). Joint occurrence of depression and aggression in children and adults with mental retardation. *Journal of Intellectual Disability Research, 37*(3), 287-294. 10.1111/j.1365-2788.1993.tb01285.x. Exclusion reason: Results not reported separately for children and adolescents

Reynolds, C. R. (1980). Concurrent validity of "What I think and feel:" the Revised Children's Manifest Anxiety Scale. *Journal of Consulting and Clinical Psychology, 48*(6), 774-775. Exclusion reason: No intellectual disability information

Rice, L. J., Gray, K. M., Howlin, P., Taffe, J., Tonge, B. J., & Einfeld, S. L. (2015). The developmental trajectory of disruptive behavior in Down syndrome, fragile X syndrome, Prader-Willi syndrome and Williams syndrome. *American Journal of Medical Genetics. Part C, Seminars in Medical Genetics, 169*(2), 182-187. 10.1002/ajmg.c.31442. Exclusion reason: No psychometric information

Richards, C., Moss, J., Nelson, L., & Oliver, C. (2016). Persistence of self-injurious behaviour in autism spectrum disorder over 3 years: a prospective cohort study of risk markers. *Journal of Neurodevelopmental Disorders, 8*. 10.1186/s11689-016-9153-x. Exclusion reason: No intellectual disability information

Rodriguez, V. (2011). Correlates and risk markers for psychopathology in children with and without autism spectrum disorder. *Dissertation Abstracts International: Section B: The Sciences and Engineering, 72*(2-B), 1174. Exclusion reason: Wrong patient population

Rojahn, J. (1989). Behavior Problems Inventory. In. Rojahn, J., Barnard-Brak, L., Medeiros, K., & Schroeder, S. R. (2016). Stereotyped behaviours as precursors of self-injurious behaviours: a longitudinal study with infants and toddlers at risk for developmental delay. *Journal of Intellectual Disability Research, 60*(2), 156-166. 10.1111/jir.12224. Exclusion reason: Mean age < 4 years

Rojahn, J., Barnard-Brak, L., Richman, D., Dotson, W., Medeiros, K., Wei, T., & Abby, L. (2013). Behavior problems in individuals with Cornelia de Lange Syndrome: Population-specific validation of the Behavior Problem Inventory-01. *Journal of Developmental and Physical Disabilities, 25*(5), 505-515. 10.1007/s10882-012-9329-6. Exclusion reason: Results not reported separately for children and adolescents

Rojahn, J., Borthwick-Duffy, S. A., & Jacobson, J. W. (1993). The association between psychiatric diagnoses and severe behavior problems in mental retardation. *Annals of Clinical Psychiatry, 5*(3), 163-170. Exclusion reason: Results not reported separately for children and adolescents

Rojahn, J., & Matson, J. L. (2010). Assessment and diagnosis of autism and spectrum disorders in children. *Journal of Developmental and Physical Disabilities, 22*(4), 313-315. 10.1007/s10882-010-9208-y. Exclusion reason: Theoretical article/Comment

Rojahn, J., Matson, J. L., Lott, D., Esbensen, A. J., & Smalls, Y. (2001). The Behavior Problems Inventory: An instrument for the assessment of self-injury, stereotyped behavior, and aggression/destruction in individuals with developmental disabilities. *Journal of Autism and Developmental Disorders, 31*(6), 577-588. 10.1023/A:1013299028321. Exclusion reason: Results not reported separately for children and adolescents

Rojahn, J., Matson, J. L., Mahan, S., Fodstad, J. C., Knight, C., Sevin, J. A., & Sharp, B. (2009). Cutoffs, norms, and patterns of problem behaviors in children with an ASD on the Baby and Infant Screen for Children with aUtIsm Traits (BISCUIT-Part 3). *Research in Autism Spectrum Disorders, 3*(4), 989-998. 10.1016/j.rasd.2009.06.002. Exclusion reason: Mean age < 4 years

Rojahn, J., Polster, L. M., Mulick, J. A., & Wisniewski, J. J. (1989). Reliability of the Behavior Problems Inventory. *Journal of the Multihandicapped Person, 2*(4), 283-293. 10.1007/BF01098170. Exclusion reason: Results not reported separately for children and adolescents

Rojahn, J., Rowe, E., Sharber, A., Hastings, R., Matson, J., Didden, R., . . . Dumont, E. (2012). The behavior problems inventory-short form for individuals with intellectual disabilities: Part I: Development and provisional clinical reference data. *Journal of Intellectual Disability Research, 56*(5), 527-545. 10.1111/j.1365-2788.2011.01507.x. Exclusion reason: Adult population

Rojahn, J., Rowe, E., Sharber, A., Hastings, R., Matson, J., Didden, R., . . . Dumont, E. (2012). The behavior problems inventory-short form for individuals with intellectual disabilities: Part II: Reliability and validity. *Journal of Intellectual Disability Research, 56*(5), 546-565. 10.1111/j.1365-2788.2011.01506.x. Exclusion reason: Results not reported separately for children and adolescents

Rojahn, J., Schroeder, S. R., Mayo-Ortega, L., Oyama-Ganiko, R., LeBlanc, J., Marquis, J., & Berke, E. (2013). Validity and reliability of the Behavior Problems Inventory, the Aberrant Behavior Checklist, and the Repetitive Behavior Scale-Revised among infants and toddlers at risk for intellectual or developmental disabilities: A multi-method assessment approach. *Research in Developmental Disabilities, 34*(5), 1804-1814. 10.1016/j.ridd.2013.02.024. Exclusion reason: Mean age < 4 years

Romero, M., Aguilar, J. M., Del-Rey-Mejias, A., Mayoral, F., Rapado, M., Pecina, M., . . . Lara, J. P. (2016). Psychiatric comorbidities in autism spectrum disorder: A comparative study between DSM-IV-TR and DSM-5 diagnosis. *International Journal of Clinical and Health Psychology, 16*(3), 266-275. 10.1016/j.ijchp.2016.03.001. Exclusion reason: No intellectual disability information

Rosenberg, L. A., Harris, J. C., & Reifler, J. P. (1988). Similarities and differences between parents' and teachers' observations of the behavior of children with learning problems. *Journal of Learning Disabilities, 21*(3), 189-190. 10.1177/002221948802100313. Exclusion reason: No intellectual disability information

Ruane, A., Carr, A., Moffat, V., Finn, T., Murphy, A., O'Brien, O., . . . O'Dwyer, R. (2019). A randomised controlled trial of the Group Stepping Stones Triple P training programme for parents of children with developmental disabilities. *Clinical Child Psychology & Psychiatry, 24*(4), 728-753. 10.1177/1359104519827622. Exclusion reason: Wrong patient population

Russell, A. T., Hahn, J. E., & Hayward, K. (2011). Psychiatric services for individuals with intellectual and developmental disabilities: Medication management. *Journal of Mental Health Research in Intellectual Disabilities, 4*(4), 265-289. 10.1080/19315864.2011.603107. Exclusion reason: No psychometric information

Russell, A. T., & Tanguay, P. E. (1981). Mental illness and mental retardation: Cause or coincidence? *American Journal of Mental Deficiency, 85*(6), 570-574. Exclusion reason: Not relevant outcome

Ruttle, K., & Dick, D. F. (1982). *Assessment of Behavior Disorders and Developmental Delay: Parent-Teacher Agreement*. Retrieved from http://search.ebscohost.com/login.aspx?direct=true&db=eric&AN=ED219895&site=ehost-live Exclusion reason: Not relevant outcome

http://search.ebscohost.com/login.aspx?direct=true&db=eric&AN=ED219895&site=ehost-live

Rzepecka, H., McKenzie, K., McClure, L., & Murphy, S. (2011). Sleep, anxiety and challenging behaviour in children with intellectual disability and/or autism spectrum disorder. *Research in Developmental Disabilities, 32*(6), 2758-2766. 10.1016/j.ridd.2011.05.034. Exclusion reason: No psychometric information

Salehi, P., Herzig, L., Capone, G., Lu, A., Oron, A. P., & Kim, S. J. (2018). Comparison of Aberrant Behavior Checklist profiles across Prader-Willi syndrome, Down syndrome, and autism spectrum disorder. *American Journal of Medical Genetics Part A, 176*(12), 2751-2759. 10.1002/ajmg.a.40665. Exclusion reason: No intellectual disability information

Sanchez-Teruel, D., & Robles-Bello, M. A. (2020). Preliminary Study on Psychometric Properties or an Anxiety Scale in Down Syndrome with Anxiety Symptoms. *International Journal of Psychological Research, 13*(1), 50-61. 10.21500/20112084.4493. Exclusion reason: Not relevant outcome

Sandvik, V., Hysing, M., & Lundervold, A. J. (2007). Strengths and Difficulties Questionnaire (SDQ) in assessment of psychological problems in children with mental retardation. *Tidsskrift for Norsk Psykologforening, 44*(6), 750-754. Exclusion reason: No psychometric information

Sarimski, K. (2004). Assessment of behavioral problems in children with intellectual disability: The Nisonger child behavior rating form. *Praxis der Kinderpsychologie und Kinderpsychiatrie, 53*(5), 319-332. Exclusion reason: Duplicate reference

Sarimski, K. (2004). Assessment of behavioral problems in children with intellectual disability: The Nisonger child behavior rating form. [German]. *Praxis der Kinderpsychologie und Kinderpsychiatrie, 53*(5), 319-332. Exclusion reason: Article in foreign language/not accessible language

Sarphare, G. S. (1995). Assessment of fears and anxiety in children with mental retardation: Developmental considerations. *Dissertation Abstracts International: Section B: The Sciences and Engineering, 56*(1-B), 0546. Exclusion reason: Not relevant outcome

Scahill, L., Aman, M. G., Lecavalier, L., Halladay, A. K., Bishop, S. L., Bodfish, J. W., . . . Dawson, G. (2015). Measuring repetitive behaviors as a treatment endpoint in youth with autism spectrum disorder. *Autism, 19*(1), 38-52. 10.1177/1362361313510069. Exclusion reason: Review

Scahill, L., Dimitropoulos, A., McDougle, C. J., Aman, M. G., Feurer, I. D., McCracken, J. T., . . . Vitiello, B. (2014). Children's Yale-Brown obsessive compulsive scale in autism spectrum disorder: component structure and correlates of symptom checklist. *Journal of the American Academy of Child & Adolescent Psychiatry, 53*(1), 97-107.e101. 10.1016/j.jaac.2013.09.018. Exclusion reason: Wrong patient population

Scahill, L., McDougle, C. J., Williams, S. K., Dimitropoulos, A., Aman, M. G., McCracken, J. T., . . . McMahon, D. (2006). Children's yale-brown obsessive compulsive scale modified for pervasive developmental disorders. *Journal of the American Academy of Child and Adolescent Psychiatry, 45*(9), 1114-1123. 10.1097/01.chi.0000220854.79144.e7. Exclusion reason: Not relevant measurement tool

Schmidt, J. D., Huete, J. M., Fodstad, J. C., Chin, M. D., & Kurtz, P. F. (2013). An evaluation of the Aberrant Behavior Checklist for Children under age 5. *Research in Developmental Disabilities, 34*(4), 1190-1197. 10.1016/j.ridd.2013.01.002. Exclusion reason: Mean age < 4 years

Schmidt, S. L., Snyder, T. J., Rouget, A. C., & Gray, E. (2000). Empirical analysis of the Selective Attention and Associated Behavior checklists of the Aggregate Neurobehavioral Student Health and Educational Review. *Journal of Developmental and Behavioral Pediatrics, 21*(3), 165-171. Exclusion reason: Wrong patient population

Scholte, E. M., Van Berckelaer-Onnes, I., & Van der Ploeg, J. D. (2008). Rating scale to screen symptoms of psychiatric disorders in children. *European Journal of Special Needs Education, 23*(1), 47-62. 10.1080/08856250701791286. Exclusion reason: No intellectual disability information

Schroeder, S. R., Marquis, J. G., Reese, R., Richman, D. M., Mayo-Ortega, L., Oyama-Ganiko, R., . . . Lawrence, L. (2014). Risk factors for self-injury, aggression, and stereotyped behavior among young children at risk for intellectual and developmental disabilities. *American Journal on Intellectual and Developmental Disabilities, 119*(4), 351-370. 10.1352/1944-7558-119.4.351. Exclusion reason: Mean age < 4 years

Schroeder, S. R., Marquis, J. G., Reese, R. M., Richman, D. M., Mayo-Ortega, L., Oyama-Ganiko, R., . . . Lawrence, L. (2013). Risk factors for self-injury, aggression, and stereotyped behavior among young children at risk for intellectual and developmental disabilities. *American Journal on Intellectual & Developmental Disabilities, 118*(3), 351-370. 10.1352/1944-7558-119.4.351. Exclusion reason: Wrong patient population

Schroeder, S. R., Richman, D. M., Abby, L., Courtemanche, A. B., & Oyama-Ganiko, R. (2014). Functional analysis outcomes and comparison of direct observations and informant rating scales in the assessment of severe behavior problems of infants and toddlers at-risk for developmental delays. *Journal of Developmental and Physical Disabilities, 26*(3), 325-334. 10.1007/s10882-014-9368-2. Exclusion reason: Mean age < 4 years

Schroeder, S. R., Rojahn, J., An, X., Mayo-Ortega, L., Oyama-Ganiko, R., & LeBlanc, J. (2014). The Parental Concerns Questionnaire: A brief screening instrument for potentially severe behavior problems in infants and toddlers at-risk for developmental delays. *Journal of Developmental and Physical Disabilities, 26*(2), 237-247. 10.1007/s10882-013-9359-8. Exclusion reason: Mean age < 4 years

Schum, R. (2004). Psychological Assessment of Children with Multiple Handicaps Who Have Hearing Loss. *The Volta Review, 104*(4), 237-255. Exclusion reason: Theoretical article/Comment

Scotti, J. R., Stevens, S. B., Jacoby, V. M., Bracken, M. R., Freed, R., & Schmidt, E. (2012). Trauma in people with intellectual and developmental disabilities: Reactions of parents and caregivers to research participation. *Intellectual and Developmental Disabilities, 50*(3), 199-206. 10.1352/1934-9556-50.3.199. Exclusion reason: Not relevant measurement tool

Siegel, M., Milligan, B., Chemelski, B., Payne, D., Ellsworth, B., Harmon, J., . . . Smith, K. A. (2014). Specialized inpatient psychiatry for serious behavioral disturbance in autism and intellectual disability. *Journal of Autism and Developmental Disorders, 44*(12), 3026-3032. 10.1007/s10803-014-2157-z. Exclusion reason: Wrong patient population

Siegel, M., Milligan, B., Stein, H., Teer, O., & Smith, K. A. (2013). Telephone administration of the aberrant behavior checklist: a pilot study of feasibility in children with intellectual disability and autism. *Journal of Intellectual Disabilities, 17*(3), 265-271. 10.1177/1744629513500007. Exclusion reason: Wrong patient population

Siegfried, L. W. K. (2000). A study of the reliability and validity of the chinese version aberrant behavior checklist. Hong Kong Polytechnic University, M.Sc. Retrieved from https://theses.lib.polyu.edu.hk/handle/200/4210 Exclusion reason: Wrong patient population

Sigafoos, J., Kerr, M., Roberts, D., & Couzens, D. (1993). Reliability of structured interviews for the assessment of challenging behaviour. *Behaviour Change, 10*(1), 47-50. Exclusion reason: Results not reported separately for children and adolescents

Sim, F., Haig, C., O’Dowd, J., Thompson, L., Law, J., McConnachie, A., . . . O'Dowd, J. (2015). Development of a triage tool for neurodevelopmental risk in children aged 30 months. *Research in Developmental Disabilities, 45*, 69-82. 10.1016/j.ridd.2015.07.017. Exclusion reason: Mean age < 4 years

Sim, F., Haig, C., O'Dowd, J., Thompson, L., Law, J., McConnachie, A., . . . Wilson, P. (2015). Development of a triage tool for neurodevelopmental risk in children aged 30 months. *Research in Developmental Disabilities, 45-46*, 69-82. 10.1016/j.ridd.2015.07.017. Exclusion reason: Mean age < 4 years

Singh, A. N., Matson, J. L., Mouttapa, M., Pella, R. D., Hill, B. D., & Thorson, R. (2009). A critical item analysis of the QABF: Development of a short form assessment instrument. *Research in Developmental Disabilities, 30*(4), 782-792. 10.1016/j.ridd.2008.11.001. Exclusion reason: Results not reported separately for children and adolescents

Singh, C. (2018). Assessment of anxiety in youths with Fragile X syndrome. *Dissertation Abstracts International: Section B: The Sciences and Engineering, 79*(1-B(E)), No Pagination Specified. Exclusion reason: No psychometric information

Sipes, M., Matson, J. L., Horovitz, M., & Shoemaker, M. (2011). The relationship between autism spectrum disorders and symptoms of conduct problems: The moderating effect of communication. *Developmental Neurorehabilitation, 14*(1), 54-59. 10.3109/17518423.2010.532850. Exclusion reason: Mean age < 4 years

Skwerer, D. P., Joseph, R. M., Eggleston, B., Meyer, S. R., & Tager-Flusberg, H. (2019). Prevalence and correlates of psychiatric symptoms in minimally verbal children and adolescents with ASD. *Frontiers in Psychiatry, 10 (FEB) (no pagination)*(43). 10.3389/fpsyt.2019.00043. Exclusion reason: No psychometric information

Slomka, G. T. (1987). An analysis of the application of three behavior rating scales in the assessment of mentally retarded school children. *Dissertation Abstracts International Section A: Humanities and Social Sciences, 47*(12-A, Pt 1), 4337. Exclusion reason: Conference Abstract

Slone, M., Durrheim, K., Kaminer, D., & Lachman, P. (1999). Issues in the identification of comorbidity of mental retardation and psychopathology in a multicultural context. *Social Psychiatry & Psychiatric Epidemiology, 34*(4), 190-194. Exclusion reason: No psychometric information

Sothirasan, K., Anand, A. J., Mittal, R., Khoo, P. C., & Chandran, S. (2020). Emotional and Behavioural disorders in a cohort with Down syndrome using the Strengths and Difficulties Questionnaire: A pilot study. *Heliyon, 6*(10), e05095. 10.1016/j.heliyon.2020.e05095. Exclusion reason: Results not reported separately for children and adolescents

Stadnick, N., Chlebowski, C., Baker-Ericzén, M., Dyson, M., Garland, A., & Brookman-Frazee, L. (2017). Psychiatric Comorbidity in Autism Spectrum Disorder: Correspondence between Mental Health Clinician Report and Structured Parent Interview. *Autism: The International Journal of Research and Practice, 21*(7), 841-851. Exclusion reason: Wrong patient population

Stadnick, N., Chlebowski, C., & Brookman-Frazee, L. (2017). Caregiver-Teacher Concordance of Challenging Behaviors in Children with Autism Spectrum Disorder Served in Community Mental Health Settings. *Journal of Autism and Developmental Disorders, 47*(6), 1780-1790. 10.1007/s10803-017-3101-9. Exclusion reason: Wrong patient population

Stanfield, A. C., McKechanie, A. G., Lawrie, S. M., Johnstone, E. C., & Owens, D. G. (2019). Predictors of psychotic symptoms among young people with special educational needs. *The British Journal of Psychiatry, 215*(1), 422-427. 10.1192/bjp.2018.296. Exclusion reason: Wrong patient population

Stark, K. H., Barnes, J. C., Young, N. D., & Gabriels, R. L. (2015). Brief Report: Understanding Crisis Behaviors in Hospitalized Psychiatric Patients with Autism Spectrum Disorder-Iceberg Assessment Interview. *Journal of Autism and Developmental Disorders, 45*(11), 3468-3474. 10.1007/s10803-015-2552-0. Exclusion reason: Not relevant measurement tool

Stasinos, D. P. (1984). Behavior Problem Syndromes in Educable Mentally Handicapped Greek Children: A Parent and Teacher Estimation. In. Steinhausen -Chr, H. (1985). A scale for assessing psychiatric disorders in children and adolescents. [German]. *Zeitschrift fur Kinder- und Jugendpsychiatrie, 13*(3), 230-240. Exclusion reason: Article in foreign language/not accessible language

Steinhausen, H.-C. (1985). A scale for assessing psychiatric disorders in children and adolescents. *Vol 13(3), 1985, pp 230-240, 13*(3), 230-240. Exclusion reason: Not relevant measurement tool

Steinhausen, H.-C. (1987). Global assessment of child psychopathology. *Journal of the American Academy of Child & Adolescent Psychiatry, 26*(2), 203-206. 10.1097/00004583-198703000-00014. Exclusion reason: No intellectual disability information

Steinhausen, H. C., & Metzke, C. W. (2005). The Developmental Behavior Checklist (DBC). Psychometric properties and norms of the German version. [German]. *Zeitschrift fur Klinische Psychologie und Psychotherapie, 34*(4), 266-276. 10.1026/1616-3443.34.4.266. Exclusion reason: Duplicate reference

Steinhausen, H.-C., & Metzke, C. W. (2005). The Developmental Behavior Checklist (DBC). Psychometric properties and norms of the German Version. *Zeitschrift fur Klinische Psychologie und Psychotherapie: Forschung und Praxis, 34*(4), 266-276. 10.1026/1616-3443.34.4.266. Exclusion reason: Duplicate reference

Stewart, S., Falah Hassani, K., Poss, J., & Hirdes, J. (2017). The determinants of service complexity in children with intellectual disabilities. *Journal of Intellectual Disability Research, 61*(11), 1055-1068. 10.1111/jir.12423. Exclusion reason: Not relevant measurement tool

Strohmer, D. C. P. H. T. Strohmer-Prout Behavior Rating Scale. In. Sturmey, P., Jamieson, J., Burcham, J., Shaw, B., & Bertram, L. (1996). The factor structure of the Reiss Screen for Maladaptive Behaviors in institutional and community populations. *Research in Developmental Disabilities, 17*(4), 285-291. 10.1016/0891-4222%2896%2900013-3. Exclusion reason: Adult population

Sturmey, P., Sevin, J., & Williams, D. (1995). The Behavior Problem Inventory: A further replication of its factor structure. *Journal of Intellectual Disability Research, 39*(4), 353-356. 10.1111/j.1365-2788.1995.tb00527.x. Exclusion reason: Wrong patient population

Sucuoglu, B. (2003). The psychometric characteristics of the Turkish form of the Aberrant Behavior Checklist. *Turk Psikoloji Dergisi, 18*(52), 77-91. Exclusion reason: Unable to obtain in full text

Sukhodolsky, D. G., Scahill, L., Gadow, K. D., Arnold, L., Aman, M. G., McDougle, C. J., . . . Vitiello, B. (2008). Parent-rated anxiety symptoms in children with pervasive developmental disorders: Frequency and association with core autism symptoms and cognitive functioning. *Journal of Abnormal Child Psychology, 36*(1), 117-128. 10.1007/s10802-007-9165-9. Exclusion reason: Not relevant outcome

Sullivan, K., Hooper, S., & Hatton, D. (2007). Behavioural equivalents of anxiety in children with fragile X syndrome: Parent and teacher report. *Journal of Intellectual Disability Research, 51*(1), 54-65. 10.1111/j.1365-2788.2006.00899.x. Exclusion reason: Wrong patient population

Sullivan, K. M. (2006). Behavior and emotional problems in children with fragile X syndrome: A comparison of different raters, instruments, and scoring techniques. *Dissertation Abstracts International: Section B: The Sciences and Engineering, 66*(9-B), 5074. Exclusion reason: No intellectual disability information

Swami, P. R., & Vaidya, P. M. (2015). Correlation of Self-Injurious Behaviour, Stereotyped Movements and Aggressive/ Destructive Behaviour with Sensory Processing Disorder in Children with Autism and Mental Retardation. *Indian Journal of Occupational Therapy (Indian Journal of Occupational Therapy), 47*(3), 81-88. Exclusion reason: No psychometric information

Szymanski, L., & King, B. H. (1999). Practice parameters for the assessment and treatment of children, adolescents, and adults with mental retardation and comorbid mental disorders. *Journal of the American Academy of Child & Adolescent Psychiatry, 38*(12,Suppl), 5S-31S. 10.1097/00004583-199912001-00002. Exclusion reason: Review

Taffe, J. R., Tonge, B. J., Gray, K. M., & Einfeld, S. L. (2008). Extracting more information from behaviour checklists by using components of mean based scores. *International Journal of Methods in Psychiatric Research, 17*(4), 232-240. 10.1002/mpr.260. Exclusion reason: No psychometric information

Thompson, R. J., & Curry, J. F. (1983). A construct validity study of the Missouri Children's Behavior Checklist with developmentally disabled children. *Journal of Clinical Psychology, 39*(5), 691-695. 10.1002/1097-4679%28198309%2939:5%3C691::AID-JCLP2270390508%3E3.0.CO;2-I. Exclusion reason: No intellectual disability information

Thompson, R. J., Jr., & Curry, J. F. (1985). Missouri Children's Behavior Checklist profiles with developmentally disabled children: construct validity. *Journal of Clinical Psychology, 41*(4), 556-564. Exclusion reason: Wrong patient population

Thompson, R. J., Curry, J. F., Sturner, R. A., Green, J. A., & Funk, S. G. (1982). Missouri Children's Behavior Checklist ratings of preschool children as a function of risk status for developmental and learning problems. *Journal of Pediatric Psychology, 7*(3), 307-316. 10.1093/jpepsy/7.3.307. Exclusion reason: No intellectual disability information

Thompson, R. J., Kronenberger, W., & Curry, J. F. (1989). Behavior classification system for children with developmental, psychiatric, and chronic medical problems. *Journal of Pediatric Psychology, 14*(4), 559-575. 10.1093/jpepsy/14.4.559. Exclusion reason: No intellectual disability information

Thompson, R. J., Merritt, K. A., Keith, B. R., Murphy, L. B., & Johndrow, D. A. (1992). The Missouri Children's Behavior Checklist behavioral classification system: A construct validity study with nonreferred children. *Journal of Clinical Psychology, 48*(6), 739-743. 10.1002/1097-4679%28199211%2948:6%3C739::AID-JCLP2270480607%3E3.0.CO;2-1. Exclusion reason: No intellectual disability information

Thompson, S., & Emerson, E. (1995). Inter-informant agreement on the Motivation Assessment Scale: Another failure to replicate. *Mental Handicap Research, 8*(3), 203-208. 10.1111/j.1468-3148.1995.tb00156.x. Exclusion reason: Not relevant measurement tool

Tonge, B., & Einfeld, S. (2000). The trajectory of psychiatric disorders in young people with intellectual disabilities. *Australian and New Zealand Journal of Psychiatry, 34*(1), 80-84. 10.1046/j.1440-1614.2000.00695.x. Exclusion reason: No psychometric information

Tonge, B. J., Einfeld, S. L., Krupinski, J., Mackenzie, A., McLaughlin, M., Florio, T., & Nunn, R. J. (1996). The use of factor analysis for ascertaining patterns of psychopathology in children with intellectual disability. *Journal of Intellectual Disability Research, 40*(Pt 3), 198-207. Exclusion reason: Duplicate reference

Toro, K. T., Miklosi, M., Horanyi, E., Kovacs, G. P., & Balazs, J. (2018). Reading disability spectrum: Early and late recognition, subthreshold, and full comorbidity. *Journal of Learning Disabilities, 51*(2), 158-167. 10.1177/0022219417704169. Exclusion reason: No intellectual disability information

Tosi, B., Maestro, S., & Marcheschi, M. (1995). Cognitive and affective characteristics of children with malformation syndrome. [Italian]. *Minerva Pediatrica, 47*(10), 385-392. Exclusion reason: Article in foreign language/not accessible language

Tosi, B., Maestro, S., & Marcheschi, M. (1995). [Cognitive and affective characteristics of children with malformation syndrome]. *Minerva Pediatrica, 47*(10), 385-392. Exclusion reason: Duplicate reference

Tremblay, K. N., Richer, L., Lachance, L., & Cote, A. (2010). Psychopathological manifestations of children with intellectual disabilities according to their cognitive and adaptive behavior profile. *Research in Developmental Disabilities, 31*(1), 57-69. 10.1016/j.ridd.2009.07.016. Exclusion reason: No psychometric information

Trillingsgaard, A., Damm, D., Sommer, S., Jepsen, J. R. M., Ostergaard, O., Frydenberg, M., & Thomsen, P. H. (2004). Developmental profiles on the basis of the FTF (Five to Fifteen) questionnaire: Clinical validity and utility of the FTF in a child psychiatric sample. *European Child and Adolescent Psychiatry, 13*(SUPPL.), iii39-iii49. 10.1007/s00787-004-3006-y. Exclusion reason: Results not reported separately for children and adolescents

Tsiouris, J. A., Cohen, I. L., Patti, P. J., & Korosh, W. M. (2003). Treatment of Previously Undiagnosed Psychiatric Disorders in Persons With Developmental Disabilities Decreased or Eliminated Self-Injurious Behavior. *The Journal of Clinical Psychiatry, 64*(9), 1081-1090. 10.4088/JCP.v64n0914. Exclusion reason: Results not reported separately for children and adolescents

Tucker, M. A., & Fox, R. A. (1995). Assessment of families with mildly handicapped and nonhandicapped preschoolers. *Journal of School Psychology, 33*(1), 29-37. 10.1016/0022-4405%2894%2900033-5. Exclusion reason: No intellectual disability information

Tureck, K., Matson, J. L., Cervantes, P., & Konst, M. J. (2014). An examination of the relationship between autism spectrum disorder, intellectual functioning, and comorbid symptoms in children. *Research in Developmental Disabilities, 35*(7), 1766-1772. 10.1016/j.ridd.2014.02.013. Exclusion reason: Wrong patient population

Turin, E., Grados, M. A., Tierney, E., Ferenc, L. M., Zabel, A., & Comi, A. M. (2010). Behavioral and psychiatric features of Sturge-Weber syndrome. *Journal of Nervous and Mental Disease, 198*(12), 905-913. 10.1097/NMD.0b013e3181fe75ee. Exclusion reason: No psychometric information

Turky, A., Beavis, J. M., Thapar, A. K., & Kerr, M. P. (2008). Psychopathology in children and adolescents with epilepsy: An investigation of predictive variables. *Epilepsy & Behavior, 12*(1), 136-144. 10.1016/j.yebeh.2007.08.003. Exclusion reason: No intellectual disability information

Turner, S., Sloper, P., & Knussen, C. (1991). The validity and applicability of the ADIECAS classroom rating scale in a sample of children with Down's syndrome. *Journal of Mental Deficiency Research, 35*(Pt 4), 384-391. Exclusion reason: Not relevant measurement tool

Tustin, R., Kent, P. A., Haskell, S., & Bond, M. J. (1991). Measuring severity of challenging behaviours: A behaviour disorder scale. *Australia & New Zealand Journal of Developmental Disabilities, 17*(3), 285-302. Exclusion reason: Results not reported separately for children and adolescents

Valdovinos, M. G., Zarcone, J., Hellings, J., Kim, G., & Schroeder, S. (2004). Using the Diagnostic Assessment of the Severely Handicapped-II (DASH-II) to measure the therapeutic effects of risperidone. *Journal of Intellectual Disability Research, 48*(1), 53-59. 10.1111/j.1365-2788.2004.00583.x. Exclusion reason: Wrong patient population

van den Heuvel, M., Jansen, D. E. M. C., Reijneveld, S. A., Flapper, B. C. T., & Smits-Engelsman, B. C. M. (2016). Identification of emotional and behavioral problems by teachers in children with developmental coordination disorder in the school community. *Research in Developmental Disabilities, 51-52*, 40-48. 10.1016/j.ridd.2016.01.008. Exclusion reason: Wrong patient population

van der Vaart, T., Rietman, A. B., Plasschaert, E., Legius, E., Elgersma, Y., & Moll, H. A. (2016). Behavioral and cognitive outcomes for clinical trials in children with neurofibromatosis type 1. *Neurology, 86*(2), 154-160. 10.1212/WNL.0000000000002118. Exclusion reason: Wrong patient population

van Gameren-Oosterom, H. B., Fekkes, M., van Wouwe, J. P., Detmar, S. B., Oudesluys-Murphy, A. M., & Verkerk, P. H. (2013). Problem behavior of individuals with Down syndrome in a nationwide cohort assessed in late adolescence. *The Journal of Pediatrics, 163*(5), 1396-1401. 10.1016/j.jpeds.2013.06.054. Exclusion reason: No psychometric information

Vaughn, S., Zaragoza, N., Hogan, A., & Walker, J. (1993). A four-year longitudinal investigation of the social skills and behavior problems of students with learning disabilities. *Journal of Learning Disabilities, 26*(6), 404-412. 10.1177/002221949302600606. Exclusion reason: No intellectual disability information

Verheij, C., Louwerse, A., Ende, J., Eussen, M., Gool, A., Verheij, F., . . . Greaves-Lord, K. (2015). The Stability of Comorbid Psychiatric Disorders: A 7 Year Follow Up of Children with Pervasive Developmental Disorder-Not Otherwise Specified. *Journal of Autism & Developmental Disorders, 45*(12), 3939-3948. 10.1007/s10803-015-2592-5. Exclusion reason: Wrong patient population

Viola, L., Garrido, G., & Rescorla, L. (2011). Testing multicultural robustness of the Child Behavior Checklist in a national epidemiological sample in Uruguay. *Journal of Abnormal Child Psychology, 39*(6), 897-908. 10.1007/s10802-011-9500-z. Exclusion reason: Wrong patient population

Vitiello, B., Spreat, S., & Behar, D. (1989). Obsessive-compulsive disorder in mentally retarded patients. *Journal of Nervous and Mental Disease, 177*(4), 232-236. 10.1097/00005053-198904000-00007. Exclusion reason: Results not reported separately for children and adolescents

Vlissides, N., Beail, N., Jackson, T., Williams, K., & Golding, L. (2017). Development and psychometric properties of the Psychological Therapies Outcome Scale - Intellectual Disabilities (PTOS-ID). *Journal of Intellectual Disability Research, 61*(6), 549-559. 10.1111/jir.12361. Exclusion reason: Results not reported separately for children and adolescents

Vorstman, J. A. S., Morcus, M. E. J., Duijff, S. N., Klaassen, P. W. J., Heineman-De Boer, J. A., Beemer, F. A., . . . Van Engeland, H. (2006). The 22q11.2 deletion in children: High rate of autistic disorders and early onset of psychotic symptoms. *Journal of the American Academy of Child and Adolescent Psychiatry, 45*(9), 1104-1113. 10.1097/01.chi.0000228131.56956.c1. Exclusion reason: No intellectual disability information

Walsh, K. K., & Shenouda, N. (1999). Correlations among the Reiss Screen, the Adaptive Behavior Scale part II, and the Aberrant Behavior Checklist. *American Journal on Mental Retardation, 104*(3), 236-248. 10.1352/0895-8017%281999%29104%3C0236:CATRST%3E2.0.CO;2. Exclusion reason: Adult population

Walz, N. C. (2000). Patterns of emotional and behavioral difficulties in children and adolescents with neurogenetic mental retardation syndromes. *Dissertation Abstracts International: Section B: The Sciences and Engineering, 60*(12-B), 6388. Exclusion reason: No psychometric information

Warner, G., Moss, J., Smith, P., & Howlin, P. (2014). Autism characteristics and behavioural disturbances in ~ 500 children with Down's syndrome in England and Wales. *Autism Research, 7*(4), 433-441. 10.1002/aur.1371. Exclusion reason: No psychometric information

Weisbrot, D. M., Gadow, K. D., DeVincent, C. J., & Pomeroy, J. (2005). The Presentation of Anxiety in Children with Pervasive Developmental Disorders. *Journal of Child and Adolescent Psychopharmacology, 15*(3), 477-496. 10.1089/cap.2005.15.477. Exclusion reason: No intellectual disability information

Wheeler, A., Raspa, M., Bann, C., Bishop, E., Hessl, D., Sacco, P., & Bailey, D. B., Jr. (2014). Anxiety, attention problems, hyperactivity, and the Aberrant Behavior Checklist in fragile X syndrome. *American Journal of Medical Genetics. Part A, 164A*(1), 141-155. 10.1002/ajmg.a.36232. Exclusion reason: Results not reported separately for children and adolescents

Wieland, J., & Zitman, F. G. (2016). Brief Symptom Inventory symptom profiles of outpatients with borderline intellectual functioning and major depressive disorder or posttraumatic stress disorder: Comparison with patients from regular mental health care and patients with Mild Intellectual Disabilities. *Research in Developmental Disabilities, 51-52*, 153-159. 10.1016/j.ridd.2016.01.007. Exclusion reason: Results not reported separately for children and adolescents

Will, M. N., & Wilson, B. J. (2014). A longitudinal analysis of parent and teacher ratings of problem behavior in boys with and without developmental delays. *Journal of Intellectual Disabilities, 18*(2), 176-187. 10.1177/1744629514528828. Exclusion reason: No psychometric information

Williams, J. K. (1994). Behavioral characteristics of children with Turner syndrome and children with learning disabilities. *Western Journal of Nursing Research, 16*(1), 26-35; discussion 35. Exclusion reason: Wrong patient population

Williams, J. K. (1994). Behavioral characteristics of children with Turner syndrome and children with learning disabilities... including commentary by Schepp KG and Tiedeman ME with author response. *Western Journal of Nursing Research, 16*(1), 26-39. Exclusion reason: Wrong patient population

Wilson, J. M., & Kiessling, L. S. (1988). What is measured by the Conners' Teacher Behavior Rating Scale? Replication of factor analysis. *Journal of Developmental and Behavioral Pediatrics, 9*(5), 271-278. 10.1097/00004703-198810000-00005. Exclusion reason: Wrong patient population

Witwer, A. N., Lecavalier, L., & Norris, M. (2012). Reliability and Validity of the Children's Interview for Psychiatric Syndromes-Parent Version in Autism Spectrum Disorders. *Journal of Autism and Developmental Disorders, 42*(9), 1949-1958. 10.1007/s10803-012-1442-y. Exclusion reason: No intellectual disability information

Wolf, T. M., & Wenzl, P. A. (1982). Assessment of relationship among measures of social competence and cognition in educable mentally retarded-emotionally disturbed students. *Psychological Reports, 50*(3, Pt 1), 695-700. 10.2466/pr0.1982.50.3.695. Exclusion reason: Not relevant outcome

Zeilinger, E. L., Weber, G., & Haveman, M. J. (2011). Psychometric properties and norms of the German ABC-Community and PAS-ADD Checklist. *Research in Developmental Disabilities, 32*(6), 2431-2440. 10.1016/j.ridd.2011.07.017. Exclusion reason: Adult population

## Appendix II Search strategies

Date of search: 21st of February 2020

Date of updated search 13th of March 2021. The search was for the outcome measures included from the 2020 search, in Ovid databases limited to publication date since the first search.

Searches for ongoing and unpublished trials 16th of May 2021

Information specialist: Brynhildur Axelsdottir baxelsdottir@gmail.com

Total number of hits from all databases: 31.842

Additional records identified through other sources: 6

Total number of hits after removing duplicates: 20.850

PsycINFO < 1967 to February Week 2 2020 > (Ovid interface)

**Table:**

#	Searches
1	intellectual development disorder/or anencephaly/or crying cat syndrome/or down's syndrome/or adaptive behavior/or cognitive impairment/or fetal alcohol syndrome/or fragile x syndrome/or prader willi syndrome/or rett syndrome/or savants/or williams syndrome/or developmental disabilities/or specific language impairment/or learning disorders/or exp learning disabilities/
2	((intellectual* or mental* or developmental* or learning* or cognit*) adj3 (disab* or impair* or handicap* or disorder* or subnormal* or deficien* or difficult*)).ti,ab
3	(retard* or rett* or prader willi or fragile X or Crying cat or cri du chat or savants or William* syndrome* or (down* adj2 syndrome*)).ti,ab
4	1 or 2 or 3
5	mental disorders/or child psychopathology/or adolescent psychopathology/or dual diagnosis/or comorbidity/or exp behavior problems/or behavior disorders/or conduct disorder/or exp disruptive behavior disorders/or emotional disturbances/
6	((mental* or emotional* or psych*) adj2 (disorder* or disturbance* or ill* or well-being or health* or disease* or abnormal* or patholog* or problem* or condition*)).ti,ab
7	((behavi* or conduct* or anger) adj3 (problem* or disorder*)).ti,ab
8	5 or 6 or 7
9	(childhood birth 12 yrs or adolescence 13 17 yrs).ag
10	(child* or kid or kids* or minors* or juvenil* or adoles* or youth* or youngster* or teen* or preteen* or boy or boys* or girl* or pediatr* or paediatr*).ti,ab,id
11	9 or 10
12	4 and 8 and 11
13	measurement/or exp psychometrics/or psychological assessment/or behavioral assessment/or cognitive assessment/or "communication and language measures"/or "mental health and illness assessment"/or psychosocial assessment/or structured clinical interview/
14	(psychometric* or instrument* or inventor* or self-report* or validat* or validity or reliab* or norm or norms or (measurement* adj tool*)).ti,ab
15	13 or 14
16	4 and 8 and 12
17	15 and 16
18	limit 17 to yr = "1980 -Current"

Ovid MEDLINE(R) and Epub Ahead of Print, In-Process & Other Non-Indexed Citations, Daily and Versions(R) < 1946 to February 19, 2020 > (Ovid interface)

**Table:**

#	Searches
1	intellectual disability/or cri-du-chat syndrome/or de lange syndrome/or down syndrome/or mental retardation, x-linked/or prader-willi syndrome/or rubinstein-taybi syndrome/or trisomy 13 syndrome/or wagr syndrome/or williams syndrome/or Developmental Disabilities/or exp Learning Disabilities/or Specific Language Disorder/
2	((intellectual* or mental* or developmental* or learning* or cognit*) adj3 (disab* or impair* or handicap* or disorder* or subnormal* or deficien* or difficult*)).ti,ab
3	(retard* or rett* or prader willi or fragile X or Crying cat or cri du chat or savants or William* syndrome* or (down* adj2 syndrome*)).ti,ab
4	1 or 2 or 3
5	Mental disorders/or "disruptive, impulse control, and conduct disorders"/or Child Behavior Disorders/or "Diagnosis, Dual (Psychiatry)"/or Problem Behavior/or "attention deficit and disruptive behavior disorders"/
6	((mental* or emotional* or psych*) adj2 (disorder* or disturbance* or ill* or well-being or health* or disease* or abnormal* or patholog* or problem* or condition*)).ti,ab
7	((behavi* or conduct* or anger) adj3 (problem* or disorder*)).ti,ab
8	5 or 6 or 7
9	exp Child/or exp Adolescent/or Minors/or exp Pediatrics/
10	(child* or kid or kids* or minors* or juvenil* or adoles* or youth* or youngster* or teen* or preteen* or boy or boys* or girl* or pediatr* or paediatr*).ti,ab,id
11	9 or 10
12	4 and 8 and 11
13	exp Psychiatric Status Rating Scales/or psychological tests/or behavior rating scale/or psychometrics/
14	(psychometric* or instrument* or inventor* or self-report* or validat* or validity or reliab* or norm or norms or (measurement* adj tool*)).ti,ab
15	13 or 14
16	12 and 15
17	limit 16 to yr = "1980 -Current"

Embase < 1980 to 2020 Week 07 > (Ovid interface)

**Table:**

#	Searches
1	mental deficiency/or mental retardation malformation syndrome/or down syndrome/or de lange syndrome/or fragile x syndrome/or prader willi syndrome/or williams beuren syndrome/or x linked mental retardation/or wagr syndrome/or cat cry syndrome/or developmental disorder/or learning disorder/or language disability/
2	((intellectual* or mental* or developmental* or learning* or cognit*) adj3 (disab* or impair* or handicap* or disorder* or subnormal* or deficien* or difficult*)).ti,ab
3	(retard* or rett* or prader willi or fragile X or Crying cat or cri du chat or savants or William* syndrome* or (down* adj2 syndrome*)).ti,ab
4	1 or 2 or 3
5	mental disease/or comorbidity/or behavior disorder/or disruptive behavior/or problem behavior/or conduct disorder/or emotional disorder/
6	((mental* or emotional* or psych*) adj2 (disorder* or disturbance* or ill* or well-being or health* or disease* or abnormal* or patholog* or problem* or condition*)).ti,ab
7	((behavi* or conduct* or anger) adj3 (problem* or disorder*)).ti,ab
8	5 or 6 or 7
9	exp child/or exp adolescent/or exp adolescence/or exp childhood/or exp pediatrics/
10	(child* or kid or kids* or minors* or juvenil* or adoles* or youth* or youngster* or teen* or preteen* or boy or boys* or girl* or pediatr* or paediatr*).ti,ab,kw,hw,jx
11	9 or 10
12	4 and 8 and 11
13	psychologic assessment/or psychologic test/or mental test/or psychological interview/or psychological rating scale/or psychometry/or structured interview/
14	(psychometric* or instrument* or inventor* or self-report* or validat* or validity or reliab* or norm or norms or (measurement* adj tool*)).ti,ab
15	13 or 14
16	12 and 15

Health and Psychosocial Instruments < 1985 to January 2020 > (Ovid interface)

**Table:**

#	Searches
1	((intellectual* or mental* or developmental* or learning* or cognit*) adj3 (disab* or impair* or handicap* or disorder* or subnormal* or deficien* or difficult*)).ti,ab
2	(retard* or rett* or prader willi or fragile X or Crying cat or cri du chat or savants or William* syndrome* or (down* adj2 syndrome*)).ti,ab
3	1 or 2
4	((mental* or emotional* or psych*) adj2 (disorder* or disturbance* or ill* or well-being or health* or disease* or abnormal* or patholog* or problem* or condition*)).ti,ab
5	((behavi* or conduct* or anger) adj3 (problem* or disorder*)).ti,ab
6	4 or 5
7	(child* or kid or kids* or minors* or juvenil* or adoles* or youth* or youngster* or teen* or preteen* or boy or boys* or girl* or pediatr* or paediatr*).ti,ab
8	3 and 6 and 7

CINAHL EBSCO

**Table:**

S1	(MH "De Lange Syndrome") OR (MH "Cri-Du-Chat Syndrome") OR (MH "Down Syndrome") OR (MH "Intellectual Disability + ") OR (MH "Williams Syndrome") OR (MH "WAGR Syndrome") OR (MH "Prader-Willi Syndrome")
S2	(MH "Developmental Disabilities")
S3	(MH "Learning Disorders + ")
S4	TI ((intellectual* or mental* or developmental* or learning* or cognit*) N3 (disab* or impair* or handicap* or disorder* or subnormal* or deficien* or difficult*)) OR AB ((intellectual* or mental* or developmental* or learning* or cognit*) N3 (disab* or impair* or handicap* or disorder* or subnormal* or deficien* or difficult*))
S5	TI (retard* or rett* or prader willi or fragile X or Crying cat or cri du chat or savants or William* syndrome* or (down* N2 syndrome*)) OR AB (retard* or rett* or prader willi or fragile X or Crying cat or cri du chat or savants or William* syndrome* or (down* N2 syndrome*))
S6	S1 OR S2 OR S3 OR S4 OR S5
S7	(MH "Mental Disorders")
S8	(MH "Diagnosis, Dual (Psychiatry)")
S9	(MH "Child Behavior Disorders")
S10	(MH "Disruptive Behavior")
S11	TI ((mental* or emotional* or psych*) N2 (disorder* or disturbance* or ill* or well-being or health* or disease* or abnormal* or patholog* or problem* or condition*)) OR AB ((mental* or emotional* or psych*) N2 (disorder* or disturbance* or ill* or well-being or health* or disease* or abnormal* or patholog* or problem* or condition*))
S12	TI ((behavi* or conduct* or anger) N3 (problem* or disorder*)) OR AB ((behavi* or conduct* or anger) N3 (problem* or disorder*))
S13	S7 OR S8 OR S9 OR S10 OR S11 OR S12
S14	(MH "Adolescence + ") OR (MH "Child") OR (MH "Minors (Legal)")
S15	(MH "Pediatrics")
S16	TI (child* or kid or kids* or minors* or juvenil* or adoles* or youth* or youngster* or teen* or preteen* or boy or boys* or girl* or pediatr* or paediatr*) OR AB (child* or kid or kids* or minors* or juvenil* or adoles* or youth* or youngster* or teen* or preteen* or boy or boys* or girl* or pediatr* or paediatr*)
S17	S14 OR S15 OR S16
S18	S6 AND S13 AND S17
S19	(MH "Psychometrics") OR (MH "Psychological Tests + ")
S20	(MH "Measurement Issues and Assessments")
S21	(MH "Structured Interview")
S22	TI (psychometric* or instrument* or inventor* or self-report* or validat* or validity or reliab* or norm or norms or (measurement* N tool*)) OR AB (psychometric* or instrument* or inventor* or self-report* or validat* or validity or reliab* or norm or norms or (measurement* N tool*))
S23	S19 OR S20 OR S21 OR S22
S24	S18 AND S23
S25	S18 AND S23

ERIC EBSCO

**Table:**

S1	DE "Intellectual Disability"
S2	DE "Developmental Disabilities"
S3	DE "Learning Disabilities"
S4	DE "Down Syndrome"
S5	TI ((intellectual* or mental* or developmental* or learning* or cognit*) N3 (disab* or impair* or handicap* or disorder* or subnormal* or deficien* or difficult*)) OR AB ((intellectual* or mental* or developmental* or learning* or cognit*) N3 (disab* or impair* or handicap* or disorder* or subnormal* or deficien* or difficult*))
S6	TI (retard* or rett* or prader willi or fragile X or Crying cat or cri du chat or savants or William* syndrome* or (down* N2 syndrome*)) OR AB (retard* or rett* or prader willi or fragile X or Crying cat or cri du chat or savants or William* syndrome* or (down* N2 syndrome*))
S7	S1 OR S2 OR S3 OR S4 OR S5 OR S6
S8	DE "Mental Disorders"
S9	DE "Comorbidity"
S10	DE "Multiple Disabilities"
S11	DE "Behavior Disorders"
S12	TI ((mental* or emotional* or psych*) N2 (disorder* or disturbance* or ill* or well-being or health* or disease* or abnormal* or patholog* or problem* or condition*)) OR AB ((mental* or emotional* or psych*) N2 (disorder* or disturbance* or ill* or well-being or health* or disease* or abnormal* or patholog* or problem* or condition*))
S13	TI ((behavi* or conduct* or anger) N3 (problem* or disorder*)) OR AB ((behavi* or conduct* or anger) N3 (problem* or disorder*))
S14	S8 OR S9 OR S10 OR S11 OR S12 OR S13
S15	DE "Adolescents" OR DE "Early Adolescents" OR DE "Late Adolescents"
S16	DE "Children" OR DE "Preadolescents" OR DE "Young Children"
S17	DE "Youth" OR DE "Disadvantaged Youth"
S18	DE "Pediatrics"
S19	TI (child* or kid or kids* or minors* or juvenil* or adoles* or youth* or youngster* or teen* or preteen* or boy or boys* or girl* or pediatr* or paediatr*) OR AB (child* or kid or kids* or minors* or juvenil* or adoles* or youth* or youngster* or teen* or preteen* or boy or boys* or girl* or pediatr* or paediatr*)
S20	S15 OR S16 OR S17 OR S18 OR S19
S21	S7 AND S14 AND S20
S22	DE "Psychological Testing"
S23	DE "Psychometrics"
S24	DE "Structured Interviews"
S25	DE "Measures (Individuals)"
S26	DE "Behavior Rating Scales"
S27	TI (psychometric* or instrument* or inventor* or self-report* or validat* or validity or reliab* or norm or norms or (measurement* N tool*)) OR AB (psychometric* or instrument* or inventor* or self-report* or validat* or validity or reliab* or norm or norms or (measurement* N tool*))
S28	S22 OR S23 OR S24 OR S25 OR S26 OR S27
S29	S21 AND S28

Web of Science Ebsco

# 1 TS = ((intellectual* or mental* or developmental* or learning* or cognit*) NEAR/3 (disab* or impair* or handicap* or disorder* or subnormal* or deficien* or difficult*)) 147,112

# 2 TS = (retard* or rett* or prader willi or fragile X or Crying cat or cri du chat or savants or William* syndrome* or (down* NEAR/2 syndrome*)) 30,022

# 3 #2 OR #1167,234

# 4 TS = ((mental* or emotional* or psych*) NEAR/2 (disorder* or disturbance* or ill* or well-being or health* or disease* or abnormal* or patholog* or problem* or condition*))319,377

# 5 TS = ((behavi* or conduct* or anger) NEAR/3 (problem* or disorder*))58,355

# 6 #5 OR #4356,855

# 7 TS = (child* or kid or kids* or minors* or juvenil* or adoles* or youth* or youngster* or teen* or preteen* or boy or boys* or girl* or pediatr* or paediatr*)814,572

# 8 TS = (psychometric* or instrument* or inventor* or self-report* or validat* or validity or reliab* or norm or norms or (measurement* NEAR tool*))659,485

# 9 #8 AND #7 AND #6 AND #35,435

Indexes = SSCI, A&HCI Timespan = 1980–2020

Updated search 13th of March 2021

APA PsycInfo < 2002 to March Week 1 2021 >

**Table:**

#	Searches
1	(nisonger* or aberrant* or reiss or overt or devereux or ((develop* or child*) adj behav* adj checklist*) or (strength* adj2 difficult* adj question*) or (well-being adj2 special*) or (disabilit* adj assessment*) or (challeng* adj behav* adj interview)).ti,ab
2	(dbc* or cbcl* or sdq*).ti,ab
3	1 or 2
4	intellectual development disorder/or anencephaly/or crying cat syndrome/or down's syndrome/or adaptive behavior/or cognitive impairment/or fetal alcohol syndrome/or fragile x syndrome/or prader willi syndrome/or rett syndrome/or savants/or williams syndrome/or developmental disabilities/or specific language impairment/or learning disorders/or exp learning disabilities/
5	((intellectual* or mental* or developmental* or learning* or cognit*) adj3 (disab* or impair* or handicap* or disorder* or subnormal* or deficien* or difficult*)).ti,ab
6	(retard* or rett* or prader willi or fragile X or Crying cat or cri du chat or savants or William* syndrome* or (down* adj2 syndrome*)).ti,ab
7	4 or 5 or 6
8	(childhood birth 12 yrs or adolescence 13 17 yrs).ag
9	(child* or kid or kids* or minors* or juvenil* or adoles* or youth* or youngster* or teen* or preteen* or boy or boys* or girl* or pediatr* or paediatr*).ti,ab,id
10	8 or 9
11	7 and 10
12	3 and 11
13	(202,002* or 202,003* or 202,004* or 202,005* or 202,006*or 202,007*or 202,008* or 202,009* or 202,010* or 202,011* or 202,012* or 202,101* or 202,102* or 202,103*).up
14	12 and 13

Ovid MEDLINE(R) and Epub Ahead of Print, In-Process, In-Data-Review & Other Non-Indexed Citations and Daily < 2017 to March 09, 2021 >

**Table:**

#	Searches
1	(nisonger* or aberrant* or reiss or overt or devereux or ((develop* or child*) adj behav* adj checklist*) or (strength* adj2 difficult* adj question*) or (well-being adj2 special*) or (disabilit* adj assessment*) or (challeng* adj behav* adj interview)).ti,ab
2	(dbc* or cbcl* or sdq*).ti,ab
3	1 or 2
4	intellectual disability/or cri-du-chat syndrome/or de lange syndrome/or down syndrome/or mental retardation, x-linked/or prader-willi syndrome/or rubinstein-taybi syndrome/or trisomy 13 syndrome/or wagr syndrome/or williams syndrome/or Developmental Disabilities/or exp Learning Disabilities/or Specific Language Disorder/
5	((intellectual* or mental* or developmental* or learning* or cognit*) adj3 (disab* or impair* or handicap* or disorder* or subnormal* or deficien* or difficult*)).ti,ab
6	(retard* or rett* or prader willi or fragile X or Crying cat or cri du chat or savants or William* syndrome* or (down* adj2 syndrome*)).ti,ab
7	4 or 5 or 6
8	exp Child/or exp Adolescent/or Minors/or exp Pediatrics/
9	(child* or kid or kids* or minors* or juvenil* or adoles* or youth* or youngster* or teen* or preteen* or boy or boys* or girl* or pediatr* or paediatr*).ti,ab,id
10	8 or 9
11	7 and 10
12	3 and 11
13	(2020 feb or 2020 mar or 2020 apr or 2020 may or 2020 jun or 2020 jul or 2020 aug or 2020 sep or 2020 oct or 2020 nov or 2020 dec or 2021 jan or 2021 feb or 2021 mar).dp
14	(202,002* or 202,003* or 202,004* or 202,005* or 202,006*or 202,007*or 202,008* or 202,009* or 202,010* or 202,011* or 202,012* or 202,101* or 202,102* or 202,103*).dt
15	13 or 14
16	12 and 15

Embase < 1996 to 2021 Week 09 >

**Table:**

#		Searches
1		(nisonger* or aberrant* or reiss or overt or devereux or ((develop* or child*) adj behav* adj checklist*) or (strength* adj2 difficult* adj question*) or (well-being adj2 special*) or (disabilit* adj assessment*) or (challeng* adj behav* adj interview)).ti,ab
2		(dbc* or cbcl* or sdq*).ti,ab
3		1 or 2
4		mental deficiency/or mental retardation malformation syndrome/or down syndrome/or de lange syndrome/or fragile x syndrome/or prader willi syndrome/or williams beuren syndrome/or x linked mental retardation/or wagr syndrome/or cat cry syndrome/or developmental disorder/or learning disorder/or language disability/
5		((intellectual* or mental* or developmental* or learning* or cognit*) adj3 (disab* or impair* or handicap* or disorder* or subnormal* or deficien* or difficult*)).ti,ab
6		(retard* or rett* or prader willi or fragile X or Crying cat or cri du chat or savants or William* syndrome* or (down* adj2 syndrome*)).ti,ab
7		4 or 5 or 6
8		exp child/or exp adolescent/or exp adolescence/or exp childhood/or exp pediatrics/
9		(child* or kid or kids* or minors* or juvenil* or adoles* or youth* or youngster* or teen* or preteen* or boy or boys* or girl* or pediatr* or paediatr*).ti,ab,kw,hw,jx
10		8 or 9
11		7 and 10
12		3 and 11
13		limit 12 to yr = "2020 -Current"

Searches for ongoing and unpublished trials

ClinicalTrials.gov

31 Studies found for: psychometric | Intellectual Disability OR mental health | Child (birth-17)

https://clinicaltrials.gov/ct2/results?cond=Intellectual+Disability+OR+mental+health&term=psychometric&cntry=&state=&city=&dist=&Search=Search&age=0

WHO International Clinical Trials Registry Platform (ICTRP)

https://apps.who.int/trialsearch/

intellect* OR mental*AND psyhometric* propertie* limited clinical trials in children
